# ﻿Just the tip of the iceberg: uncovering a hyperdiverse clade of African *Russula* (*Basidiomycota*, *Russulales*, *Russulaceae*) species with signs of evolutionary habitat adaptations

**DOI:** 10.3897/imafungus.16.140321

**Published:** 2025-02-17

**Authors:** Cathrin Manz, Mario Amalfi, Bart Buyck, Felix Hampe, Nourou S. Yorou, Slavomír Adamčík, Meike Piepenbring

**Affiliations:** 1 Mycology Working Group, Goethe University, Biologicum, Max-von-Laue-Str. 13, 60438 Frankfurt am Main, Germany Goethe University Frankfurt am Main Germany; 2 Meise Botanic Garden, Meise, Nieuwelaan 38, 1860 Meise, Belgium Botanic Garden Meise Meise Belgium; 3 Fédération Wallonie-Bruxelles, Service Général de l’Enseignement Universitaire et de la Recherche Scientifique, Rue A.Lavallée 1, 1080 Bruxelles, Belgium Fédération Wallonie-Bruxelles, Service Général de l’Enseignement Universitaire et de la Recherche Scientifique Bruxelles Belgium; 4 Institut de Systématique, Écologie, Biodiversité (ISYEB), Muséum national d’histoire naturelle, CNRS, Sorbonne Université, EPHE, 57 rue Cuvier, CP 39, 75005 Paris, France Muséum national d’histoire naturelle, CNRS, Sorbonne Université Paris France; 5 Wetzlarer Str. 1, 35510 Butzbach, Germany Unaffiliated Butzbach Germany; 6 Research Unit Tropical Mycology and Plant-Soil Fungi Interactions, Faculty of Agronomy, University of Parakou, Parakou, Benin University of Parakou Parakou Benin; 7 Laboratory of Molecular Ecology and Mycology, Institute of Botany, Plant Science and Biodiversity Center, Slovak Academy of Sciences, Dúbravská cesta 9, 845 23 Bratislava, Slovakia Institute of Botany, Plant Science and Biodiversity Center, Slovak Academy of Sciences Bratislava Slovakia; 8 Department of Botany, Faculty of Natural Sciences, Comenius University in Bratislava, Révová 39, 811 02 Bratislava, Slovakia Comenius University in Bratislava Bratislava Slovakia

**Keywords:** Benin, diversity estimation, gallery forests, morphological traits, phylogeny, savannah woodlands

## Abstract

The diversity within the ectomycorrhizal genus Russula (Basidiomycota) in West Africa is largely unexplored. The study area was Benin, where only ten out of the 159 species endemic to tropical Africa have been previously reported. We focused on “*Afrovirescentinae*”, which is a monophyletic lineage within Russulasubgen.Heterophyllidiaesister tosubsect.Virescentinae. The phylogenetic placement of this clade was analysed using sequence data from ITS, LSU, mtSSU, *tef1*, *rpb1* and *rpb2* regions. Ten “*Afrovirescentinae*” species are recognised, described and illustrated from Benin. Four of them, *R.carmesina*, *R.hiemisilvae*, *R.inflata* and *R.sublaevis*, were previously published. Five species, *Russulaacrialbida***sp. nov.**, *R.beenkenii***sp. nov.**, *R.coronata***sp. nov.**, *R.florae***sp. nov.** and *R.spectabilis***sp. nov.**, are newly described. Species within this group are characterised by densely reticulated spore ornamentation, but they exhibit considerable variation in field appearance and pileipellis structure. In gallery forests, their basidiomata are ephemeral, small and their basidiospores have prominent ornamentation; while in savannah woodlands, the basidiomata are fleshy, large and basidiospores present low ornamentation. We suggest that these morphological traits may represent evolutionary adaptations to a specific environmental condition. We analysed the species richness, ecological range and distribution of the “*Afrovirescentinae*” clade globally based on data from the UNITE database, estimating a total diversity of 94 species primarily distributed in sub-Saharan Africa, but also in the Neotropics. Four additional previously described species not detected in Benin were assigned to this clade, based on holotype sequencing. Several species are widely distributed across tropical Africa and do not show specificity regarding their associated plant symbionts.

## ﻿Introduction

*Russula* species are key components in ectomycorrhizal (= ECM) forests worldwide (Looney et al. 2016; Buyck et al. 2018). The genus *Russula* is one of the ten most species rich fungal genera and the second most species rich genus of ECM fungi (Bhunjun et al. 2022). Approximately 1,300 *Russula* species are described so far, but nearly 20,000 *Russula* OTUs were detected by ITS sequence data in a global dataset for soil fungi (Tedersoo et al. 2022).

Based on the analysis of environmental sequences, savannah appears to be the most diverse biome for *Russula* species (Adamčík et al. 2019). African *Russula* species occur in various vegetation types in association with plants belonging to *Asteropeiaceae*, *Dipterocarpaceae*, Fabaceae (Detarioideae), *Phyllanthaceae* or *Sarcolaenaceae* (Thoen and Bâ 1989; Ducousso et al. 2008; Tedersoo et al. 2011; Sanon et al. 2014). In tropical Africa, *Russulaceae* is the most frequently found and most abundant ECM lineage (Corrales et al. 2022) and harbours mostly generalists regarding their plant partner (Diédhiou et al. 2010; Tedersoo et al. 2011). The genus *Russula* is one of the most intensely studied genera of macrofungi in tropical Africa with 159 described species, all of which are strictly endemic to the African continent (see Suppl. material 1, Hennings (1908), overview of species described until 1992 in Buyck (1992), Härkönen et al. (1993), Buyck (1995), Buyck (1999), Härkönen et al. (2003), Buyck (2004), Buyck (2005), Buyck and Sharp (2007), Buyck (2008), Douanla-Meli and Langer (2009), Sanon et al. (2014), Wang et al. (2018), Wang et al. (2019b), Rossi et al. (2020) and Bhunjun et al. (2022)). However, little is known about the diversity of the genus in West Africa and only ten *Russula* species have been reported from Benin so far (Piepenbring et al. 2020).

The study area in Benin, West Africa, is part of the Guineo-Sudanian transition zone and harbours two major habitat types with ectomycorrhiza forming trees: 1) gallery forests with *Berliniagrandiflora* (Vahl) Hutch. & Dalziel and *Uapacaguineensis* Müll.Arg. and 2) Sudanian savannah woodlands with *Isoberlinadoka* Craib & Stapf, *Isoberliniatomentosa* (Harms) Craib & Stapf, *Monoteskerstingii* Gilg and *Uapacatogoensis* Pax. Gallery forests are characterised by a dense vegetation, water availability throughout the year and frequent flooding events during rainy seasons (Kirchmair 2017), while savannah woodlands consist of an open vegetation of more or less scattered trees and shrubs with soils covered by grasses exposed to sunlight and wind, which are frequently superficially dried out, even during the rainy season. Both habitats are widely distributed in West Africa and harbour only partially overlapping ECM fungal communities which are dominated by species of *Russulaceae* (Meidl et al. 2021). Several ECM species are reported to have broad distribution areas across sub-Saharan Africa. For example, *Cantharelluscongolensis* Beeli known from gallery forests in Benin (Dramani et al. 2022), was originally described from a lowland tropical rainforest in the Democratic Republic of the Congo (DRC) (Beeli 1928) and is reported from savannah woodlands adjacent to rainforests in Tanzania (Buyck et al. 2013). The distribution area of several ECM species occurring in Sudanian savannah woodlands in Benin extends to the miombo woodlands of the Zambezian biogeographic region (Badou et al. 2018; Han et al. 2018; Aïgnon et al. 2023). Nevertheless, community-based global studies predict at least some endemicity of ECM species in sub-Saharan Africa (Tedersoo et al. 2022).

The present study is focusing on the diversity of an underexplored lineage of mostly African Russulaspecies which is closely related tosubsect.Virescentinae Singer (Wang et al. 2019a) and is hereinafter referred to as “*Afrovirescentinae*”. Species in this lineage are morphologically highly diverse, but share a reticulate spore ornamentation and single-celled pileocystidia. They occur in gallery forests and/or savannah woodlands in Benin. Due to contrasting conditions in these two habitats, we expect corresponding morphological adaptations. These adaptations probably evolved multiple times as convergences and were facilitated by low host specificity of *Russula* spp. in Africa. Our aim was to define the species concepts and assign correct names to Beninese “*Afrovirescentinae*” members by morphological and molecular analyses of recently collected basidiomata and historical collections including holotypes. We reconstructed phylogenetic relationships within “*Afrovirescentinae*” and define the position of the group within subgen.Heterophyllidiae Romagn. based on the analysis of five DNA regions. Environmental nrITS sequence data retrieved by a PlutoF search (https://plutof.ut.ee/ accessed on 29.05.2024) were used to estimate the species diversity of “*Afrovirescentinae*”, their ecological range and distribution.

## ﻿Methods

### ﻿Sampling

In total, 283 specimens of species belonging to the genus *Russula* were collected in gallery forests and savannah woodlands in Benin during field research in June and July 2021 and 2022. The material was dried with a dehydrator at 40 °C. Molecular sequence data of the barcoding ITS nrDNA region were generated (for method, see below) and allowed a preliminary sorting of the species. Thirty-nine of the specimens from Benin were selected for this study and deposited in the Herbarium Berolinense (**B**). Additional 21 specimens including eight holotypes from various African countries were loaned and investigated from the Herbaria of the University of Parakou (**UNIPAR**), the Botanical Garden Meise (**BR**) and the University of Helsinki (**H**).

### ﻿Morphological investigation

Fresh basidiomata were photographed in the field using a Panasonic DMC-TZ81 or an Olympus TG-6 digital camera and macroscopic features were registered in the fresh state with colour codes referring to Kornerup and Wanscher (1978). Reactions to ferrous sulphate (FeSO_4_), potassium hydroxide (KOH), sulphovanillin and phenol were tested on intact tissues in the fresh state. The reaction to Guaiac was tested after 8–10 seconds on stipe and lamellar surfaces following Chalange (2014). Spore print colour codes are referring to the scale of Romagnesi (1967). Light microscopic observation and terminology follow the standards proposed by Adamčík et al. (2019) and were carried out using a Nikon eclipse 80i microscope with a Nikon Y-IDT drawing attachment at a magnification of 2,000× for line drawings and a Nikon DS-Fi2 camera for measurements from microscopic pictures. At least three collections per species were measured for microscopic descriptions, if available. The number of measured elements is indicated in brackets after the indications of the sizes for each structure. Scanning electron microscopy (SEM) was carried out using a Hitachi (S 4500) microscope with a magnification of 6,000–10,000 times.

### ﻿DNA extraction, amplification and sequencing

The genomic DNA of recently collected specimens was extracted using the innuPREP Plant DNA Kit (Analytik Jena, Germany) following the manufacturer’s instructions (protocol 1) using SLS as lysis solution. The genomic DNA of old herbarium specimens was extracted using a method developed in the context of the present study. Some 20–50 mg of dry material was placed in a 2 ml sterile Eppendorf tube. Sterile beads were added and the samples were ground using a Tissue Lyser with 2 cycles à 90 s at 1.8 Hz. 2% of PVP (polyvinylpyrrolidone) were added to the 2× cetyltrimethylammonium bromide (CTAB) buffer before the addition to the samples. The stock solution of the CTAB buffer consisted of 2% CTAB dissolved in an aqueous solution of 10% Tris-HCl 1M pH8, 28% NaCl 5M and 4% EDTA 0.5M pH 8. One ml of 55 °C hot CTAB buffer and 10 µl of proteinase K were added to each sample. The samples were incubated at 55 °C for one hour and at room temperature overnight. The next day, the samples were incubated at 65 °C for one hour. After centrifugation at room temperature at 10,000 rpm for 10 min, the supernatant liquid was transferred to a new sterile Eppendorf tube. An equal volume of isoamyl alcohol / phenol / chloroform 1:25:24 was added, then the tubes were shortly vortexed and then shaken for 2 min. After a centrifugation at room temperature at 8,000 rpm for 10 min, the upper phase was transferred to a new sterile Eppendorf tube. An amount of 2/3 of the recovered volume of -20 °C cold 2-propanol was added to the recovered upper phase, mixed by inversion for approximately 5 min and incubated at -20 °C for 15 min. After a centrifugation at room temperature at 10,000 rpm for 10 min, the supernatant was discarded. 600 µl of 70% ethanol were added to the pellet, the samples were then incubated at -20 °C for 20 min and centrifuged again at room temperature at 10,000 rpm for 2 min. The supernatant was discarded and the pellets were air dried for approx. 1 hour. The pellets were re-suspended with 75 µl 60 °C warm sterile TE-buffer and stored overnight in the fridge at 4 °C.

Six markers were amplified: 1) the internal transcribed spacer region of ribosomal DNA (ITS), comprising the ITS1 and ITS2 spacer regions and the ribosomal gene 5.8S using primers ITS1F and ITS4B (Gardes and Bruns 1993) for recently collected material; for old herbarium specimens, partial sequences were obtained using primers ITS5 and ITS2 (White et al. 1990) for the ITS1 partition or 58A1F (Martin and Rygiewicz 2005) and ITS4 (White et al. 1990) for the ITS2 partition; 2) a part of the ribosomal large subunit 28S region (LSU), using primers LR0R (Cubeta et al. 1991) and LR7 (Vilgalys and Hester 1990); 3) a part of the mitochondrial small subunit rDNA (mtSSU) using primers MS1 and MS2 (White et al. 1990); 4) the translation elongation factor 1-alpha (*tef1*) using primers *tef1*F and *tef1*R (Morehouse et al. 2003); 5) the largest subunit of the RNA polymerase II (*rpb1*) using primers gRPB1-Af (Stiller and Hall 1997) and fRPB1-Cr (Matheny et al. 2002); 6) the region between the conserved domains 6 and 7 of the second largest subunit of the RNA polymerase II (*rpb2*) using primers bRPB2-6F (Liu and Hall 2004) and bRPB2-7.1R (Matheny et al. 2005).

PCR products were obtained using a peqSTAR 2× Gradient Thermal Cycler (PEQLAB, Erlangen, Germany) and the VWR Taq DNA Polymerase (VWR, Darmstadt, Germany). The cycling conditions were as follows: For all loci, the initial denaturation was carried out for 4 min at 95 °C and all elongation steps were carried out at 72 °C. ITS & LSU: 35 cycles of denaturation for 45 s at 94 °C, annealing for 30 s at 53 °C and elongation for 60 s, final elongation for 5 min; mtSSU: 35 cycles of denaturation for 45 s at 94 °C, annealing for 45 s at 52 °C, elongation for 60 s, final elongation for 10 min; *tef1*: 35 cycles of denaturation for 45 s at 94 °C, annealing for 60 s at 52 °C, elongation for 75 s, final elongation for 10 min; *rpb1*: 20 cycles of denaturation for 45 s at 94 °C, annealing for 30 s at 55 °C (decreasing 0.5 °C each cycle), elongation for 90 s followed by 20 cycles of denaturation for 45 s at 94 °C, annealing for 30 s at 45 °C, elongation for 90 s, final elongation for 10 min; *rpb2*: 35 cycles of denaturation for 45 s at 94 °C, annealing for 60 s at 58.5 °C, elongation for 60 s, final elongation for 5 min. Successfully amplified products were sent to Microsynth Seqlab (Göttingen, Germany) for purification and sequencing using the same primers as used for PCR.

### ﻿UNITE search and analysis of ITS sequence data

The initial ITS dataset was based on sequences obtained in this study, few sequences retrieved from GenBank via BLAST search (https://blast.ncbi.nlm.nih.gov/Blast.cgi) and 13 sequences of *Heterophyllidiae* retrieved from the alignment in Wang et al. (2019a). One representative ITS sequence for each of the ten “*Afrovirescentinae*” species from Benin was used for a UNITE database search (https://unite.ut.ee/, accessed on 14.05.2024). Two approaches were tested to retrieve the maximum number of sequences belonging to “*Afrovirescentinae*” from UNITE.

1. Species hypothesis (SH) approach: For each of the ten analysed species, the massBLASTer search (https://unite.ut.ee/analysis.php, accessed on 14.05.2024) resulted in 30 most similar sequences. All unique SHs at the 1.5% dissimilarity threshold containing at least one sequence from this search were sorted. All sequences belonging to these sorted SHs were retrieved.
2. Compound cluster (CC) approach: For each of the ten species only the most similar BLAST result was selected. All corresponding SH at the 1.5% dissimilarity threshold were searched in PlutoF (https://plutof.ut.ee/, accessed on 14.05.2024) to identify the corresponding CC. All ten SH were grouped in the CC “ *Agaricomycetes* | UCL10_011118”. All sequences belonging to this CC were selected for further analysis.

The selected sequences were exported by the clipboard tool in PlutoF (https://plutof.ut.ee/) and were downloaded together with corresponding metadata (columns “Sequence ID”, “Sequence”, “Sampling area.Country”, “Identifiacation.Taxon name”, “Isolation source”, “Parent.Interactions”). The metadata were added to the sequence headers using Microsoft Excel version 2405 (Microsoft Corporation). Through repeated steps of calculating phylogenetic trees and sorting out sequences, the datasets from the two approaches were reduced to only a few sequences for each species level clade in the “*Afrovirescentinae*” representing unique information about the country of origin or ecology. In the final step, both datasets were combined into a single one used for the calculation of the phylogenetic ITS tree.

### ﻿Phylogenetic analyses

For the analysis of nr ITS data, sequences were aligned by the online version of the multiple sequence alignment programme MAFFT v. 7 (Katoh et al. 2019), using the Auto-strategy. Sequences of Russulaspecies fromsubgen.Compactae (Fr.) Bon (De Lange et al. 2021; De Lange et al. 2023) were used as an outgroup. Information on the specimens of which nr ITS sequences were newly generated for the present study are listed in Table 1. The alignment was trimmed and edited in Geneious Prime 2023.2.1 (https://www.geneious.com). A Maximum Likelihood analysis was run using the RAxML-HPC BlackBox (Stamatakis 2014) via the CIPRES Science Gateway (https://www.phylo.org/) with automatically halted bootstrapping. The final trees were edited using TreeGraph 2 (Stöver and Müller 2010) and Adobe Illustrator 27.3.

For the multigene phylogenetic analysis, 79 collections representing 55 potential species clades were included (Table 2). A combined dataset including sequence data of five partial loci (LSU, mtSSU, *tef1*, *rpb1*, *rpb2*) was constructed and used for further phylogenetics analyses. Nucleotide sequences were automatically aligned with the MUSCLE algorithm (Edgar 2004) with default settings, then manually adjusted as necessary with PhyDE® v.0.9971 (Müller et al. 2010). Potentially ambiguously aligned segments were also detected using the Gblocks v.0.91b programme (Castresana 2000) with the following parameter settings: minimum number of sequences for a conserved position = 40 (minimum possible); minimum number of sequences for a flank position = 40 (minimum possible); maximum number of contiguous non-conserved positions = 4 bp, minimum block size = 4 bp and gaps allowed within selected blocks in half of the sequences. The assignment of codon positions was confirmed by translating nucleotide sequences into predicted amino acid sequences using MacClade 4.0 (Maddison and Maddison 2005) and then compared with several annotated *Russula* sequences available on GenBank. Phylogenetic analyses were performed separately for each individual and concatenated loci using Bayesian Inference (BI) as implemented in MrBayes v.3.2 (Ronquist et al. 2011) and Maximum Likelihood (ML) as implemented in RAxML 7.0.4 (Stamatakis 2006; Stamatakis et al. 2008).

**Figure 1.**
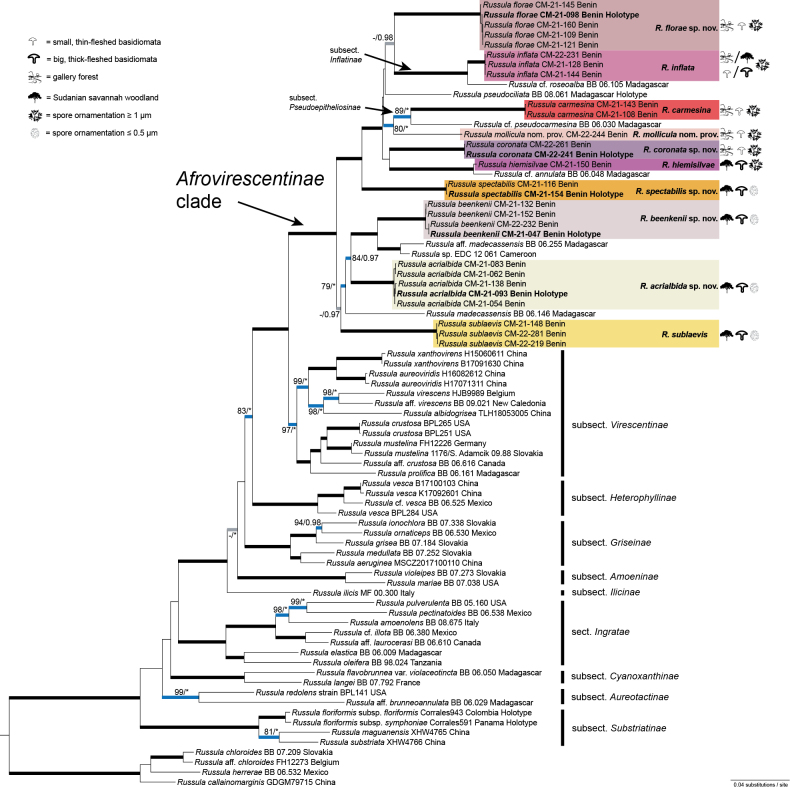
Phylogenetic Maximum Likelihood tree of Russulasubg.Heterophyllidiae, based on concatenated LSU, mtSSU, *tef 1*, *rpb1* and *rpb2* sequence data with bootstrap (ML) and Bayesian Inference (BI) values indicated over the branches. Branches in bold are supported by ML ≥ 75 or BI ≥ 0.95; black – with full support by both analyses (ML = 100, BI = 1.00), blue – supported by both ML and BI, grey – supported only by one analysis, asterisk (*) indicates full support. Highlighted species are described and illustrated in this study. Labels of holotypes of newly-described species are written in bold. Habitats (gallery forests or savannah woodlands), sizes of basidiomata (small and thin-fleshed or large and thick-fleshed) and prominence of spore ornamentation (low and high) for species occurring in Benin are indicated by icons.

**Table 1.** Information on the specimens of which nrITS sequences were newly generated for the present study.

Taxon	Voucher no.	Origin	Gen Bank Acc. No.
*R.acrialbida*	CM-21-083	Benin	PQ150333
*R.acrialbida*	CM-21-062	Benin	PQ150332
*R.acrialbida*	CM-21-138	Benin	PQ150341
***R.acrialbida***	**CM-21-093**	**Benin**	**PQ150334**
*R.acrialbida*	CM-21-054	Benin	PQ150331
*R.acrialbida*	CM-21-038	Benin	PQ150325
*R.acrialbida*	CM-21-053	Benin	PQ150330
*R.acrialbida*	CM-21-049	Benin	PQ150329
*R.acrialbida*	CM-21-039	Benin	PQ150326
*R.acrialbida*	CM-21-041	Benin	PQ150327
*R.acrialbida*	CM-22-215	Benin	PQ150353
*R.acrialbida*	CM-22-175	Benin	PQ150349
***R.albofloccosa***	**Rammeloo6096**	**Burundi**	**PQ150375**
*R.beenkenii*	CM-21-132	Benin	PQ150340
*R.beenkenii*	CM-21-152	Benin	PQ150346
*R.beenkenii*	CM-22-232	Benin	PQ150355
***R.beenkenii***	**CM-21-047**	**Benin**	**PQ150328**
*R.carmesina*	CM-21-143	Benin	PQ150342
*R.carmesina*	CM-21-108	Benin	PQ150336
*R.carmesina*	SYN5103	Guinea	PQ150380
*R.coronata*	CM-22-261	Benin	PQ150357
***R.coronata***	**CM-22-241**	**Benin**	**PQ150363**
*R.florae*	CM-21-145	Benin	PQ150343
***R.florae***	**CM-21-098**	**Benin**	**PQ150335**
*R.florae*	CM-21-160	Benin	PQ150348
*R.florae*	CM-21-109	Benin	PQ150337
*R.florae*	CM-21-121	Benin	PQ150339
*R.florae*	CM-22-178	Benin	PQ150350
*R.florae*	CM-22-284	Benin	PQ150359
*R.florae*	CM-22-185	Benin	PQ150351
*R.florae*	CM-22-206	Benin	PQ150352
*R.hiemisilvae*	CM-22-150	Benin	PQ150345
***R.hiemisilvae***	**1028**	**Tanzania**	**PQ150381**
*R.inflata*	CM-22-231	Benin	PQ150362
*R.inflata*	CM-21-128	Benin	PQ150360
*R.inflata*	CM-21-144	Benin	PQ150361
*R.inflata*	Degreef90/9	DRC	PQ150373
*R.inflata*	Schreurs1738	DRC	PQ150370
*R.inflata*	Schreurs1415	DRC	PQ150369
*R.inflata*	Thoen5209	DRC	PQ150371
*R.inflata*	Thoen7647	Senegal	PQ150372
***R.inflata***	**Buyck1652**	**DRC**	**PQ150366**
*R.inflata*	Buyck1560	DRC	PQ150365
*R.inflata*	Buyck1653	DRC	PQ150367
***R.intricata***	**Schreurs1778**	**DRC**	**PQ150368**
*R.mollicula* nom. prov.	CM-22-244	Benin	PQ150356
***R.roseoalba***	**GF3024**	**DRC**	**PQ150377**
***R.roseovelata***	**Schmitz-Levecq102**	**DRC**	**PQ150374**
***R.roseoviolacea***	**Schreurs1638**	**DRC**	**PQ150378**
*R.* sp.	OAB1010	Guinea	PQ150382
*R.* sp.	OAB1004	Guinea	PQ150383
*R.* sp.	SYN5095	Guinea	PQ150384
*R.* sp.	Buyck1660	DRC	PQ150376
*R.spectabilis*	CM-21-116	Benin	PQ150338
***R.spectabilis***	**CM-21-154**	**Benin**	**PQ150347**
*R.sublaevis*	CM-21-148	Benin	PQ150344
*R.sublaevis*	CM-22-281	Benin	PQ150358
*R.sublaevis*	CM-22-219	Benin	PQ150354
*R.sublaevis*	ADK3317	Benin	PQ150364
***R.sublaevis***	**Schreurs985**	**DRC**	**PQ150379**

Note: Holotypes are highlighted in bold.

**Table 2.** Information on the DNA sequences used to reconstruct the phylogenetic tree based on concatenated LSU, mtSSU, *tef1*, *rpb1* and *rpb2* sequence data. Holotype collections are labelled with “HT”.

Taxon	Voucher no.	Origin	LSU	mtSSU	*tef1*	*rpb1*	*rpb2*	References
*R.aureoviridis*	H16082612	China	MK881920	MK882048	MN617846	–	–	Das et al. (2017)
***R.acrialbida***	**CM-21-083**	**Benin**	**PQ152097**	**PQ157909**	**PQ179296**	**PQ203937**	**PQ203916**	**this study**
***R.acrialbida***	**CM-21-062**	**Benin**	**PQ152098**	**PQ157910**	**PQ179297**	**PQ203936**	**PQ203918**	**this study**
***R.acrialbida***	**CM-21-138**	**Benin**	**PQ152099**	**PQ157907**	**PQ179294**	**PQ203935**	**PQ203919**	**this study**
***R.acrialbida* HT**	**CM-21-093**	**Benin**	**PQ152100**	**PQ157908**	**PQ179295**	**PQ203934**	**PQ203920**	**this study**
***R.acrialbida***	**CM-21-054**	**Benin**	**PQ152101**	**PQ157911**	**PQ179298**	**PQ203933**	**PQ203917**	**this study**
*R.aeruginea*	MSCZ2017100110	China	MK881944	MK882072	MT085583	MT085539	–	unpublished
R.aff.brunneoannulata	BB 06.029	Madagascar	KU237452	KU237295	KU237887	KU237602	KU237738	Buyck et al. (2018)
R.aff.chloroides	FH12273	Belgium	KT933876	–	–	KT957386	KT933947	Looney et al. (2016)
R.aff.crustosa	BB 06.616	Canada	KU237461	KU237305	KU237896	KU237612	KU237747	Buyck et al. (2018)
R.aff.laurocerasi	BB 06.610	Canada	KU237458	KU237302	KU237893	KU237609	KU237744	Buyck et al. (2018)
R.aff.madecassensis	BB 06.255	Madagascar	KU237475	KU237319	KU237906	KU237624	KU237761	Buyck et al. (2018)
R.aff.virescens	BB 09.021	New Caledonia	KU237582	KU237430	KU238009	KU237723	KU237868	Buyck et al. (2018)
*R.albidogrisea*	TLH18053005	China	MN839562	MN839610	MT085609	MT085560	MT085637	unpublished
*R.amoenolens*	BB 08.675	Italy	KU237562	KU237410	–	KU237706	KU237848	Buyck et al. (2018)
*R.aureoviridis*	H17071311	China	MN839561	MN839609	MT085608	MT085542	MT085636	unpublished
***R.beenkenii***	**CM-21-132**	**Benin**	**PQ152093**	**PQ157915**	**PQ179302**	**PQ203928**	**PQ203903**	**this study**
***R.beenkenii***	**CM-21-152**	**Benin**	**PQ152094**	**PQ157913**	**PQ179300**	–	**PQ203902**	**this study**
***R.beenkenii***	**CM-22-232**	**Benin**	**PQ152095**	**PQ157929**	**PQ179317**	**PQ203929**	**PQ203905**	**this study**
***R.beenkenii* HT**	**CM-21-047**	**Benin**	**PQ152096**	**PQ157914**	**PQ179301**	**PQ203927**	**PQ203904**	**this study**
*R.callainomarginis*	GDGM79715	China	MN839582	MN839632	MT085602	MT085535	MT085659	Song et al. (2022)
***R.carmesina***	**CM-21-143**	**Benin**	**PQ152084**	**PQ157925**	**PQ179309**	**PQ203938**	**PQ203923**	**this study**
***R.carmesina***	**CM-21-108**	**Benin**	**PQ152085**	**PQ157922**	**PQ179308**	**PQ203926**	**PQ203922**	**this study**
R.cf.annulata	BB 06.048	Madagascar	KU237470	KU237314	KU237902	–	KU237756	
R.cf.illota	BB 06.380	Mexico	KU237464	KU237308	KU237898	KU237615	KU237750	Buyck et al. (2018)
R.cf.pseudocarmesina	BB 06.030	Madagascar	KU237453	KU237296	–	KU237603	KU237739	Buyck et al. (2018)
R.cf.roseoalba	BB 06.105	Madagascar	KU237472	KU237316	–	KU237621	KU237758	Buyck et al. (2018)
R.cf.vesca	BB 06.525	Mexico	KU237465	KU237309	KU237899	KU237616	KU237751	Buyck et al. (2018)
*R.chloroides*	BB 07.209	Slovakia	KU237559	KU237407	KU237990	KU237703	KU237845	Buyck et al. (2018)
***R.coronata***	**CM-22-261**	**Benin**	**PQ152088**	**PQ157931**	**PQ179320**	**PQ203939**	**PQ203924**	**this study**
***R.coronata* HT**	**CM-22-241**	**Benin**	**PQ152089**	**PQ157934**	**PQ179321**	–	**PQ203925**	**this study**
*R.crustosa*	BPL265	USA	KT933826	–	–	KT957338	KT933898	Looney et al. (2016)
*R.crustosa*	BPL251	USA	KT933822	–	–	KT957334	KT933894	Looney et al. (2016)
*R.elastica*	BB 06.009	Madagascar	KU237451	KU237294	–	KU237601	KU237737	Buyck et al. (2018)
R.flavobrunneavar.violaceotincta	BB 06.050	Madagascar	KU237468	KU237312	KU237901	KU237619	KU237754	Buyck et al. (2018)
***R.florae***	**CM-21-145**	**Benin**	**PQ152077**	**PQ157926**	**PQ179314**	**PQ203948**	–	**this study**
***R.florae* HT**	**CM-21-098**	**Benin**	**PQ152078**	**PQ157921**	**PQ179311**	**PQ203946**	**PQ203906**	**this study**
***R.florae***	**CM-21-160**	**Benin**	**PQ152079**	**PQ157927**	**PQ179313**	**PQ203945**	**PQ203909**	**this study**
***R.florae***	**CM-21-109**	**Benin**	**PQ152080**	**PQ157923**	**PQ179310**	**PQ203947**	**PQ203907**	**this study**
***R.florae***	**CM-21-121**	**Benin**	**PQ152081**	**PQ157924**	**PQ179312**	**PQ203949**	**PQ203908**	**this study**
R.floriformissubsp.floriformis HT	Corrales943	Colombia	MT023729	MT039861	MT024552	–	MT021752	Vera et al. (2021)
R.floriformissubsp.symphoniae HT	Corrales591	Panama	MT023730	MT039862	MT024553	–	MT021753	Vera et al. (2021)
*R.grisea*	BB 07.184	Slovakia	KU237509	KU237355	KU237939	KU237659	KU237795	Buyck et al. (2018)
*R.herrerae*	BB 06.532	Mexico	KU237486	KU237330	KU237915	KU237635	KU237772	Buyck et al. (2018)
***R.hiemisilvae***	**CM-22-150**	**Benin**	**PQ152090**	**PQ157918**	**PQ179305**	**PQ203940**	**PQ203910**	**this study**
*R.ilicis*	MF 00.300	Italy	KU237595	KU237443	KU238021	KU237733	KU237880	Buyck et al. (2018)
***R.inflata***	**CM-22-231**	**Benin**	**PQ152086**	**PQ157933**	**PQ179318**	**PQ203944**	**PQ203899**	**this study**
***R.inflata***	**CM-21-128**	**Benin**	**PQ152082**	**PQ157919**	**PQ179306**	**PQ203942**	**PQ203900**	**this study**
***R.inflata***	**CM-21-144**	**Benin**	**PQ152083**	**PQ157920**	**PQ179307**	**PQ203943**	**PQ203901**	**this study**
*R.ionochlora*	BB 07.338	Slovakia	KU237508	KU237354	KU237938	KU237658	KU237794	Buyck et al. (2018)
*R.langei*	BB 07.792	France	KU237510	KU237356	KU237940	KU237660	KU237796	Buyck et al. (2018)
*R.madecassensis*	BB 06.146	Madagascar	KU237456	KU237300	KU237891	KU237607	KU237742	Buyck et al. (2018)
*R.maguanensis*	XHW4765	China	MH714537	OQ586179	MH939983	OQ579058	MH939989	Wang et al. (2019a)
*R.mariae*	BB 07.038	USA	KU237538	KU237384	KU237968	KU237687	KU237824	Buyck et al. (2018)
*R.medullata*	BB 07.252	Slovakia	KU237546	KU237392	KU237976	KU237693	KU237832	Buyck et al. (2018)
***R.mollicula* nom. prov.**	**CM-22-244**	**Benin**	**PQ152087**	**PQ157930**	**PQ179319**	**PQ203941**	**PQ203921**	**this study**
*R.mustelina*	FH12226	Germany	KT933866	–	–	KT957376	KT933937	Looney et al. (2016)
*R.mustelina*	1176/S. Adamcik 09.88	Slovakia	KU237596	KU237444	KU238022	–	KU237881	Buyck et al. (2018)
*R.oleifera*	BB 98.024	Tanzania	KU237490	KU237334	KU237919	–	KU237776	Buyck et al. (2018)
*R.ornaticeps*	BB 06.530	Mexico	KU237466	KU237310	–	KU237617	KU237752	Buyck et al. (2018)
*R.pectinatoides*	BB 06.538	Mexico	KU237462	KU237306	–	KU237613	KU237748	Buyck et al. (2018)
*R.prolifica*	BB 06.161	Madagascar	KU237455	KU237299	KU237890	KU237606	KU237741	Buyck et al. (2018)
*R.pseudociliata*	BB 08.061	Madagascar	KU237537	KU237383	KU237967	KU237686	KU237823	Buyck et al. (2018)
*R.pulverulenta*	BB 05.160	USA	KU237563	KU237411	–	KU237707	KU237849	Buyck et al. (2018)
*R.redolens*	BPL141	USA	KT933808	–	–	KT957321	KT933879	Looney et al. (2016)
*R.* sp.	EDC 12 061	Cameroon	KR364201	–	–	KR364468	KR364338	De Crop et al. (2017)
***R.spectabilis***	**CM-21-116**	**Benin**	**PQ152091**	**PQ157916**	**PQ179303**	**PQ203950**	**PQ203914**	**this study**
***R.spectabilis* HT**	**CM-21-154**	**Benin**	**PQ152092**	**PQ157917**	**PQ179304**	**PQ203951**	**PQ203915**	**this study**
***R.sublaevis***	**CM-21-148**	**Benin**	**PQ152102**	**PQ157912**	**PQ179299**	**PQ203930**	**PQ203911**	**this study**
***R.sublaevis***	**CM-22-281**	**Benin**	**PQ152103**	**PQ157932**	**PQ179316**	**PQ203932**	**PQ203913**	**this study**
***R.sublaevis***	**CM-22-219**	**Benin**	**PQ152104**	**PQ157928**	**PQ179315**	**PQ203931**	**PQ203912**	**this study**
*R.substriata*	XHW4766	China	OQ359148	OQ371394	MH939987	OQ359996	MH939993	Wang et al. (2019a), Buyck et al. (2023)
*R.vesca*	B17100103	China	MK881939	MK882067	MN617851	MT085493	–	unpublished
*R.vesca*	K17092601	China	MN839566	MN839614	MT085613	MT085494	MT085641	unpublished
*R.vesca*	BPL284	USA	KT933839	–	–	KT957351	KT933910	Looney et al. (2016)
*R.violeipes*	BB 07.273	Slovakia	KU237534	KU237380	KU237964	KU237683	KU237820	Buyck et al. (2018)
*R.virescens*	HJB9989	Belgium	DQ422014	–	–	–	DQ421955	unpublished
*R.xanthovirens*	H15060611	China	MN839560	MN839608	MT085607	MT085501	MT085635	Song et al. (2018)
*R.xanthovirens*	B17091630	China	MK881928	MK882056	MT085570	–	–	Song et al. (2018)

Note: Newly generated data are highlighted in bold.

Russulaspecies fromsubgen.Brevipedum Buyck & V. Hofstetter (*Russulaherrerae* A. Kong, A. Montoya & Estrada strain 239/BB 06.532, *Russulachloroides* (Krombh.) Bres. strain 572/BB 07.209 and voucher FH12273 and *Russulacallainomarginis* J.F. Liang & J. Song voucher GDGM79715) were designated as outgroups. Models of evolution for BI were estimated using the Akaike Information Criterion (AIC) as implemented in ModelTest 3.7 (Posada and Crandall 1998). In order to facilitate the data partitioning by codon position, the three clade specific, confidently alignable introns, present in the *tef1* partition, in the *rpb1* partition and the one at the end of the *rpb2* partition were excised and analysed as a distinct partition.

The dataset was subdivided into eleven partitions: (LSU) (mtSSU) (*rpb1* codons 1+2) (*rpb1* 3^rd^ codon position) (*rpb2* codons 1+2) (*rpb2* codon 3) (*tef1* codons 1+2) (*tef 1* codon 3) (*tef1* introns) (*rpb1* introns) (*rpb2* intron).

The best-fit models for each partition were implemented as partition-specific models within partitioned mixed-model analyses of the combined dataset and all parameters were unlinked across partitions. The combined dataset for the Bayesian analysis was implemented with four independent runs, each with four simultaneous independent chains for 10 million generations, starting from random trees and keeping one tree every 1000^th^ generation.

All trees sampled after convergence (ave. standard deviation of split frequencies < 0.01) and confirmed using Tracer v.1.4 (Rambaut and Drummond 2007) were used to reconstruct a 50% majority-rule consensus tree (BC) and to calculate Bayesian posterior probabilities (PP). PP of each node was estimated based on the frequency at which the node was resolved amongst the sampled trees with the consensus option of 50% majority-rule (Simmons et al. 2004). A probability of 0.95 was considered significant.

Maximum Likelihood (ML) searches were conducted with RAxML involving 1000 replicates under the GTRGAMMAI model, with all model parameters estimated by the programme. In addition, 1000 bootstrap (ML BS) replicates were run with the same GTRGAMMAI model. In order to let RaxML software estimate the parameters of the evolution model separately for each independent locus, we provided an additional alignment partition file to the software. Clades with ML BS values of 75% or greater were considered supported by the data.

Nucleotide sequences are considered to be phylogenetically informative until they reach the substitution saturation; especially in coding sequences, saturation will be more pronounced in the rapidly evolving third codon position. At this point, it is no longer possible to deduce whether an observed similarity between a pair of sequences results from their common ancestry or whether this has occurred by chance (Jeffroy et al. 2006). To detect the possible bias from substitution saturation, we tested the first, second and the third codon position of the coding region studied (*tef1*, *rpb1* and *rpb2*) as well as the non-coding loci (LSU, mtSSU, *tef1*, *rpb1* and *rpb2* introns) by using Xia’s test (Xia et al. 2003; Xia and Lemey 2009), as implemented in DAMBE (Xia and Xie 2001). As the critical index substitution saturation (Iss.c) is based on simulation results, there is a problem with more than 32 species. To circumvent this problem, DAMBE was used to randomly sample subsets of 4, 8, 16 and 32 OTUs multiple times and perform the test for each subset to see if substitution saturation exists for these subsets of sequences. In order to confirm the results of the Xia’s method, we also plotted transitions and transversions at the first, second and third codon positions against Tamura-Nei genetic distances with the aid of the DAMBE package, with an asymptotic relationship indicating the presence of saturation.

Before combining the data partitions, topological incongruence between the datasets was assessed using 1000 replicates of ML BS under the same models described above, on each locus separately. Paired trees were examined for conflicts only involving nodes with ML BS > 75% (Mason-Gamer and Kellogg 1996; Lutzoni et al. 2004; Reeb et al. 2004) compared with the software compat.py (Kauff and Lutzoni 2002) available at https://www.lutzonilab.net/downloads. A conflict was assumed to be significant when two different relationships for the same set of taxa (one being monophyletic and the other non-monophyletic) were observed in rival trees.

## ﻿Results

### ﻿Phylogenetic analyses

The final combined DNA sequence alignment for the multigene phylogeny including 132 new sequences of *Russula* spp. from Benin resulted in 4,253 characters including gaps. Alignment sizes, summary statistics of sequence data, best-fit models and tests of substitution saturation for each dataset are provided in Table 3.

**Table 3.** Summary of datasets used for phylogenetic inferences.

Datasets											
Properties	LSU	mtSSU	*tef1*	*tef1*	*tef1*	*rpb1*	*rpb1*	*rpb1*	*rpb2*	*rpb2*	*rpb2*
			1^st^ & 2^nd^	3^rd^	introns	1^st^ & 2^nd^	3^rd^	introns	1^st^ & 2^nd^	3^rd^	introns
Alignment size	911	596	448	223	269	325	162	524	477	238	81
Excluded characters	-	32	-	-	-	-	-	16	-	-	11
Model selected	GTR+I+G	GTR+I+G	HKY+I+G	GTR+G	HKY+I+G	GTR+I+G	K80+I+G	GTR+I+G	GTR+I+G	GTR+G	GTR+I
-Likelihood score	4177.5352	2130.6577	1315.8429	2819.5667	3379.1914	1068.9609	2583.7498	3745.0576	1900.6295	4743.6709	1086.4247
Base frequencies											
Freq. A =	0.2569	0.3975	0.3000	0.1290	0.2473	0.3273	Equal	0.1714	0.2957	0.2038	0.2258
Freq. C =	0.2143	0.1150	0.2008	0.3705	0.2404	0.2150	Equal	0.2641	0.2369	0.2782	0.2549
Freq. G =	0.2926	0.1673	0.2738	0.2186	0.2035	0.2582	Equal	0.2484	0.2454	0.2734	0.1748
Freq. T =	0.2362	0.3201	0.2253	0.2818	0.3088	0.1995	Equal	0.3161	0.2220	0.2446	0.3445
Proportion											
of invariable sites	0.6604	0.6294	0.7879	0	0.1123	0.6222	0.1315	0.3556	0.7447	0	0.2859
Gamma shape	0.5652	0.6717	0.5475	1.9875	2.4166	0.6049	4.2319	0.5721	0.6079	2.3269	-
Test of substitution saturation											
Iss	0.155	0.206	0.318	0.505	0.539	0.265	0.465	0.439	0.278	0.518	0.661
Iss.cSym	0.741	0.702	0.696	0.686	0.692	0.684	0.709	0.703	0.700	0.684	0.999
P (Sym)	< 0.0001	< 0.0001	< 0.0001	< 0.0001	<0.001	< 0.0001	< 0.0001	< 0.0001	< 0.0001	< 0.0001	< 0.0001
Iss.cAsym	0.430	0.377	0.502	0.365	0.380	0.354	0.412	0.378	0.374	0.360	0.925
P (Asym)	< 0.0001	< 0.0001	< 0.0001	< 0.0001	< 0.0007	< 0.0001	< 0.0001	< 0.0012	< 0.0001	< 0.0003	< 0.0001

Note: Iss: index of substitution saturation. Iss.cSym: critical value for symmetrical tree topology. Iss.cAsym: critical value for extremely asymetrical tree topology. P: probability that Iss is significantly different from the critical value (Iss.cSym or Iss.cAsym).

No conflict involving significantly supported nodes was found between the tree topologies obtained for the datasets of individual loci, using the 75% ML BS criterion; the datasets were therefore combined. The test of substitution saturation showed that the observed index of substitution saturation (Iss) was significantly lower than the corresponding Iss.c in every partition, indicating that there was little saturation in our sequences (P < 0.001).

In the ML searches, the combined dataset showed 1,922 distinct patterns with a proportion of gaps and undetermined characters of 17.14%.

The two Bayesian runs converged to stable likelihood values after 605,000 generations. Therefore 9,000 stationary trees (10%) from each analysis were used to compute a 50% majority rule consensus tree and to calculate posterior probabilities (PP). The consensus tree of the BI and the ML tree were congruent as far as the terminal clades or supported lineages are concerned.

**Figure 2.**
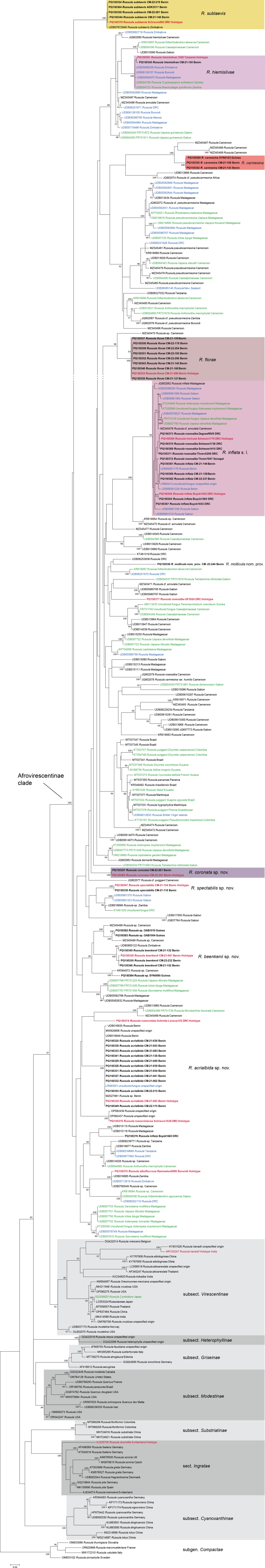
Phylogenetic Maximum Likelihood tree of Russulasubgen.Heterophyllidiae, based on nrITS sequence data. Only bootstrap values ≥ 75 are displayed. Species described and illustrated in this study are highlighted with coloured boxes. Additional species level clades are represented by sequences predominantly retrieved from UNITE. Labels of sequences generated for this study are in bold. Font colour indicates the source of the sequence: black – basidioma, red – type material, blue – soil sample, green – mycorrhizal root tip.

**Figure 3.**
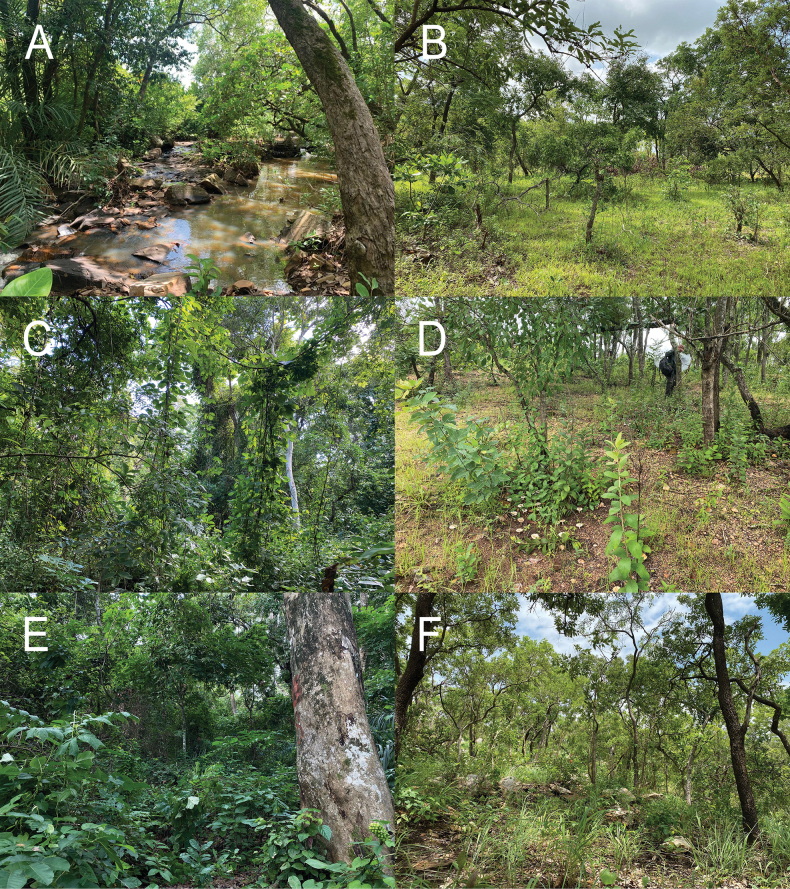
Habitats with occurrence of ECM fungi in Benin **A, C, E** gallery forests with *Berliniagrandiflora* and *Uapacaguineensis*, **B, D, F** Sudanain savannah woodlands with *Isoberliniadoka*, *Isoberliniatomentosa*, *Uapacatogoensis* and *Monoteskerstingii*.

The phylogeny showed an overall support for groups within the genus *Russula* previously recognised at section or subsection rank. The nomenclature of these infrageneric groups was assigned, based on the current taxonomic literature. All samples of tropical African species provisionally recognised as “*Afrovirescentinae*” clade were placed in a monophyletic lineage as sister to subsect.Virescentinae. Within this clade, sequences of the Beninese collections formed eight species-rank terminal clades and two additional singletons. The identity of three Beninese species was confirmed by similarity with newly-generated ITS sequences from the holotype collections: *Russulahiemisilvae* Buyck, *Russulainflata* Buyck and *Russulasublaevis* (Buyck) Buyck. The first species is represented by a single collection in the multigene phylogeny (Fig. 1), but by multiple collections in the ITS tree (Fig. 2). In addition, based on the holotype sequence, *Russulaintricata* Buyck is recognised as a synonym of *R.inflata*. The identity of *Russulacarmesina*R. Heim is verified, based on morphology after re-investigating of the holotype collection. Five other Beninese species are described as new in the present study: *R.acrialbida* sp. nov., *R.beenkenii* sp. nov., *R.coronata* sp. nov., *R.florae* sp. nov. and *R.spectabilis* sp. nov. One singleton from Benin represents an undescribed species and was labelled as “*R.mollicula* nom. prov.”. There are seven additional singletons in the multigene phylogeny representing African collections from outside Benin. The “*Afrovirescentinae*” clade included two already described subsections: *Inflatinae* Buyck and *Pseudoepitheliosinae* Buyck. Members of this clade were further grouped into two major clusters. One of them received support by both ML and BI and mostly contained species with small, thin-fleshed basidiomata with a high basidiospore ornamentation of 1.0–2.6 µm occurring in gallery forests, but also species with large and thick-fleshed basidiomata occurring in savannah woodlands. The second major cluster is supported by BI (ML = 67, BI = 0.97) only and includes species occurring in Sudanian savannah woodlands with large, thick-fleshed basidiomata and basidiospores with low basidiospore ornamentation of 0.1–0.5 µm. For Beninese collections, we observed that the species with small basidiomata almost always occur in gallery forests and species with large basidiomata exclusively occur in Sudanian savannah woodlands (Figs 1, 3). One species with basidiomata of intermediate size, *R.inflata*, occurred in both habitat types.

For the estimation of the species richness, distribution and ecological range of “*Afrovirescentinae*” species, the UNITE search applying the SH approach led to the analysis of 54 individual SH at 1.5% threshold which included 630 unique ITS sequences. The CC approach resulted in 453 retrieved ITS sequences from CC “*Agaricomycetes* | UCL10_011118”. The first approach yielded two species level clades, belonging to CC “*Russulales* | UCL10_026694”, which were not included in the dataset recovered by the CC approach. The second approach resulted in additional 17 SHs and 13 singletons not recovered by the SH approach and belonging to the “*Afrovirescentinae*” clade. The final alignment of 311 sequences for the ITS phylogeny (Fig. 2) had a length of 860 bp with 723 unique columns. The ITS tree had a topology similar to the multigene phylogeny, but with lower supports over the tree backbone. The “Afrovirescentinae” clade was well supported as sister tosubsect.Virescentinae. Compared to the multigene phylogeny, there was no support for the two major clusters within the “*Afrovirescentinae*” lineage. “*Afrovirescentinae*” include 55 supported species level clades and 39 singletons. Thus, we estimate a total diversity of approx. 94 species in the “*Afrovirescentinae*” clade, ten of which occur in Benin. Eight already described species names (*Russulaalbofloccosa* Buyck, *R.hiemisilvae*, *R.inflata*, *R.intricata*, *Russularoseoalba* Buyck, *Russularoseovelata* Buyck, *Russularoseoviolacea* Buyck, *R.sublaevis*) were recognised within the “*Afrovirescentinae*” clade by sequences from their holotypes. *Russulaalbofloccosa* and *R.roseoviolacea* were also represented by additional sequences retrieved from the UNITE database and originated from African countries other than Benin. The holotype sequences of *R.roseoalba* and *R.roseovelata* remained singletons. Three of the already-published species were confirmed to occur in Benin. *Russulacarmesina*, *R.hiemisilvae* and *R.inflata* are newly recorded for West Africa. *Russulasublaevis* is reported for the first time from Benin. Thirty-seven of the supported species clades represented by more than one sequence are only known from a single country, mostly from Cameroon or Madagascar. Four species described in this study (*R.acrialbida*, *R.coronata*, *R.florae*, *R.spectabilis*) are only known from Benin. *Russulaalbofloccosa*, *R.beenkenii*, *R.hiemisilvae*, *R.sublaevis* and at least five unidentified species only known from sequence data are broadly distributed in sub-Saharan Africa. One terminal clade contains sequences of species originating from the Neotropics that were either identified as *Russulabrasiliensis* Singer, *Russulahygrophytica* Pegler, *Russulapanamae* Buyck & Ovrebo or *Russulapuiggarii* (Speg.) Singer or remained unidentified. Besides this cluster, there are only two further singleton sequences of non-African origin from soil samples from Mexico and New Zealand.

According to data from ECM root tips, “*Afrovirescentinae*” species interact mutualistically with trees from the families *Asteropeiaceae*, Fabaceae (Detarioideae), *Phyllanthaceae* and *Sarcolaenaceae* in tropical Africa and *Dipterocarpaceae*, *Fabaceae* (*Detarioideae*, *Papilionoideae*), *Nyctaginaceae* and *Polygonacae* in the Neotropics. Several taxa, especially from Madagascar, are not specific regarding the family of their associated host tree.

### ﻿Taxonomy

#### ﻿ Russula acrialbida

Taxon classificationAnimaliaRussulalesRussulaceae

Manz, F. Hampe & Yorou, sp. nov.

0D21198F-7746-58F5-8EE9-060D6632FB75

856862

Figs 4 , 5 , 6

##### Holotype.

Benin. Collines, Toui, Forêt de Toui-Kilibo, co-ord. 8°36.4'N, 2°38.0'E, alt. 340 m, Sudanian woodland, under *Isoberliniadoka*, on sandy soil, 06.07.2021, leg. C. Manz, F. Hampe, N. S. Yorou & G. Abohoumbo, *CM-21-093* (**holotype** B 70 0105401, **isotype**UNIPAR).

##### Additional material examined.

Benin. Borgou, Wari-Maro, Forêt de Wari-Maro, co-ord. 9°11.1'N, 2°12.8'E, alt. 280 m, Sudanian woodland, under *I. doka*, on sandy soil, 30.06 2021, leg. C. Manz, F. Hampe, D. Dongnima & S. Badou, *CM-21-038* (**paratype**, B 70 0105402, UNIPAR); *ibid.* co-ord. 9°07.9'N, 2°07.5'E, alt. 340 m, Sudanian woodland, under *I. doka*, on sandy soil, 30.06 2021, leg. C. Manz, F. Hampe, D. Dongnima & S. Badou, *CM-21-039***(paratype**, B 70 0105403, UNIPAR); *ibid. CM-21-041* (**paratype**, B 70 0105404, UNIPAR); *ibid.* co-ord. 9°11.0'N, 2°12.8'E, alt. 310 m, Sudanian woodland, under *I. doka*, on sandy soil, 25.06.2022, leg. C. Manz, F. Hampe, S. Sarawi, A. Rühl & D. Dongnima, *CM-22-215* (**paratype**, B 70 0105405, UNIPAR); *ibid.* N’dail, Forêt de N’Dali, co-ord. 9°45.4'N, 2°40.1'E, alt. 360 m, Sudanian woodland, under *I. doka*, on sandy soil, 01.07.2021, leg. C. Manz, F. Hampe & G. Abohoumbo, *CM-21-049* (**paratype**, B 70 0105406, UNIPAR); *ibid.* Kpéssou, Forêt de l’Ouémé Supérieur, co-ord. 09°15.8'N, 002°11.1'E, alt. 330 m, Sudanian woodland, under *I. doka*, on sandy soil, 02.07.2021, leg. C. Manz, F. Hampe, N. S. Yorou & G. Abohoumbo, *CM-21-053* (**paratype**, B 70 0105407, UNIPAR); *ibid. CM-21-054* (**paratype**, B 70 0105408, UNIPAR); *ibid. CM-21-062* (**paratype**, B 70 0105409, UNIPAR); *ibid.* co-ord. 9°45.6'N, 2°8.0'E, alt. 320 m, Sudanian woodland, under *I. doka*, *Isoberliniatomentosa* & *Uapacatogoensis*, 18.07.2021, leg. C. Manz, F. Hampe, N. S. Yorou & G. Abohoumbo, *CM-21-138* (**paratype**, B 70 0105410, UNIPAR); *ibid.* Collines, Toui, Forêt de Toui, co-ord. 8°37.7'N, 2°35.6'E, alt. 320 m, Sudanian woodland, under *I. doka*, on sandy soil, 05.07.2021, leg. C. Manz, F. Hampe, N. S. Yorou & I. Oguchina, *CM-21-083* (**paratype**, B 70 0105411, UNIPAR); *ibid.* Atakora, Natitingou, Kota waterfalls, co-ord. 10°12.7'N, 1°26.6'E, alt. 500 m, Sudanian woodland, under *I.tomentosa*, on rocky soil, 15.06.2022, leg. C. Manz, F. Hampe, S. Sarawi, A. Rühl & D. Dongnima, *CM-22-175* (**paratype**, B 70 0105412, UNIPAR).

##### Diagnosis.

One of the most common *Russula* species in Sudanian woodlands in Benin, characterised by large white basidiomata, pileus surface with fine cream-coloured patches, burning acrid taste, small spores with a nearly smooth surface, a trichodermal pileipellis with attenuated hyphal terminations embedded in a gelatinous matrix and cystidia rapidly staining black in sulphovanillin, occurring in savannah woodlands. Differs from *R.roseovelata* by the absence of large detaching areolae on the pileus surface.

##### Description.

**Growth habit**: basidiomata solitary or in groups of up to ten. **Pileus**: mostly large to very large, rarely medium-sized, 35–155 mm in diam., when young, hemispherical, apically truncated or sometimes even slightly depressed, with margins touching the stipe, in shape similar to a matchstick head, later expanding plane, centrally depressed; margin involuted and slightly remaining so even when mature, distinctly tuberculate-striate up to 10–15 mm, frequently radially cracked, regular or slightly undulate; cuticle smooth, dry or slightly greasy when wet, finely areolate, patched towards the pileus margin, peelable up to ^1^/_2_–^3^/_4_ of the pileus radius, colour white, yellowish-white (4A2), ivory (4B2) or cream (4A3), rarely with a faint pinkish-white (9A2) hue, near the centre also orange-white (5A2), patches near the margin grey orange (5B4-5), apricot yellow (5B6), pompeian yellow (5C6), cocoa brown (6E6), cognac brown (6E7) or rusty brown (6E8) on white background. **Lamellae**: 3–8 mm wide, 8–11 lamellae present along 1 cm near the pileus margin, narrowly adnate, first white, with maturity yellow white (4A2) to cream (4A3) coloured, with frequent furcations, especially near the stipe attachment, anastomoses absent, lamellulae usually absent, only few observed in one collection, edges entire, concolourous. **Stipe**: 55–110 × 15–35 mm, cylindrical or slightly tapering towards the base, frequently bumpy or with 2–4 irregular depressions or constrictions corresponding to the distinct chambers inside, smooth to slightly rugose or with slight longitudinal ridges, annulus absent, white; cottony stuffed, cavernate with 3–5 distinct chambers. **Context**: 3–10 mm thick at half pileus radius, white, parts damaged by insects turning brownish-orange, young firm, with maturity brittle, taste burning acrid after 2–3 seconds, odour inconspicuous or sometimes slightly fruity; macrochemical reactions: guaiac after 8–10 seconds strongly positive (+++) on both stipe and lamellae surfaces, FeSO_4_ strong, deeply orange, sulphovanillin negative, sometimes bluish, KOH yellow on stipe surface and context, but negative on pileus surface, phenol negative. **Spore print**: cream (IIb-IIc).

**Spores**: (5.6–)6.2–6.6–7.1(–7.9) × (5.1–)5.4–5.7–6.1(–6.4) µm (n = 90), Q = (1.02–)1.1–1.15–1.21(–1.31), globose, subglobose to broadly ellipsoid, surface almost smooth, ornamentation very inconspicuous, composed of elements hardly visible under light microscope, ornamentation approx. 0.1 µm high as estimated by SEM, very dense weakly amyloid lines and warts forming a complete reticulum as observed by SEM, density of the individual elements not quantifiable by light microscopy; suprahilar plage small, inamyloid, covered by minute wrinkles visible only by SEM. **Basidia**: (31.5–)35.5–40–44.5(–52) × (7–)8–8.5–9(–10) µm (n = 61), clavate to broadly clavate, 4-spored; basidiola approx. 5–7 µm wide, cylindrical to clavate. **Hymenial cystidia**: on lamellae sides (56–)63–77–91(–117) × (7.5–)9–10–11(–14) µm (n = 60), moderately numerous, 1,200–1,400 cystidia/mm^2^, predominantly clavate, sometimes subcylindrical, frequently with a slightly curved base or slightly flexuose, originating in subhymenium and somewhat protruding over basidia, thin-walled, apically obtuse, usually with a 1.5–11 µm long appendage; heteromorphous contents dense, crystalline, dispersed over the entire cell, rapidly turning to dark black in sulphovanillin. Hymenial cystidia near the lamellae edges distinctly shorter and slightly narrower, (31.5–)40.5–49–57.5(–76) × (6.5–)8–9–10(–12) µm (n = 60), cylindrical to broadly clavate, occasionally with a 1–7 µm long appendage; heteromorphous contents similar to the one in hymenial cystidia on lamellae sides. **Lamellae edges**: fertile, with equal representation of cystidia, basidia, basidiola and marginal cells. **Marginal cells**: (13.5–)16.5–19.5–23(–29.5) × (2.5–)3.5–4.5–5(–7) µm (n = 62), not well differentiated, cylindrical to subclavate, sometimes slightly bent or with a secondary septum, similar to basidiola, optically empty, thin-walled. **Pileipellis**: orthochromatic in Cresyl blue, sharply delimited from the underlying context, 230–300 µm deep; suprapellis a trichoderm, 50–80 µm deep, composed of erect hyphal terminations embedded in a gelatinous matrix; gradually passing to a 170–220 µm deep, strongly gelatinised subpellis of more or less parallel, moderately dense, intricate, 2.5–3.5 µm wide hyphae and abundant cystidioid hyphae. Acid resistant incrustations absent. **Hyphal terminations**: near the pileus margin composed of (1–)2–4 unbranched cells, originating in intricate hyphae of the subpellis, thin-walled or with slightly thickened walls (up to 0.5 µm), terminal cells (10.5–)23.5–33.5–43.5(–57) × (2.5–)3–3.5–4(–5) µm (n = 90), mainly attenuated, less frequently cylindrical, apically obtuse; subterminal cells mainly shorter, 3.5–7 µm wide, cylindrical or ellipsoid. Hyphal terminations near the pileus centre of (1–)2–5 unbranched cells, thin-walled, terminal cells of similar dimensions compared to the ones near the pileus margin (11.5–)17.5–28.5–39.5(–57.5) × 2.5–3.5–4(–5) µm (n = 91), cylindrical, attenuated or subulate, often more distinctly base-inflated, sometimes apically acute; subterminal cells distinctly shorter, 3–7 µm wide, ellipsoid to ovoid, forming chains before branching. **Pileocystidia**: near the pileus margin (23.5–)32–42.5–53(–86.5) × (5.5–)7.5–9–10.5(–14) µm (n = 60), one-celled, one two-celled cystidium observed in one collection, broadly clavate, fusiform or lanceolate, originating in the suprapellis, thin-walled, apically mainly obtuse, sometimes acute, usually occasionally with a 2.5–8 µm long appendage; heteromorphous contents dense, crystalline or banded, rapidly turning to black in sulphovanillin. Pileocystidia near the pileus centre distinctly shorter, (11.5–)20–32.5–45(–62) × (4.5–)6.5–8.5–10(–13.5) µm (n = 71), globose, ovoid, ellipsoid, fusiform, clavate or lanceolate; contents similar to the one of pileocystidia near the pileus margin. **Context**: with dispersed, but distinct cystidioid hyphae, approx. 6–9.5 µm wide, sparsely septate, branched, contents dispersed or sometimes almost homogenous; oleiferous hyphae absent.

##### Etymology.

Referring to the strongly acrid taste and white colour of the basidiomata.

##### Distribution and ecology.

Only known from Sudanian woodlands dominated by *Isoberliniadoka* in Benin.

##### Notes.

*Russulaacrialbida* has spores with extremely low and fine ornamentation which is also present in *R.roseovelata*, a species with a pinkish pileipellis covered by large detaching rose-beige areolae (Fig. 7) and a pileus margin without striations (Buyck 1994). Microscopically, the latter species differs by slightly larger spores, hymenial cystidia that are up to 200 µm long and the presence of capitate terminal cells in the pileipellis with intracellular refringent transversal bands (Buyck 1994). Specimens very similar to *R.acrialbida* are illustrated and described in local African field guides as R.cf.roseovelata or *R.roseovelata* from Zambian miombo woodlands (Härkönen et al. 2015; Niemelä et al. 2021) or R.aff.roseovelata from Tanzania (Härkönen et al. 2003). It is likely that these records either represent *R.acrialbida* or a closely-related species, but probably not *R.roseovelata* s. str. because of the differences apparent in the field pictures. During our fieldwork in Benin, we encountered a man from the nearby village Wari-Maro collecting *R.acrialbida* as an edible fungus. According to him, the species would lose its acrid taste after soaking in an aqueous solution of baking soda overnight with subsequent boiling. Further studies are needed to confirm the suitability of the species for consumption as edible fungus. A similar-looking species from Tanzania identified as *R.roseovelata* is reported as edible after parboiling (Chelela et al. 2015). *Russulaalbofloccosa*, *Russulaochrocephala* Buyck and *Russulaterrena* Buyck & Sharp are tropical African species with pileus colours similar to *R.acrialbida*. *Russulaalbofloccosa* is known from Burundi and DRC and can be distinguished from *R.acrialbida* by smaller, much more fragile basidiomata, a weak reaction to FeSO_4_, a mild to weakly acrid taste and larger more elliptical spores with a more prominent ornamentation (Buyck 1994). *Russulaochrocephala* is known from DRC and Senegal and differs by spores with a more prominent ornamentation and an amyloid suprahilar plage (Buyck 1997). *Russulaterrena* is known from Zimbabwe and differs by its smaller basidiomata, weak FeSO_4_ reaction, more prominent spore ornamentation and cystidia that are insensitive to sulphovanillin (Buyck and Sharp 2007). To our knowledge, *R.acrialbida* is the only species with burning acrid taste in Russulasect.Heterophyllae Fr.

**Figure 4.**
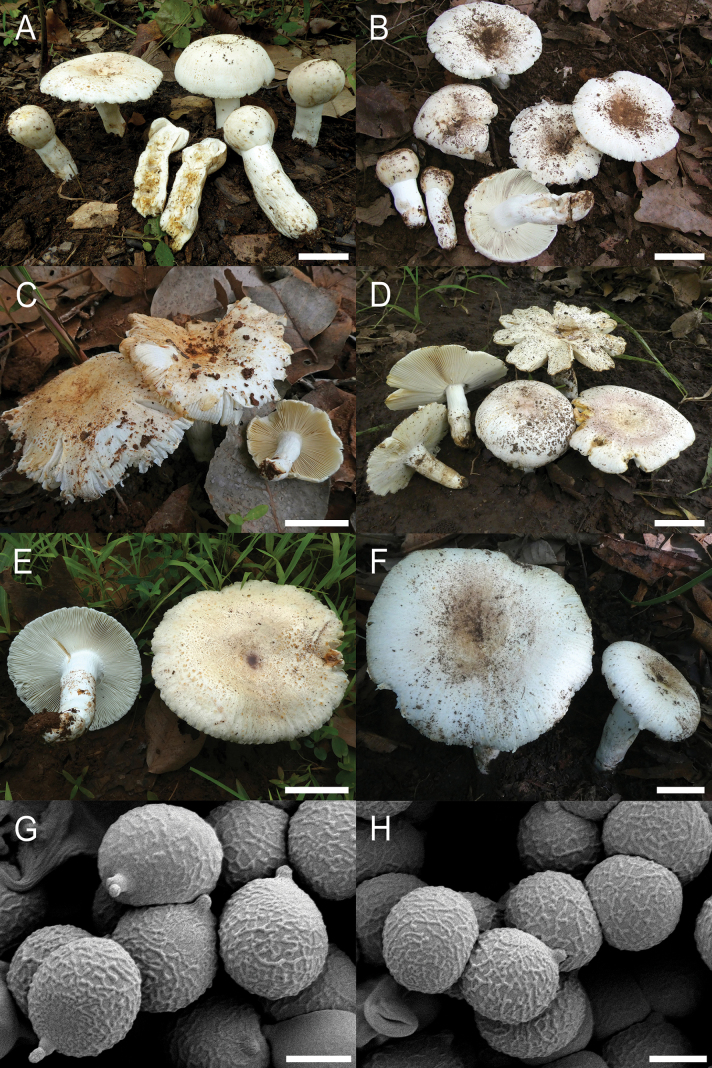
*Russulaacrialbida***A–F** field pictures of basidiomata: **A** CM-21-093, holotype, **B** CM-21-062, **C** CM-21-053, **D** CM-22-215, **E** CM-21-054, **F** CM-21-083; **G, H** scanning electron microscopical pictures of basidiospores (both CM-21-093, holotype). Scale bars: 5 cm (**A–E**); 2.5 cm (**F**); 3 µm (**G, H**).

**Figure 5.**
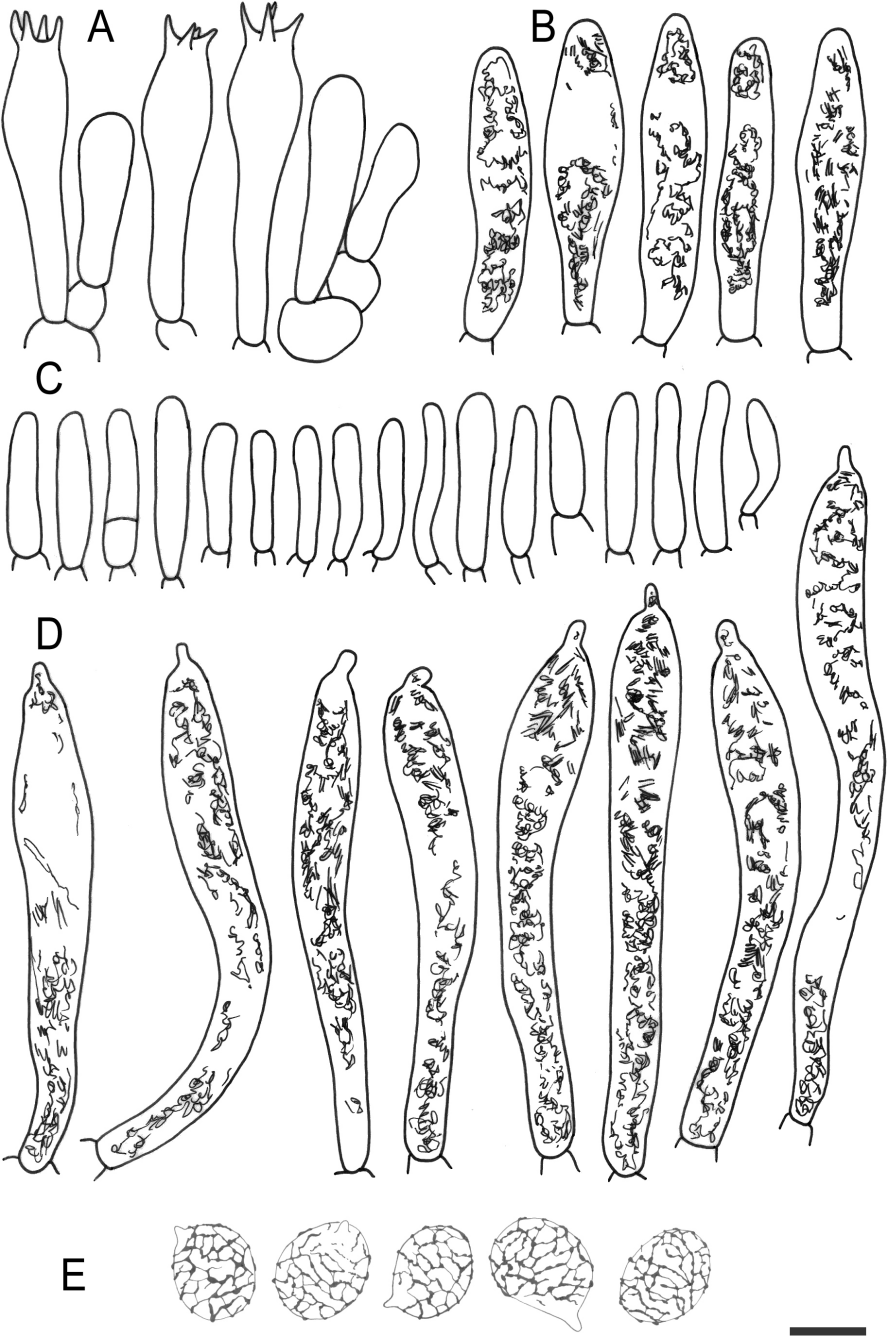
Hymenial elements of *Russulaacrialbida* (holotype, CM-21-093) **A** basidia and basidiola, **B** hymenial cystidia near the lamellar edges, **C** marginal cells, **D** hymenial cystidia, **E** spores as seen in Melzer’s reagent. Scale bar:10 μm, but only 5 μm for spores.

**Figure 6.**
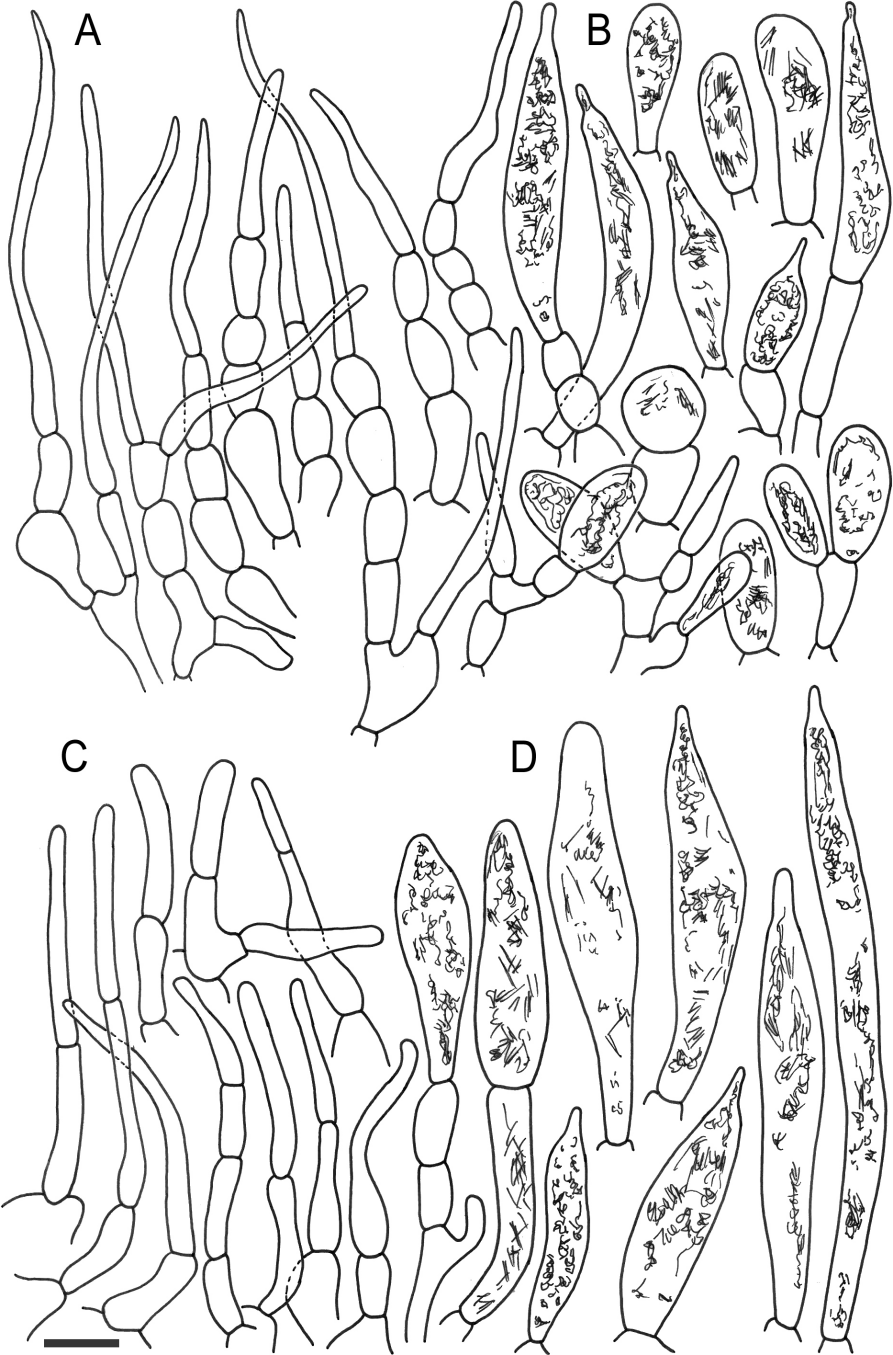
Elements of the pileipellis of *Russulaacrialbida* (holotype, CM-21-093) **A, B** elements near the pileus centre: **A** hyphal terminations, **B** pileocystidia; **C, D** elements near the pileus margin: **C** hyphal terminations, **D** pileocystidia. Scale bar: 10 μm.

#### ﻿ Russula beenkenii

Taxon classificationAnimaliaRussulalesRussulaceae

Manz, F. Hampe & M. Piepenbr., sp. nov.

AF066851-77A4-5654-8306-7A2E141EF67B

856864

Figs 8 , 9 , 10

##### Holotype.

Benin. Borgou, N’Dali, Forêt de N’Dali, co-ord. 9°45.4'N, 2°40.1'E, alt. 360 m, Sudanian woodland, under *Isoberlinadoka*, on sandy soil, 01.07.2021, leg. C. Manz, F. Hampe & G. Abohoumbo, *CM-21-047* (**holotype** B 70 0105413, **isotype**UNIPAR).

##### Additional material examined.

Benin. Borgou, Parakou, Forêt d’Okpara, co-ord. 9°14.8'N, 2°43.6'E, alt. 350 m, Sudanian woodland, under *I. doka*, *Monoteskerstingii* and *Piliostigmathonningii* (Schumach.) Milne-Redh., on sandy soil, 16.07.2021, leg. C. Manz, F. Hampe & G. Abohoumbo, *CM-21-132* (**paratype**, B 70 0105414, UNIPAR); *ibid.* Atakora, Natitingou, Kossoucoingou, co-ord. 10°9.9'N, 1°12.1'E, alt. 500 m, Sudanian woodland, under *Isoberliniatomentosa*, on rocky soil, 20.07.2021, leg. C. Manz, F. Hampe, G. Abohoumbo, T.C. Bogo, *CM-21-152* (**paratype**, B 70 0105415, UNIPAR); *ibid.* co-ord. 10°9.5'N, 1°9.5'E, alt. 330 m, Sudanian woodland, under *I.tomentosa*, on rocky soil, 27.06.2022, leg. C. Manz, S. Sarawi & A. Rühl, *CM-22-232* (**paratype**, B 70 0105416, UNIPAR).

##### Diagnosis.

Basidiomata medium-sized, pileus surface whitish with a slightly pinkish hue, mild taste, spores with a very low ornamentation, cystidia not reacting in sulphovanillin, hyphal terminations near the pileus centre very variable and with striking patches of refractive inclusions arranged in horizontal bands, occurring in savannah woodlands. Differs from *R.roseovelata* by a smooth pileus cuticle without areolae.

##### Description.

**Growth habit**: basidiomata solitary or in small groups of up to five. **Pileus**: medium-sized, 35–75 mm in diam., when young convex, later expanding plane, slightly to distinctly centrally depressed; margin even or slightly involuted, finely striate up to 10 mm, frequently radially cracked, regular or slightly undulate; cuticle smooth, matt, peelable up to ^1^/_2_–^3^/_4_ of the pileus radius, colour near the centre white, yellowish-white (4A2), ivory (4B2) or with an orange white (5A2) hue, near the margin additionally frequently with a more or less distinct pinkish-white (10-11A2) hue. **Lamellae**: 3–6 mm wide, 8–13 lamellae present along 1 cm near the pileus margin, adnexed, sometimes even free, cream-coloured, with frequent furcations near the stipe attachment, anastomoses and lamellulae absent; edges entire, concolourous. **Stipe**: 30–80 × 10–20 mm, cylindrical, sometimes bent towards the base, occasionally with 2–3 constrictions corresponding to the chambers inside, smooth to slightly rugose, annulus absent, white; cottony stuffed, cavernate with 3–4 distinct chambers. **Context**: 1–2 mm thick at half pileus radius, white, unchanging when bruised, brittle, taste mild, odour inconspicuous, macrochemical reactions: guaiac after 8–10 seconds positive (++) on both stipe and lamellae surfaces, FeSO_4_ distinctly salmon orange, sulphovanillin negative, KOH pale yellow on pileus and stipe surfaces, phenol negative. **Spore print**: not observed, but certainly not white, at least cream-coloured.

**Spores**: (5.8–)6.2–6.5–6.9(–7.5) × (4.8–)5.2–5.5–5.9(–6.4) µm (n = 120), Q = (1.07–)1.13–1.19–1.24(–1.35), subglobose to broadly ellipsoid, rarely ellipsoid; surface almost smooth, ornamentation very inconspicuous, composed of elements hardly visible by light microscopy, approx. 0.2 µm high as estimated by SEM, very dense weakly amyloid pustules and crests connected by numerous fine lines forming a complete reticulum, density of the individual elements not quantifiable by light microscopy; suprahilar plage small, inamyloid, partially covered by minute wrinkles only visible by SEM. **Basidia**: (24.5–)30.5–36–41.5(–49.5) × (6–)7.5–8.5–9.5(–10) µm (n = 60), narrowly clavate to subcylindrical, 4-spored; basidiola approx. 4–7 µm wide, cylindrical to narrowly clavate. **Hymenial cystidia**: on lamellae sides (43–)63.5–74–84.5(–100) × (6–)7.5–9–10(–11.5) µm (n = 60), subcylindrical to slightly clavate or fusiform, frequently with a slightly curved base or slightly flexuose, originating in subhymenium and somewhat protruding over basidia, thin-walled, apically obtuse, rarely acute, with a 1.5–16 µm long appendage; heteromorphous contents dense, amorphous or crystalline, sometimes located only in the upper two thirds, not reacting to sulphovanillin. Hymenial cystidia near the lamellae edges distinctly shorter, (32–)37–42–47(–52) × (5–)6–7–8(–11) µm (n = 60), cylindrical to slightly clavate or fusiform, usually with one or sometimes two (2.5–)3.5–5.5–7.5(–12) µm long appendages; heteromorphous contents slightly less dense than in cystidia on lamellae sides. **Lamellae edges**: fertile with equal representation of cystidia, basidia, basidiola and marginal cells. **Marginal cells**: (12–)19–25–31(–42) × (3–)4–5–6(–9) µm (n = 64), fusiform, lanceolate or utriform, less frequently cylindrical or subclavate and then hard to distinguish from basidiola, optically empty, thin-walled. **Pileipellis**: orthochromatic in Cresyl blue, gradually passing to the underlying context, 95–110 µm deep; suprapellis a trichoderm, 25–35 µm deep, composed of erect hyphal terminations, gelatinised; gradually passing to a 65–80 µm deep subpellis of loosely interwoven, irregularly orientated, 2.5–3 µm wide hyphae, becoming denser and horizontally orientated near the context, gelatinised in the distal part, cystidioid hyphae present. Acid resistant encrustations absent. **Hyphal terminations**: near the pileus margin composed of 1–3 unbranched cells, thin-walled, with occasional refractive inclusions, terminal cells (10–)18.5–27.5–36.5(–47) × (2.5–)3.5–4–4.5(–6) µm (n = 92), cylindrical or slightly tapering towards the apex, apically obtuse; subterminal cells distinctly shorter, 3.5–10 µm wide. Hyphal terminations near the pileus centre very variable, composed of 1–5 unbranched cells, thin-walled, often with conspicuous refractive patches, frequently arranged in a horizontal band pattern, also frequently observed in hyphae of the subpellis, terminal cells distinctly longer and slightly narrower (8–)9–22–35(–77) × (2–)2.5–3.5–4(–6.5) µm (n = 94), cylindrical or tapering towards the apex; subterminal cells shorter, 3–8 µm wide. **Pileocystidia**: near the pileus margin (21–)23.5–29.5–35.5(–43.5) × 3.5–4.5–5(–7) µm (n = 61), one-celled, predominantly lanceolate, sometimes subcylindrical, originating in the suprapellis, thin-walled, apically obtuse, with a 1–3 µm long terminal knob; heteromorphous contents moderately dense, granulose, sometimes concentrated in the apical part, also frequent in subterminal cells, not reacting to sulphovanillin. Pileocystidia near the pileus centre similar in size, shape and contents to pileocystidia near the pileus margin (17–)24.5–33–41(–61.5) × (3–)3.5–4.5–5(–6) µm (n = 60). **Context**: without cystidioid hyphae, oleiferous hyphae frequent.

##### Etymology.

After the German mycologist Ludwig Beenken, honouring his contribution to the knowledge on the diversity of the ECM morphology within the genus *Russula*.

##### Distribution and ecology.

Known from Sudanian woodlands dominated by *Isoberlinia* spp. in Benin. Distributed also in Zimbabwe.

##### Notes.

*Russulabeenkenii* has spores with extremely low ornamentation, as in *R.roseovelata*. The latter species can be distinguished by its distinctly rose coloured, areolate pileus, the absence of gelatinisation in the suprapellis, cystidia that are greying in sulphovanillin and distinctly wider pileocystidia (Buyck 1994).

**Figure 7.**
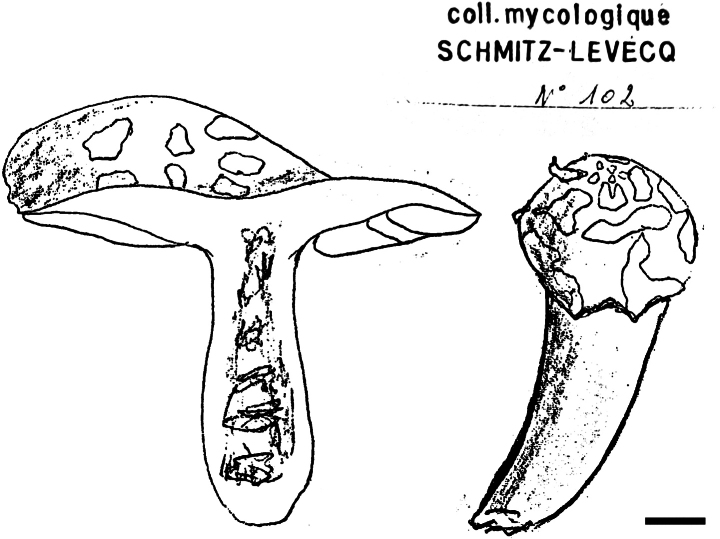
*Russularoseovelata*. Pencil drawing of the holotype collection of *Russularoseovelata* by Schmitz-Levecq preserved in BR. Scale bar: 1 cm.

**Figure 8.**
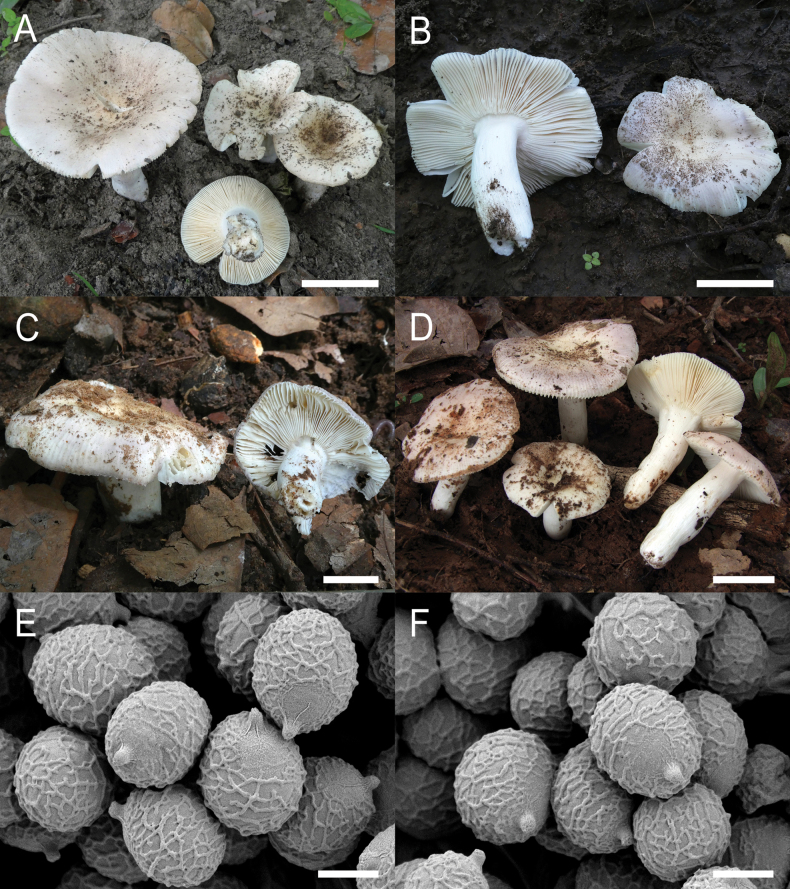
*Russulabeenkenii***A–D** field pictures of basidiomata: **A** CM-21-047, holotype, **B** CM-21-132, **C** CM-21-152, **D** CM-22-232; **E, F** scanning electron microscopical pictures of basidiospores (both CM-21-047, holotype). Scale bars: 3 cm (**A, D**); 2 cm (**B**); 1 cm (**C**); 3 µm (**E, F**).

**Figure 9.**
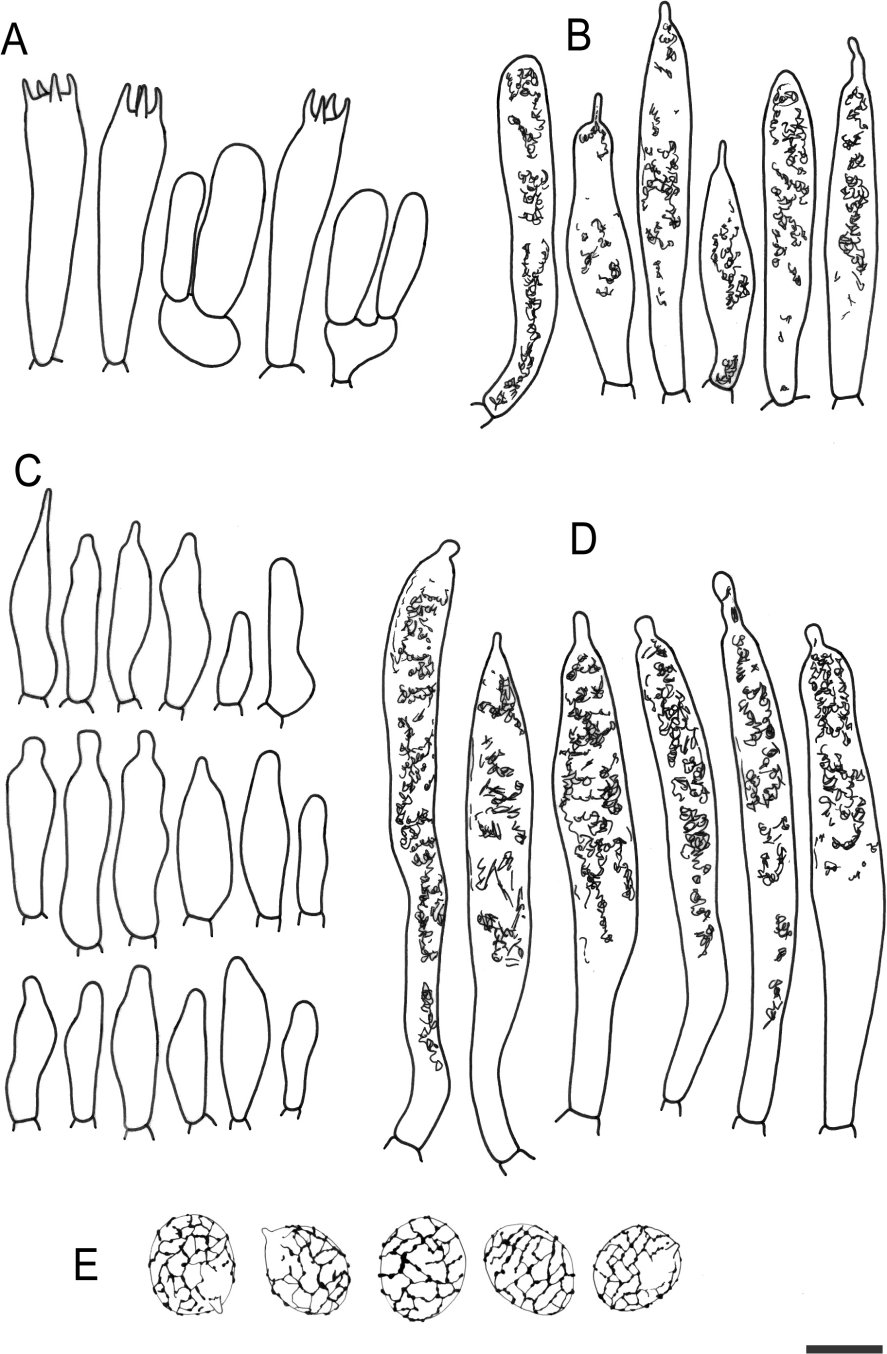
Hymenial elements of *Russulabeenkenii*. (holotype, CM-21-047) **A** basidia and basidiola, **B** hymenial cystidia near the lamellar edges, **C** marginal cells, **D** hymenial cystidia, **E** spores as seen in Melzer’s reagent. Scale bar: 10 μm, but only 5 μm for spores.

**Figure 10.**
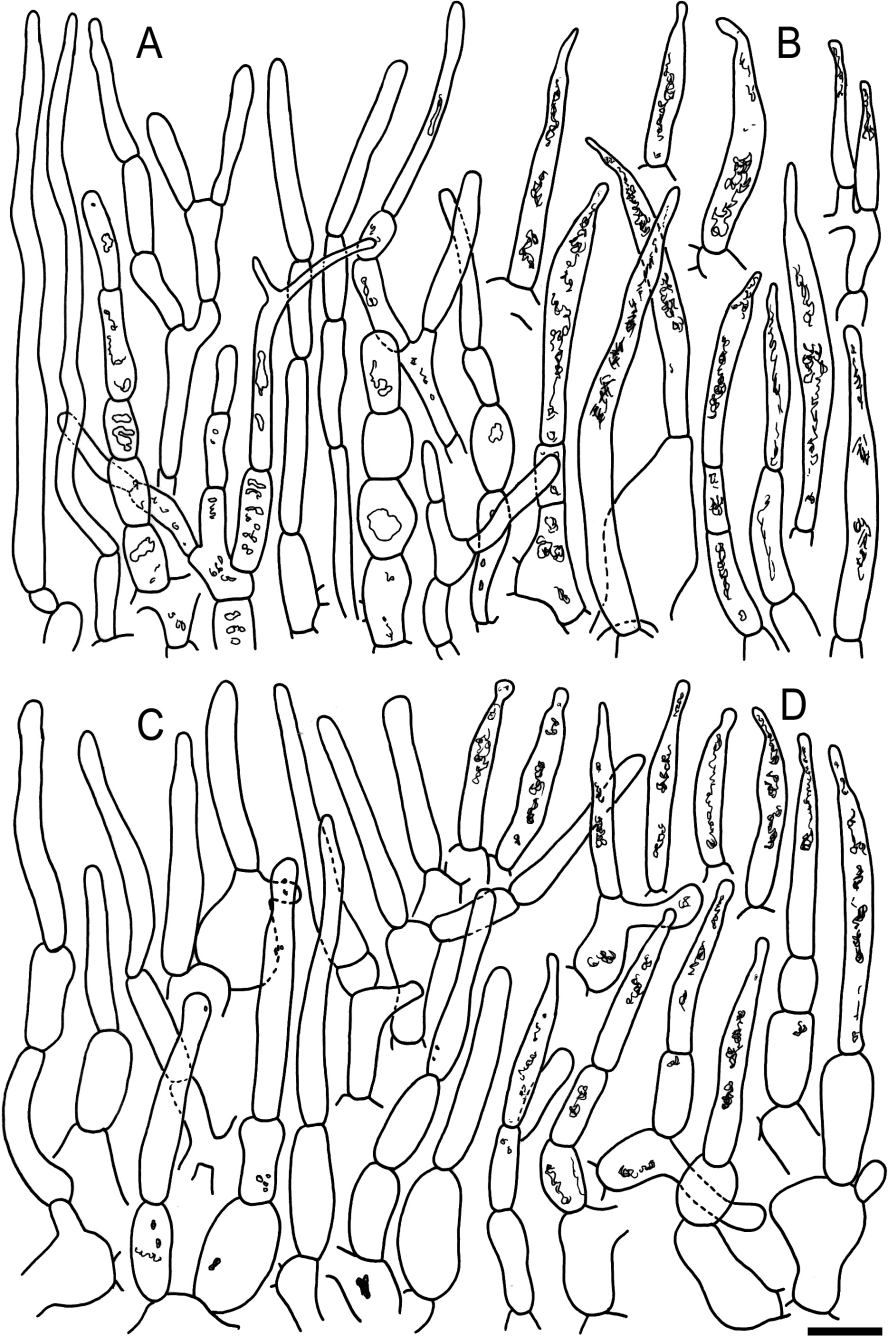
Elements of the pileipellis of *Russulabeenkenii*. (holotype, CM-21-047) **A, B** elements near the pileus centre: **A** hyphal terminations, **B** pileocystidia; **C, D** elements near the pileus margin: **C** hyphal terminations, **D** pileocystidia. Scale bar: 10 μm.

#### ﻿ Russula carmesina

Taxon classificationAnimaliaRussulalesRussulaceae

Heim, Rev. Mycol. (Paris) 34 (4): 347 (1970)

08D94646-44E9-54C0-91C0-DB47E15B08C6

322906

**Figure 11.**
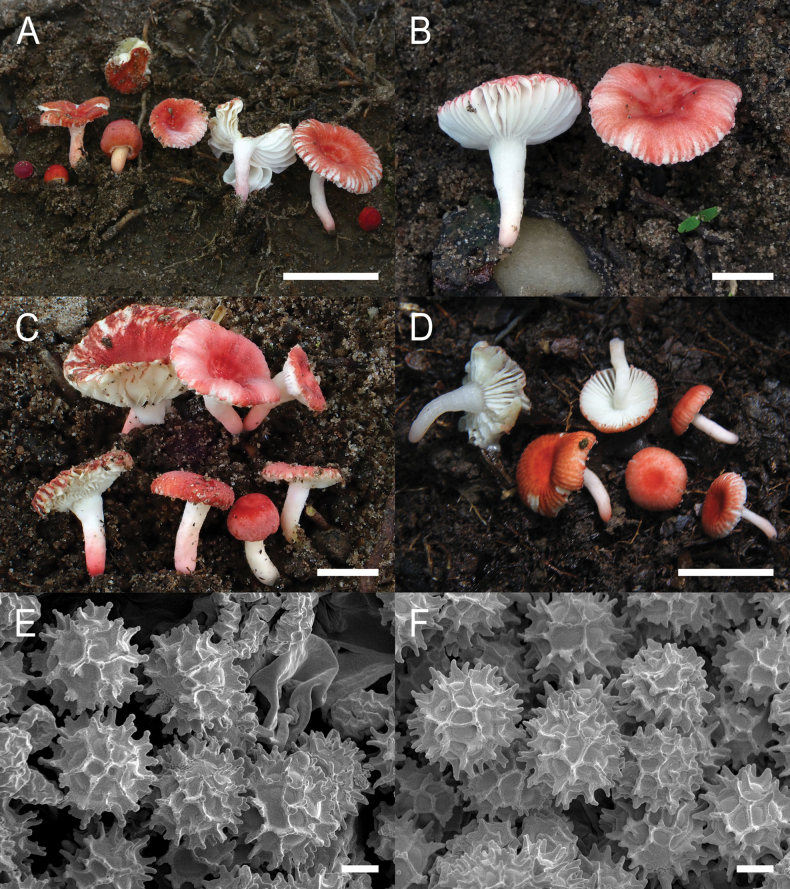
*Russulacarmesina***A–D** field pictures of basidiomata: **A** CM-21-143, **B** CM-21-108, **C** CM-22-282, **D** SYN5103; **E, F** scanning electron microscopical pictures of basidiospores (both CM-21-143). Scale bars: 1 cm (**A, D**); 0.5 cm (**B, C**); 3 µm (**E, F**).

**Figure 12.**
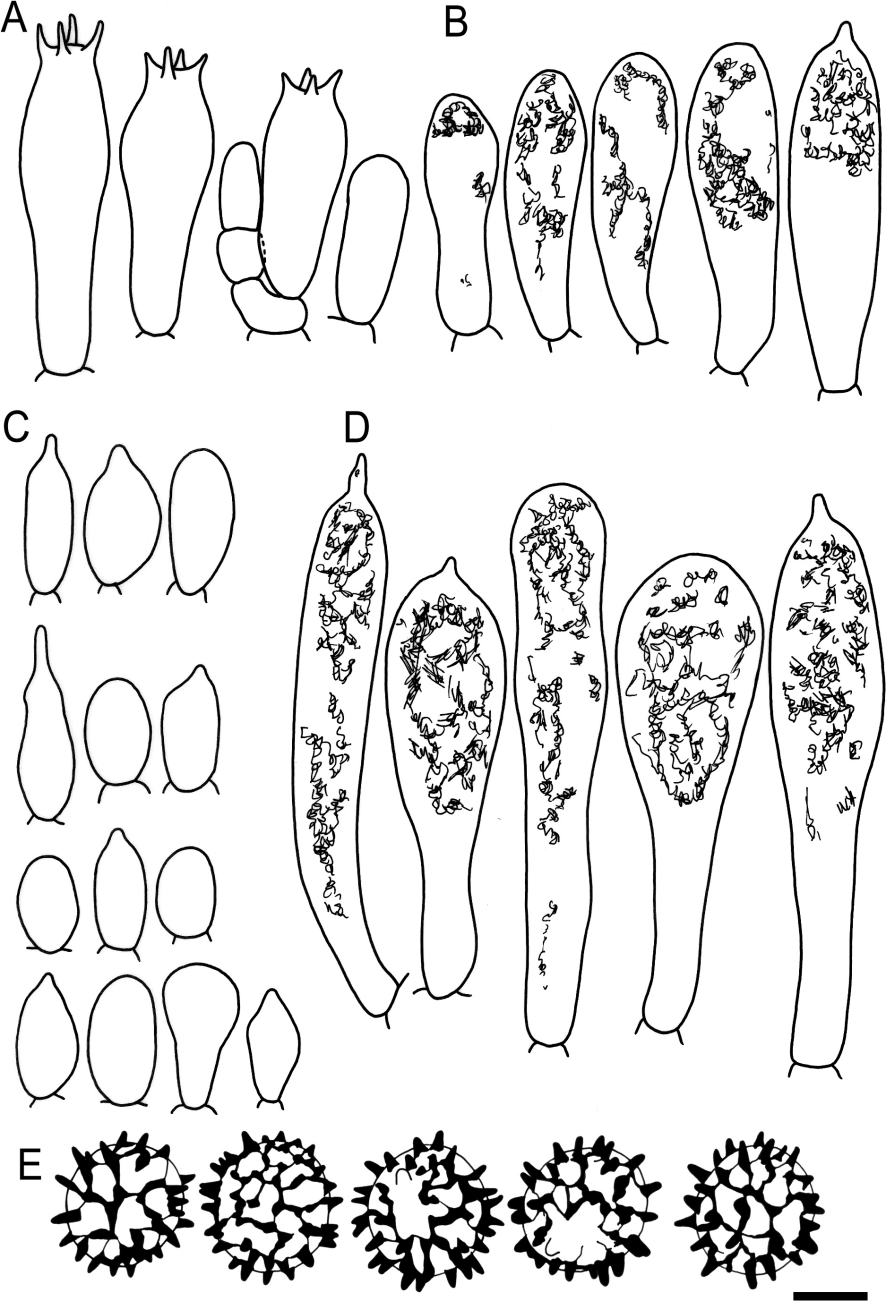
Hymenial elements of *Russulacarmesina* (CM-21-143) **A** basidia and basidiola, **B** hymenial cystidia near the lamellar edges, **C** marginal cells, **D** hymenial cystidia, **E** spores as seen in Melzer’s reagent. Scale bar: 10 μm, but only 5 μm for spores.

**Figure 13.**
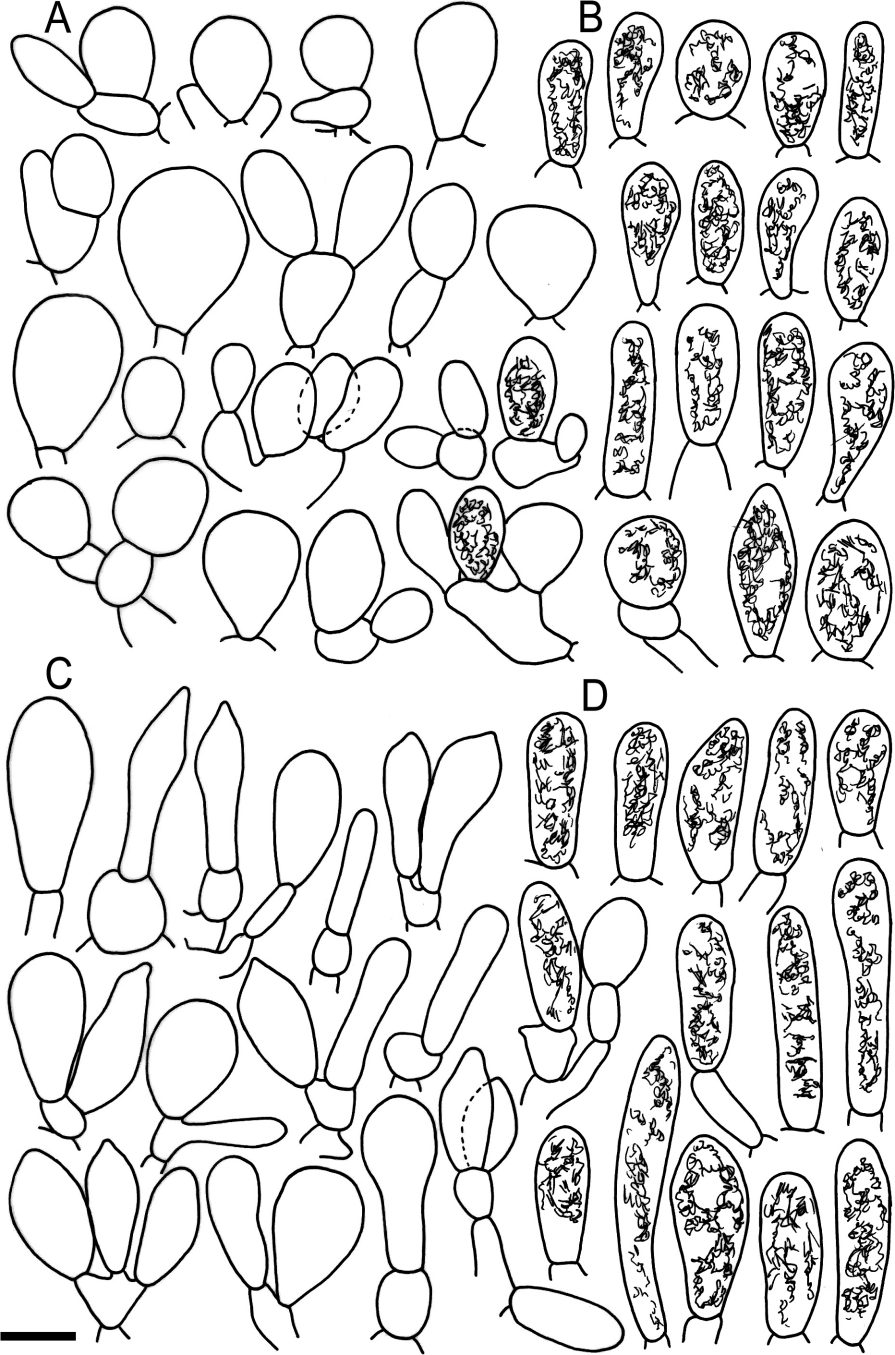
Elements of the pileipellis of *Russulacarmesina* (CM-21-143) **A, B** elements near the pileus centre: **A** hyphal terminations, **B** pileocystidia; **C, D** elements near the pileus margin: **C** hyphal terminations, **D** pileocystidia. Scale bar: 10 μm.

**Figure 14.**
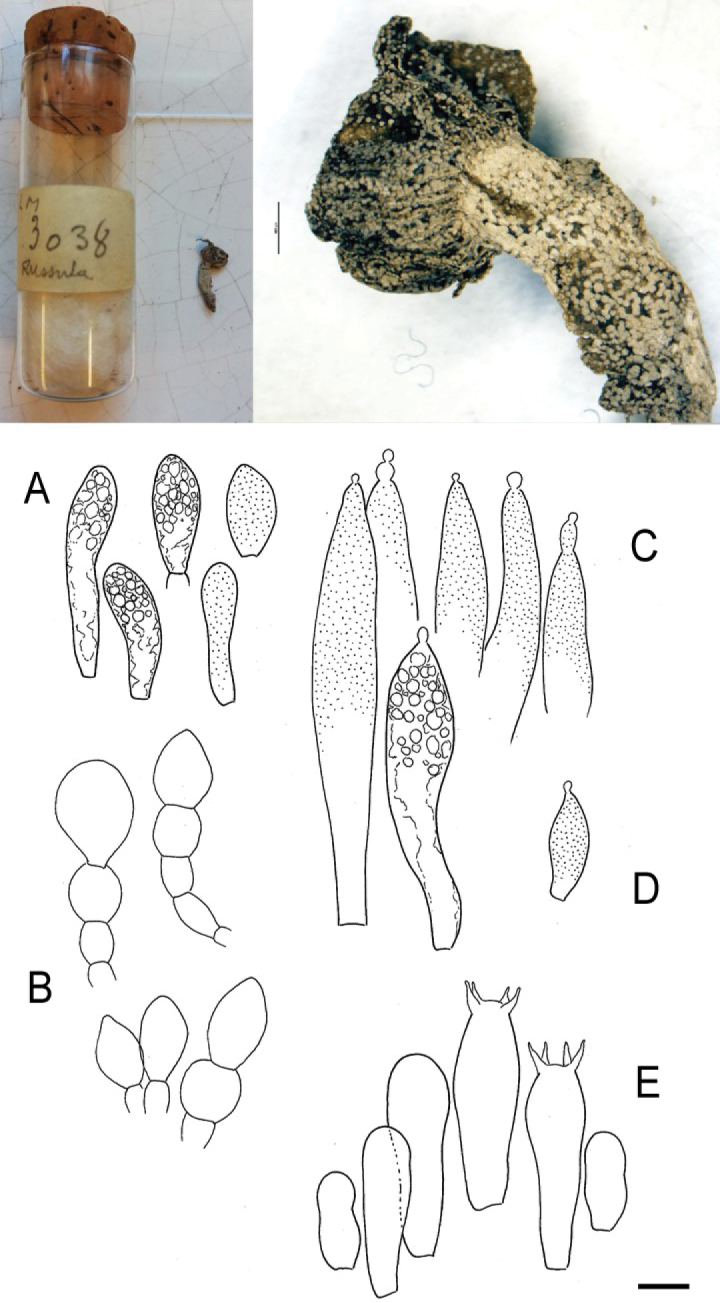
Holotype material of *Russulacarmesina* (LM 3038). **Top** photographs of the holotype material preserved in PC; **Bottom** micromorphology. **A** pileocystidia, **B** hyphal terminations of the pileipellis, **C** hymenial cystidia, **D** hymenial cystidium near the lamellar edge, **E** basidia and basidiola. Scale bar: 10 μm.

Figs 11 , 12 , 13 , 14

##### Holotype.

Central African Republic. gallery forest along the Lobaye River, solitary, directly on the ground, 12.05.1968, leg. R. Heim, *LM 3038* (PC0798351).

##### Additional material examined.

Benin. Atakora, Natitingou, Kota waterfalls, co-ord. 10°12.7'N, 1°26.8'E, alt. 500 m, in a gallery forest, under *Uapacaguineensis*, directly along the riverside on bare sand, 11.07.2021, leg. C. Manz, F. Hampe, N. S. Yorou & G. Abohoumbo, *CM-21-108* (B 70 0105417, UNIPAR); *ibid.* 19.07.2021, leg. C. Manz, F. Hampe, N. S. Yorou, G. Abohoumbo & D. Dongnima, *CM-21-143* (B 70 0105418, UNIPAR); *ibid.* 05.07.2022, leg. C. Manz & F. Hampe, *CM-22-282* (B 70 0105419, UNIPAR); Guinea. Kankan, National Park of Upper Niger, co-ord. 10°14.8'N, 10°27.8'W, under *Isoberliniadoka*, *Anthonothafragrans* (Baker f.) Exell & Hillc., *Anthonothacrassifolia* (Baill.) J. Léonard & *Uapacatogoensis*, 07.08.2022, leg. N. S. Yorou, *SYN5103* (UNIPAR).

##### Short description.

Very small basidiomata, pileus surface bright red, lamellae edges partly reddish, stipe narrowing towards the frequently reddish base, pileipellis a hymeniderm, occurring in gallery forests.

##### Description based on material recently collected in Benin and Guinea.

**Growth habit**: basidiomata solitary or in small groups of approx. ten. **Pileus**: very small to small, 3–12 mm in diam., when young, hemispherical, later convex, expanding plane, centrally depressed; margin even, frequently finely radially cracked up to 3 mm, when young with a very fugitive, pale, veil-like overhanging membrane that is not reaching the stipe, when mature, visible as very fine scales, giving a slightly uneven appearance, never forming an annulus; cuticle smooth, matt, finely radially fibrous, somewhat velvety, peelable up to ½ of the pileus radius, partly descending on to the lamellae edges, colour when young, blood red (10C8), later cherry red (10B8), lake red (9C8) or lobster red (9B8), sometimes paler towards the margin: red (9A6) or pastel red (9A5). **Lamellae**: 1–2 mm wide, 10–11 lamellae present along 1 cm near the pileus margin, ventricose, adnexed, sometimes with a decurrent tooth, white, furcations, anastomoses and lamellulae absent, edges entire, white or frequently red near the pileus margin. **Stipe**: 5–15 × 2–3 mm, cylindrical or frequently narrowing towards the base; smooth to slightly rugose, annulus absent, white, covered partly also with a pastel red (9A4) hue at the base or the entire stipe; cottony stuffed. **Context**: only up to 0.5 mm thick at half pileus radius, white, unchanging when bruised, very fragile, taste mild, odour inconspicuous; macrochemical reactions: guaiac after 8–10 seconds negative (-) on both stipe and lamellae surfaces, sulphovanillin negative, FeSO_4_, KOH and phenol not tested. **Spore print**: white (Ia).

**Spores**: (6.9–)7.9–8.4–8.8(–9) × (6.6–)7.5–7.9–8.4(–9) µm (n = 61), Q = (1–)1.02–1.05–1.09(–1.15), globose to subglobose; ornamentation of distant to moderately distant amyloid spines [2–4(–6) in a circle of 3 µm diam.], 1–2 µm high, connected by numerous lines [2–4(–5) in the circle] forming a complete reticulum, spines occasionally fused in pairs (0–2 fusions in the circle), tips frequently bifurcate as seen by SEM; suprahilar plage moderately large, inamyloid, without ornamentation. **Basidia**: (28–)33.5–37.5–41.5(–46) × (10–)11–12–13(–14.5) µm (n = 40), clavate to broadly clavate, 4-spored; basidiola approx. 5–8 µm wide, cylindrical or clavate. **Hymenial cystidia**: on lamellae sides (51–)60–71–82(–88) × (8–)10–13–15(–20) µm (n = 40), widely dispersed, 60–130 /mm^2^, narrowly to broadly clavate, rarely subcylindrical, originating in subhymenium and somewhat protruding over basidia, thin-walled, apically obtuse, with or without one or two 1.5–15 µm long appendages; heteromorphous contents abundant, crystalline to amorphous, mostly located in the upper half, turning distinctly greyish-black in sulphovanillin after 2–3 minutes. Hymenial cystidia near the lamellae edges distinctly shorter, (28.5–)32–37.5–43.5(–53.5) × (9–)10.5–11.5–12.5(–14) µm (n = 40), clavate to broadly clavate, rarely slightly constricted, occasionally with a 1.5–4 µm long appendage; heteromorphous contents similar to the one in hymenial cystidia on lamellae sides. **Lamellae edges**: fertile, with equal representation of cystidia, basidia, basidiola and marginal cells. **Marginal cells**: (11–)13–16.5–19.5(–26) × (5–)6–7.5–8.5(–10.5) µm (n = 40), predominantly fusiform with acute apex, sometimes ovoid with obtuse apex, optically empty, thin-walled. **Pileipellis**: orthochromatic in Cresyl blue, gradually passing to the underlying context, not gelatinised, 50–70 µm deep; suprapellis a hymeniderm or epithelium, 20–27 µm deep, composed of one or two layers of inflated terminal cells; sharply delimited from a 20–37 µm deep subpellis of moderately dense, horizontally orientated, 2.5–4 µm wide interwoven hyphae. Acid resistant encrustations absent. **Hyphal terminations**: near the pileus margin usually composed of single or sometimes two cells before branching, thin-walled; terminal cells (11.5–)14.5–18–21.5(–26.5) × (3.5–)7–9.5–12(–14.5) µm (n = 60), predominantly ovoid or clavate to broadly clavate with obtuse apex, frequently also fusiform with pointed apex, rarely cylindrical; subterminal cells distinctly shorter, more or less isodiametric, 3–10 µm wide, mainly branched. Hyphal terminations near the pileus centre similar to the ones near the pileus margin, terminal cells (7–)11–14–17(–21.5) × (5–)7–9.5–12(–17) µm (n = 60), globose to subglobose or ovoid with obtuse apex; subterminal cells almost exclusively branched. **Pileocystidia**: near the pileus margin (16.5–)17–22–27(–40.5) × (6–)7–8.5–9.5(–11.5) µm (n = 40), one-celled, cylindrical to broadly clavate, rarely base-inflated, originating in the suprapellis, thin-walled, apically obtuse, without appendages; heteromorphous contents dense, crystalline to amorphous, present in the entire cell, turning distinctly greyish-black in sulphovanillin after 2–3 minutes. Pileocystidia near the pileus centre distinctly shorter, (12–)15–19–23(–28.5) × (4–)6.5–8–9.5(–11.5) µm (n = 40), globose to ovoid, clavate or broadly cylindrical; contents similar to the one in pileocystidia near the pileus margin. **Context**: very thin in pileus, consisting of a layer only 1–2 sphaerocysts thick; cystidioid and oleiferous hyphae absent.

##### Distribution and ecology.

Known from gallery forests in Guinea, Benin and Central African Republic.

##### Notes.

In the past, *Russulacarmesina* was placed in subsect.Pseudoepitheliosinae together with several other species with small basidiomata and a hymenidermal pileipellis structure, predominantly distributed in Africa (Buyck 1990b). It is very similar to *Russulaparasitica* (R. Heim) Buyck and *Russulapseudocarmesina* Buyck; however, these two species are distinctly annulate (Buyck 1994). In addition, *R.pseudocarmesina* has larger basidiomata with pileus diameters of 2–4 cm (Buyck 1994). *Russulapseudoepitheliosa* Buyck is a similar species without annulus, but also has larger basidiomata with purplish-violet pileus colours (Buyck 1994). In the original description of *R.carmesina*, Heim did not mention the presence of pileocystidia, although he was usually mentioning these elements in his other *Russula* descriptions when he observed them (Heim 1970). As the holotype material was lost for decades, *R.carmesina* was thought to be lacking pileocystidia and the placement in subsect.Pseudoepitheliosinae was uncertain (Buyck 1990b). Fortunately, we were able to locate the type material. It was in a bad condition, since it was previously stored in ethanol which evaporated a long time ago, leaving a miniscule, blackish remnant of a basidioma encrusted by crystalline matter. However, by microscopic investigation, the presence of obvious pileocystidia in the material could be revealed (Fig. 14).

#### ﻿ Russula coronata

Taxon classificationAnimaliaRussulalesRussulaceae

Manz & F. Hampe, sp. nov.

85115E41-2F4D-510F-808A-0366766FF7CB

856865

Figs 15 , 16 , 17

##### Diagnosis.

Pileus surface violet-grey with fugitive jagged white remnants of a partial veil at the margin, taste mild, spore print white, marginal cells near lamella edges with finger-like projections and pileocystidia staining slightly violet-grey in sulphovanillin, occurring in gallery forests. Differs from *Russulaannulata*R. Heim by white-coloured lamellae edges.

##### Holotype.

Benin. Donga, Bassila, co-ord. 9°0.1'N, 1°38.9'E, alt. 360 m, in a gallery forest, under *Berliniagrandiflora*, on sandy soil, 30.06.2022, leg. C. Manz, F. Hampe, S. Sarawi, A. Rühl & R. Dramani, *CM-22-241* (**holotype** B 70 0105420, **isotype**UNIPAR).

##### Additional material examined.

Benin. Donga, Bassila, co-ord. 9°0.1'N, 1°38.9'E, alt. 360 m, in a gallery forest, under *B.grandiflora* on sandy soil, 02.07.2022, leg. C. Manz, F. Hampe, S. Sarawi, A. Rühl & R. Dramani, *CM-22-261* (**paratype**, B 70 0105421, UNIPAR).

##### Description.

**Growth habit**: basidiomata solitary or in groups of two. **Pileus**: medium-sized, 60–65 mm in diam., plane, slightly centrally depressed; margin even, distinctly striate up to ^1^/_3_ to ½ of the pileus radius, regularly shaped, sometimes slightly undulate, finely crenulate, when young, covered by a crown of fugitive, jagged, white partial veil remnants; cuticle smooth, matt, finely pruinose, not peelable, colour near the margin purplish-grey (13B2) or greyish-magenta (13B3, 14D3–4, 14E3–4), near the centre greyish-magenta (13C3) or dark purple (14F3–4), sometimes with light milk-white (1B2) spots. **Lamellae**: 6–7 mm wide, 5–6 lamellae present along 1 cm near the pileus margin, narrowly adnate or emarginate, white, occasionally furcated near the stipe attachment, anastomoses and lamellulae absent; edges entire, concolourous. **Stipe**: 55–60 × 11–13 mm, cylindrical, sometimes narrowing towards the base, slightly bulging here and there; smooth to slightly rugose, annulus absent, white with a greyish-magenta (14E3) hue near the base; hollow to slightly cottony stuffed. **Context**: approx. 0.5 mm thick at half pileus radius, white, unchanging when bruised, brittle, taste mild, odour inconspicuous; macrochemical reactions: guaiac after 8–10 seconds negative on stipe and positive (++) on lamellae surfaces, FeSO_4_ weak salmon orange, sulphovanillin negative, KOH negative, phenol negative. **Spore print**: white (Ia).

**Spores**: (7.3–)7.9–8.4–8.8(–9.4) × (6.9–)7.6–8.1–8.5(–9.2) µm (n = 60), Q = (1–)1.01–1.04–1.06(–1.09), globose to subglobose; ornamentation of large, distant, amyloid spines [(1–)2–3((–4) in a circle of 3 µm diam.], 1.7–2.6 µm high, connected by numerous lines [(1–)2–4(–5) in the circle] forming a complete reticulum, isolated elements absent, spines and line connections with frequent secondary warts visible only by SEM; suprahilar plage small, inamyloid, without ornamentation. **Basidia**: (34–)37.5–43.5–49.5(–59) × (12.5–)14–15–16(–17) µm (n = 40), clavate to broadly clavate, 4-spored; basidiola approx. 8–9 µm wide, clavate to subclavate. **Hymenial cystidia**: on lamellae sides (86–)93.5–99.5–106(–112) × (10–)12–14.5–16.5(–22) µm (n = 40), fusiform, originating in subhymenium and somewhat protruding over basidia, thin-walled, apically obtuse, with a 2–15 µm long appendage; heteromorphous contents amorphous, located in the upper half, not reacting to sulphovanillin. Hymenial cystidia near the lamellae edges shorter and narrower, (43–)54.5–65–75.5(–89.5) × (8–)9–11–12.5(–14) µm (n = 40), cylindrical or fusiform, sometimes slightly centrally constricted, with one or two 1–8 µm long terminal knobs; heteromorphous contents less dense, sometimes located only in the upper half. **Lamellae edges**: fertile, marginal cells intermixed with basidia and basidiola. **Marginal cells**: (19–)23–27–31.5(–36) × (4–)5–6.5–8.5(–10.5) (n = 40), variably shaped, coarsely diverticulate with several finger-like, irregular projections, optically empty, thin-walled. **Pileipellis**: orthochromatic in Cresyl blue, gradually passing to the underlying context, 170–300 µm deep; suprapellis a trichoderm, 25–45 µm deep, composed of erect hyphal terminations embedded in a gelatinous matrix; gradually passing to a 170–270 µm deep subpellis of loosely interwoven, irregularly orientated, strongly gelatinised, 2.5–3 µm wide hyphae, becoming denser and horizontally arranged near the context. Acid resistant incrustations absent. **Hyphal terminations**: near the pileus margin composed of 1–3 unbranched cells, thin-walled, terminal cells (13–)23.5–29.5–35.5(–44) × (3–)3.5–5–6(–7) µm (n = 60), predominantly subulate, rarely cylindrical, apically obtuse; subterminal cells distinctly shorter, 4–9 µm wide, ellipsoid or cylindrical. Hyphal terminations near the pileus centre of 2–4(–5) unbranched cells, thin-walled; terminal cells (9–)13–19.5–25.5(–36) × (2.5–)3.5–4–4.5(–6.5) µm (n = 60), subulate or cylindrical; subterminal cells 3–6 µm wide, mostly cylindrical, becoming barrel-shaped close to the subpellis. **Pileocystidia**: near the pileus margin (19.5–)23–30–37(–49.5) × (4–)4.5–5.5–6.5(–7) µm (n = 40), one-celled, usually lanceolate, sometimes subcylindrical, originating in the suprapellis or in upper part of the subpellis, thin-walled, apically obtuse, with a 4–7 µm long appendage or terminal knob; heteromorphous contents amorphous, turning slightly to violet-grey in sulphovanillin. Pileocystidia near the pileus centre similar in size, shape and heteromorphous contents to pileocystidia near the pileus margin, (18–)21.5–27–32.5(–44) × 3.5–4.5–5.5(–7) µm (n = 40). **Context**: without cystidioid hyphae, oleiferous hyphae frequent.

##### Etymology.

corona = crown, referring to the jagged velar patches on the pileus margin.

##### Distribution and ecology.

Only known from the Bassila gallery forest in Benin.

##### Notes.

Both collections of *R.coronata* showed serrated fragments of a partial veil on the pileus margin, for which we introduce the term “crown”. The presence of a partial veil is frequent in tropical African *Russula* species. In several species, the veil is very fugacious and remnants can be observed either as a ring attached to the stipe or the pileus margin or as a crown on the pileus margin or lost due to weather impacts. This feature should, therefore, be treated with caution when identifying *R.coronata*. *Russulaannulata* is a violet-grey capped species described from Madagascar with an annulus or crown (Heim 1937). It shares the reticulate spore ornamentation and the trichodermal pileipellis with shorter ellipsoid to subglobose subterminal cells, but differs by a stipe entirely covered by vivid violet pustules, lamellae with a dark violet edge and violet-coloured context under the pileipellis (Heim 1937, 1938). The type specimen of *R.annulata* is lost (Buyck 1994) and, after reviewing the original description, we assume that it is based on a mixture of similar species due to the considerable morphological variations described by Heim. Therefore, the macromorphological comparison is based on the original aquarelle painting of the type specimen (Heim 1937). *Russulaannulatosquamosa* Beeli and *Russulaannulatolutea* Beeli are other annulate species which differ by olive yellow pileus colours and acrid taste (Beeli 1936). *Russulaacriannulata* Buyck is an annulate species with ochre-brown pileus colours which shares the trichodermal pileipellis structure with single-celled pileocystidia, but differs by spores ornamented by isolated warts (Härkönen et al. 1993). *Russulaannulatobadia* Beeli is an annulate species with wine-red pileus colours which shares the reticulate spore ornamentation, but differs by the absence of pileocystidia and the presence of filiform marginal cells (Buyck 1994). *Russulaacuminata* Buyck is a species without remnants of a partial veil and with wine-red pileus colours which shares similar marginal cells and spores, but differs by strongly ramified hyphal terminations in the pileipellis (Buyck 1994).

**Figure 15.**
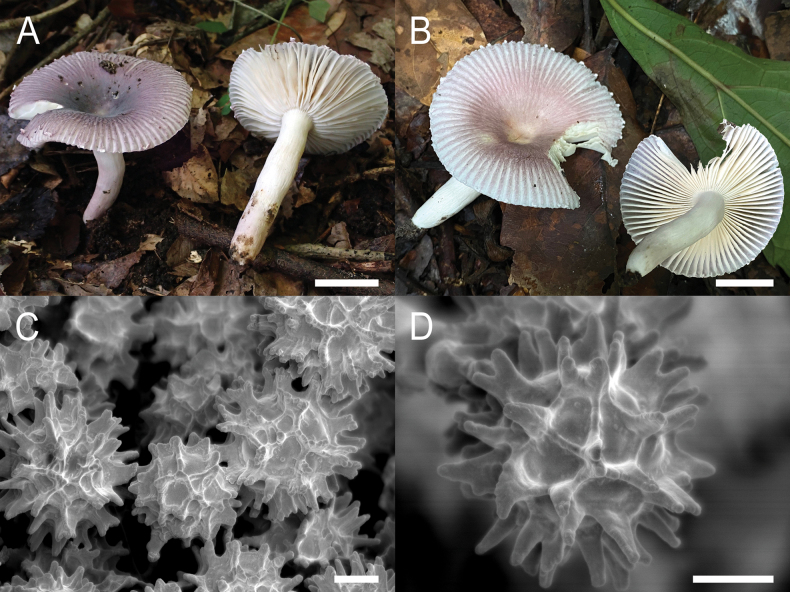
*Russulacoronata***A, B** field pictures of basidiomata: **A** CM-22-241, holotype, **B** CM-22-261; **C, D** scanning electron microscopical pictures of basidiospores (both CM-22-241, holotype). Scale bars: 2 cm (**A, B**); 3 µm (**C, D**).

**Figure 16.**
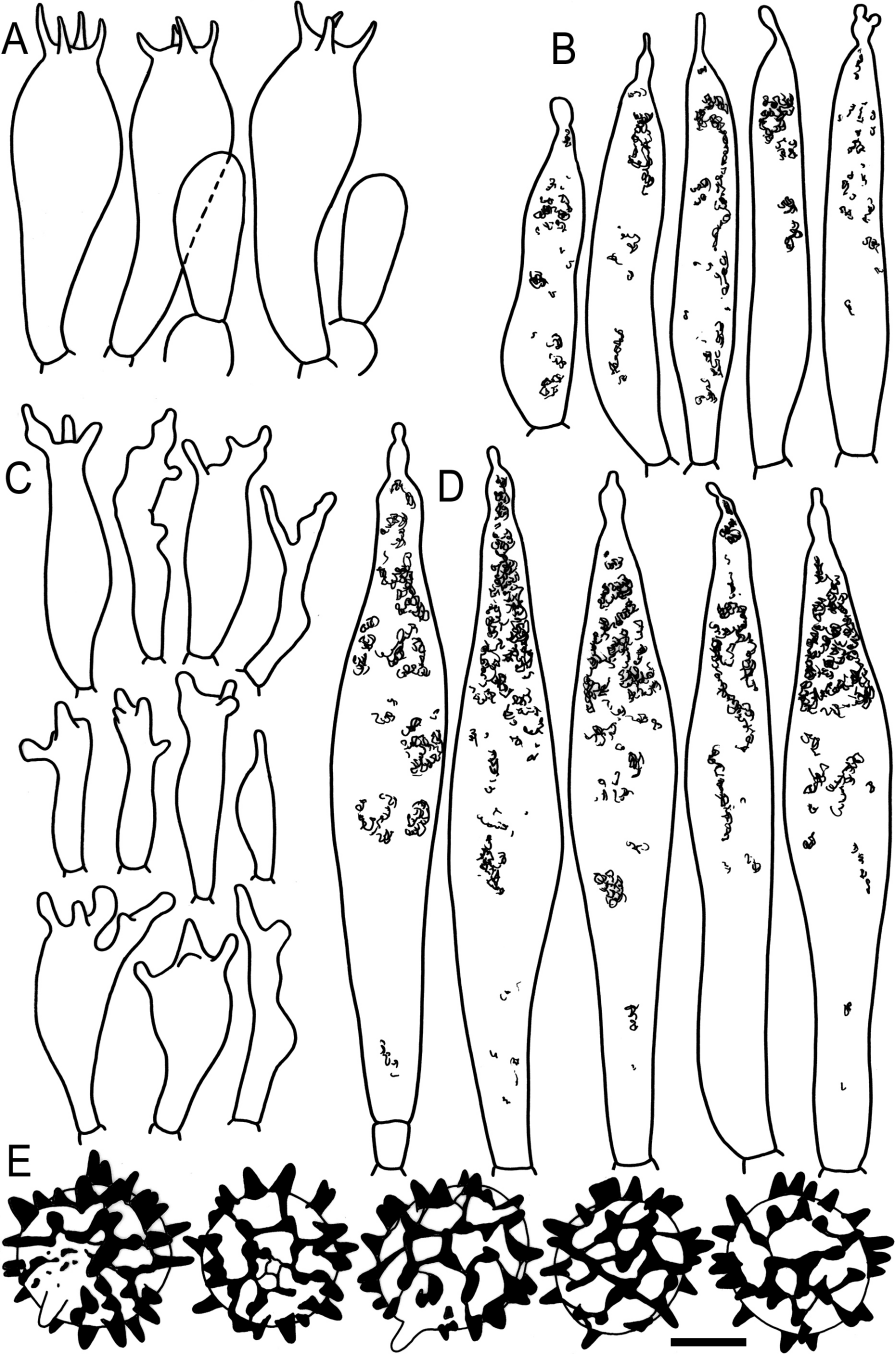
Hymenial elements of *Russulacoronata* (holotype, CM-22-241) **A** basidia and basidiola, **B** hymenial cystidia near the lamellar edges, **C** marginal cells, **D** hymenial cystidia, **E** spores as seen in Melzer’s reagent. Scale bar: 10 μm, but only 5 μm for spores.

**Figure 17.**
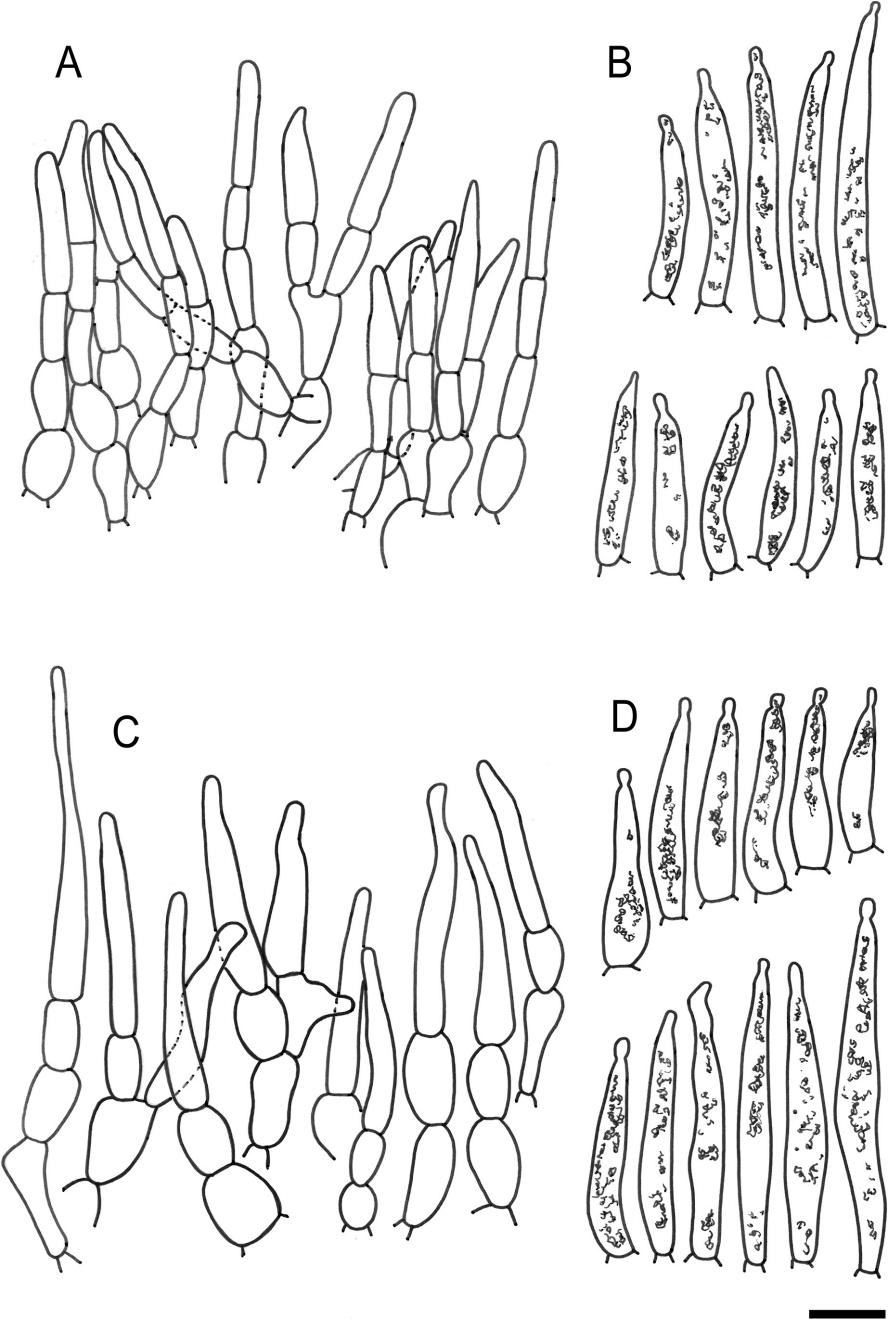
Elements of the pileipellis of *Russulacoronata* sp. (holotype, CM-22-241) **A, B** elements near the pileus centre: **A** hyphal terminations, **B** pileocystidia; **C, D** elements near the pileus margin: **C** hyphal terminations, **D** pileocystidia. Scale bar: 10 μm.

#### ﻿ Russula florae

Taxon classificationAnimaliaRussulalesRussulaceae

Manz & F. Hampe, sp. nov.

EAA8EEFA-1664-59E2-9C0E-442C4A589617

856866

Figs 18 , 19 , 20

##### Holotype.

Benin. Atakora, Natitingou, Kota Waterfalls, co-ord. 10°12.7'N, 1°26.8'E, alt. 500 m, in a gallery forest, under *Uapacaguineensis* on rocky soil, 10.07.2021, leg. C. Manz, F. Hampe, *CM-21-098* (**holotype** B 70 0105422, **isotype**UNIPAR).

##### Additional material examined.

Benin. Atakora, Natitingou, Kota Waterfalls, co-ord. 10°12.8'N, 1°26.8'E, alt. 500 m, in a gallery forest, under *U.guineensis* & *Berliniagrandiflora*, directly along the riverside on bare sand, 11.07.2021, leg. C. Manz, F. Hampe, N. S. Yorou, G. Abohoumbo & D. Dongnima, *CM-21-109* (**paratype**, B 70 0105423, UNIPAR); *ibid.* 14.07.2021, leg. C. Manz, F. Hampe, N. S. Yorou, G. Abohoumbo & D. Dongnima, *CM-21-121* (**paratype**, B 70 0105424, UNIPAR); *ibid.* 19.07.2021, leg. C. Manz, F. Hampe, N. S. Yorou, G. Abohoumbo & D. Dongnima, *CM-21-145* (**paratype**, B 70 0105425, UNIPAR); *ibid.* leg. N. S. Yorou, *CM-21-160/SYN5074* (**paratype**, B 70 0105426, UNIPAR); *ibid.* co-ord. 10°12.7'N, 1°26.6'E, alt. 500 m, in a gallery forest, under *U.guineensis* & *B.grandiflora*, directly along the riverside in between fine gravel, 15.06.2022, leg. C. Manz, F. Hampe, S. Sarawi, A. Rühl & D. Dongnima, *CM-22-178* (**paratype**, B 70 0105427, UNIPAR); *ibid.* 17.06.2022, leg. C. Manz, F. Hampe, S. Sarawi, A. Rühl & D. Dongnima, *CM-22-185* (**paratype**, B 70 0105428, UNIPAR); *ibid.* 06.07.2022, leg. C. Manz & F. Hampe, *CM-22-284* (**paratype**, B 70 0105429, UNIPAR).

##### Diagnosis.

Relatively small, pileus surface pinkish and cracking into fine areolae, stipe without annulus, context fragile, taste mild, hyphal terminations in pileipellis arranged in erect tufts, occurring in gallery forests. Differs from *R.roseoalba* by its negative reaction to guaiac.

##### Description.

**Growth habit**: basidiomata solitary or in small groups of up to five. **Pileus**: small to medium-sized, 10–40 mm in diam., when young hemispherical, truncated, with margins touching the stipe, later expanding plane, when mature, centrally depressed; margin even or slightly involute, distinctly tuberculate-striate up to ¾ of the pileus margin, frequently slightly to distinctly radially cracked up to ½ of the pileus radius, mostly crenulate, sometimes undulate, usually with crown of fugitive partial veil remnants; cuticle matt, very finely areolate, hardly peelable, colour near the margin white, pinkish-white (10-11A2) or reddish-grey (10B2), becoming gradually darker towards the centre, rosewood (9D5), dull red (10B3), brownish-red (10C6), reddish-grey (11B2) or violet brown (10E5), occasionally with greyish-green (30B4) spots. **Lamellae**: 2–3 mm wide, 6–7 lamellae present along 1 cm near the pileus margin, narrowly adnate, white, occasionally forked, low anastomoses only at the pileus margin, lamellulae absent; edges entire, concolourous. **Stipe**: 25–35 × 3–5 mm, cylindrical, somewhat bulging here and there, smooth to slightly rugose, annulus absent, white; hollow. **Context**: approx. 1 mm thick at half pileus radius, white, unchanging when bruised, brittle, taste mild, odour inconspicuous; macrochemical reactions: guaiac after 8–10 seconds negative on both stipe and lamellae surfaces, FeSO_4_ salmon orange, sulphovanillin, KOH and phenol negative. **Spore print**: not observed, but probably white or cream.

**Spores**: (6.8–)7.2–7.6–8(–8.8) × (6.3–)6.8–7.2–7.6(–8.7) µm (n = 90), Q = (1–)1.02–1.06–1.09(–1.17), subglobose, rarely broadly ellipsoid; ornamentation of distant to moderately distant amyloid warts [(2–)3–5(–6) in a circle of 3 µm diam.], 1–1.8 µm high, connected by frequent to abundant lines [(1–)2–4(–5) in the circle] forming a complete reticulum, rarely fused (up to 1 fusion in the circle), warts frequently rimmed by additional smaller warts visible only by SEM; suprahilar plage small, inamyloid, without ornamentation. **Basidia**: (32–)35–39–43(–48) × (10–)11–12.5–14.5(–20) µm (n = 60), clavate to subclavate, 4-spored; basidiola approx. 5–10 µm wide, cylindrical to clavate. **Hymenial cystidia**: on lamellae sides (61–)70.5–81–91.5(–114) × (7–)9.5–12–14.5(–20) µm (n = 61), predominantly fusiform, rarely subclavate, originating in subhymenium and somewhat protruding over basidia, thin-walled, usually with a 3–15 µm long appendage, rarely with 2 appendages; heteromorphous contents amorphous, mostly located in the upper third, not reacting to sulphovanillin. Hymenial cystidia near the lamellae edges distinctly shorter, (37.5–)44–51.5–59(–70.5) × (8–)10–11.5–13(–15) µm (n = 60), similar in shape to hymenial cystidia on lamellae sides; heteromorphous contents only located near the apex, distinctly less dense. **Lamellae edges**: fertile, with equal representation of cystidia, basidia, basidiola and marginal cells. **Marginal cells**: (10–)14.5–19.5–24(32.5) × (4–)5.5–7–8.5(–11) µm (n = 60), not well differentiated, variable in shape, cylindrical, clavate, pyriform, utriform or fusiform, sometimes hard to distinguish from basidiola, optically empty, thin-walled. **Pileipellis**: orthochromatic in Cresyl blue, sharply delimited from the underlying context, 145–185 µm deep; suprapellis a trichoderm, 35–50 µm deep, not gelatinised, composed of erect hyphal terminations; well delimited from the 100–145 µm deep subpellis of loose, irregularly orientated, interwoven, strongly gelatinised, 4–9 µm wide hyphae, becoming denser and horizontally arranged near the context. Acid resistant encrustations absent. **Hyphal terminations**: near the pileus margin arranged in erect tufts corresponding to the fine, macroscopically visible areolae, composed of (1–)3–4 unbranched cells, thin-walled, terminal cells (15.5–)24.5–30–36(–44) × (3–)4–5–5.5(–6.5) µm (n = 90), subulate, rarely subcylindrical, apically obtuse; subterminal cells shorter, 3.5–9 µm wide, cylindrical or ellipsoid. Hyphal terminations near the pileus centre similar to the ones near the pileus margin, terminal cells shorter, (9.5–)15–22–29(–39) × (3–)4–4.5–5.5(–6.5) µm (n = 91), more frequently cylindrical; subterminal cells shorter, 3.5–8 µm wide, cylindrical or ellipsoid. **Pileocystidia**: near the pileus margin (23.5–)30.5–36–42(–53.5) × (2–)4–4.5–5(–5.5) µm (n = 29), rare, one-celled, predominantly fusiform, rarely cylindrical, originating in the suprapellis, thin-walled, apically obtuse, sometimes with a 1–3.5 µm long appendage; heteromorphous contents amorphous, sometimes only located in the apical part, clearly discernible in sulphovanillin, content insensitive, but cytoplasm turning to darker pink, also in the neighbouring cell. Pileocystidia near the pileus centre more frequent, similar in size, shape and heteromorphous contents to the ones near the pileus margin, (20.5–)32–39.5–47(–59) × (3–)4–4.5–5.5(–6.5) µm (n = 62). **Context**: without cystidioid and oleiferous hyphae.

##### Etymology.

For Flora, the daughter of the authors of this species.

##### Distribution and ecology.

Only known from the Kota gallery forest in Benin.

##### Notes.

Basidiomata of *R.roseoalba* without the fugitive fragile annulus are similar in field aspect to basidiomata of *R.florae*. The former species can be distinguished by its strongly positive reaction to guaiac and smooth pileipellis which is not regularly cracking in fine scales (Buyck 1994). Based on phylogenetic analyses, *R.roseoalba* is so far only known from the holotype collection. *Russulaacriuscula* Buyck is another species with pinkish-white pileus colours, but, as a member of subgen. Russula (subsect. Echinospermatinae Buyck), it is unrelated to “*Afrovirescentinae*”. It can be distinguished from *R.florae* by its acrid taste, more robust basidiomata and spores with isolated spines and amyloid suprahilar spot. *Russulapruinata* Buyck has a red pileus surface, but it can have whitish-farinaceous scales (probably from veil remnants) and also differs by its distinctly yellowing stipe context when bruised (Buyck 1994).

**Figure 18.**
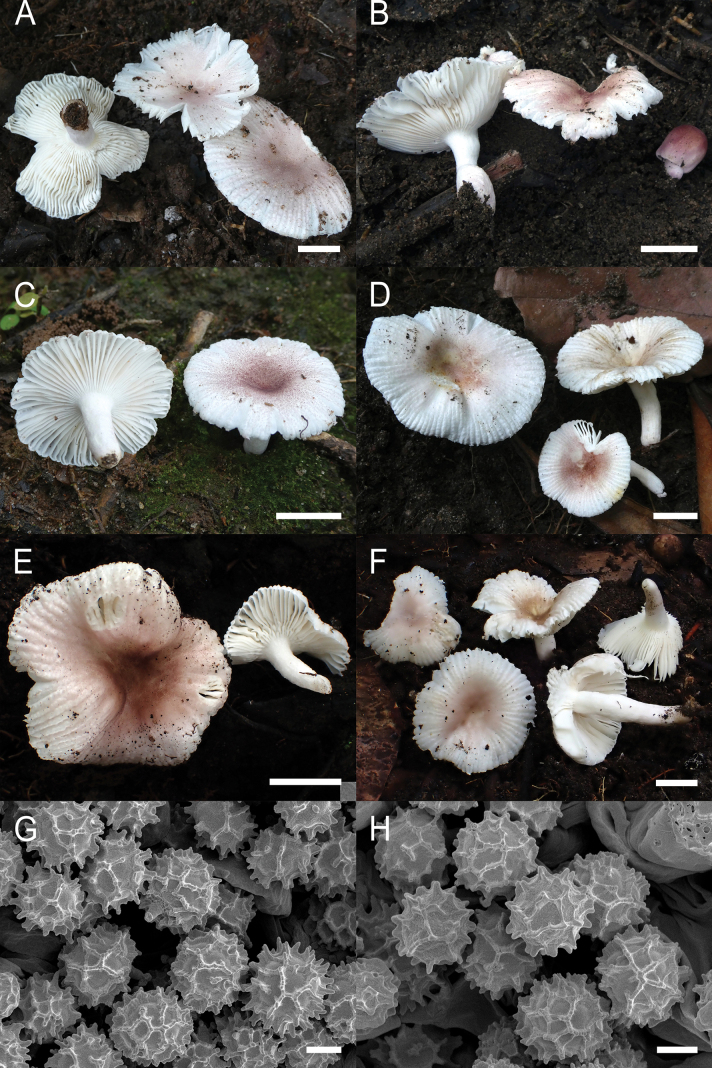
*Russulaflorae***A–F** field pictures of basidiomata: **A** CM-21-098, holotype, **B** CM-21-109, **C** CM-21-121, **D** CM-21-145, **E** CM-22-185, **F** CM-22-284; **G, H** scanning electron microscopical pictures of basidiospores (both CM-21-093, holotype). Scale bars: 1 cm (**A–F**); 3 µm (**G, H**).

**Figure 19.**
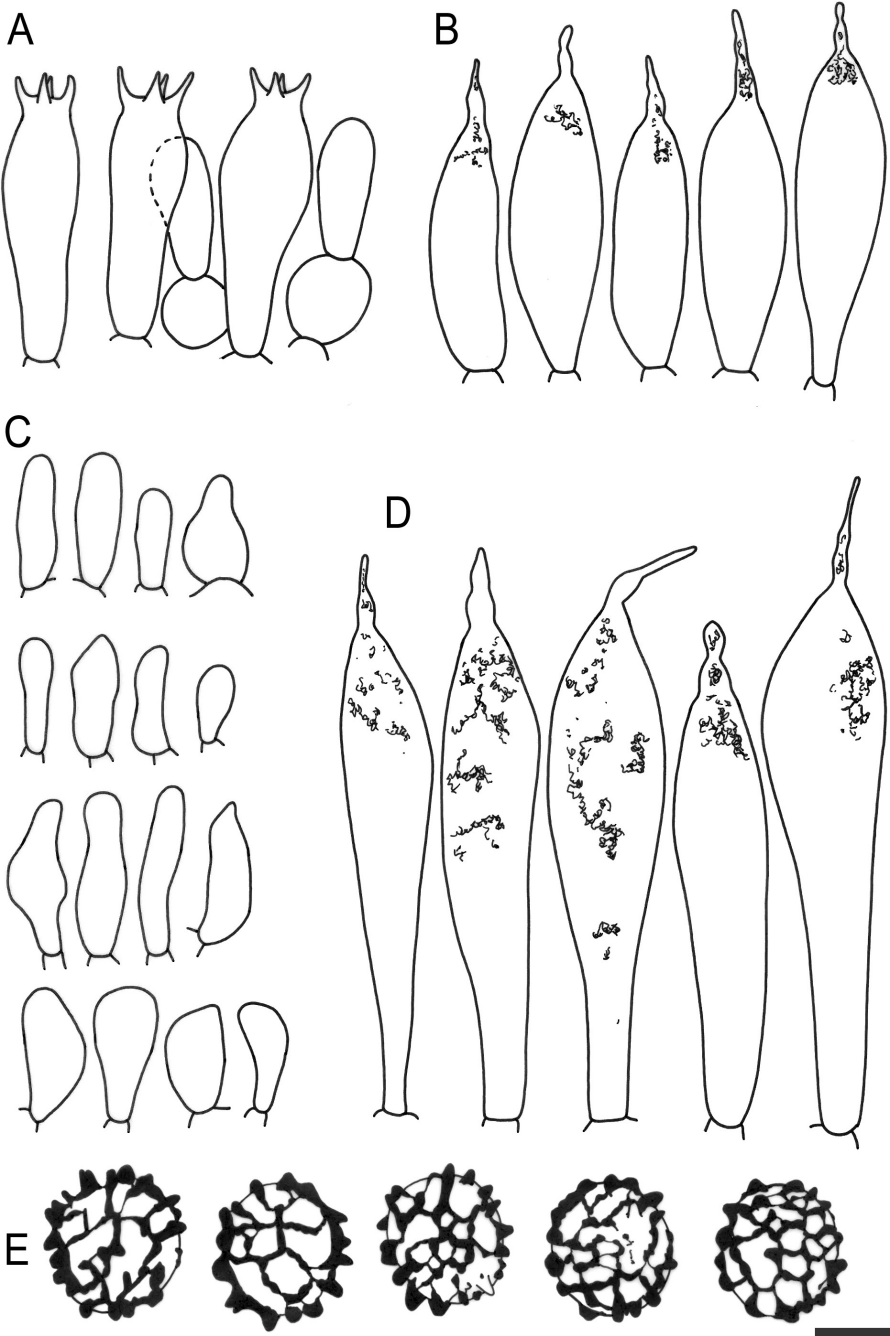
Hymenial elements of *Russulaflorae* (holotype, CM-21-098) **A** basidia and basidiola, **B** hymenial cystidia near the lamellar edges, **C** marginal cells, **D** hymenial cystidia, **E** spores as seen in Melzer’s reagent. Scale bar: 10 μm, but only 5 μm for spores.

**Figure 20.**
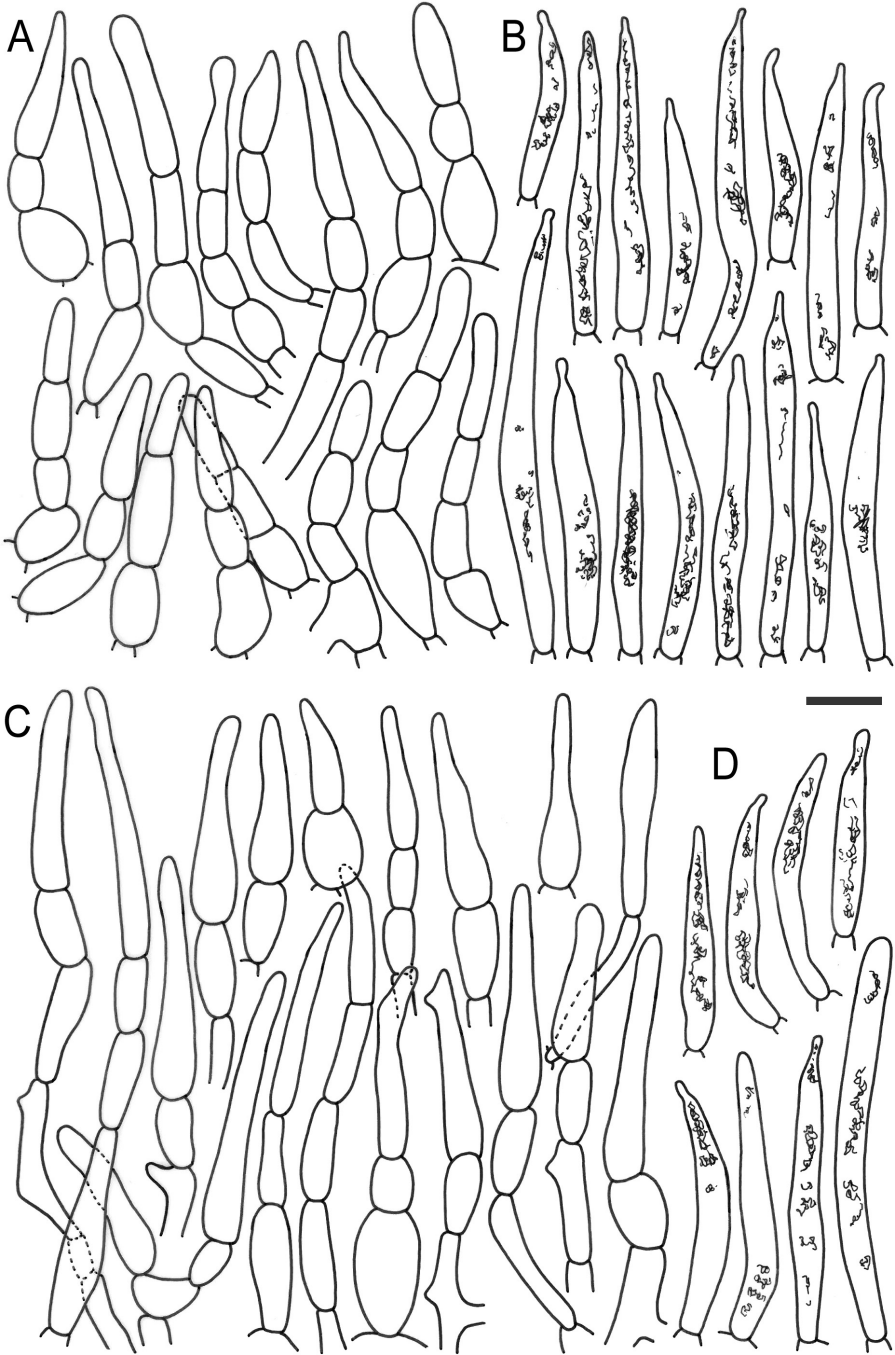
Elements of the pileipellis of *Russulaflorae* (holotype, CM-21-098) **A, B** elements near the pileus centre: **A** hyphal terminations, **B** pileocystidia; **C, D** elements near the pileus margin: **C** hyphal terminations, **D** pileocystidia. Scale bar: 10 μm.

#### ﻿ Russula hiemisilvae

Taxon classificationAnimaliaRussulalesRussulaceae

Buyck, Karstenia 33: 27 (1993)

D39A9978-2343-50C4-8022-B2215086211D

361062

Figs 21 , 22 , 23

##### Holotype.

Tanzania. Western Province, Kahama District, 30 km W of Kahama, Wendele, Forest Reserve (03 32 CB), alt. 1,200 m, on soil in *Brachystegia* Benth.-*Combretum* Loefl. woodland, 09.12.1991, leg. Saarimtiki et al. 1028 (H 7041854).

##### Additional material examined.

Benin. Atakora, Kossoucoingou, co-ord. 10°9.9'N, 1°12.1'E, alt. 500 m, Sudanian woodland, under *Isoberliniatomentosa*, on rocky soil, 20.07.2021, leg. C. Manz, F. Hampe, G. Abohoumbo, T.C. Bogo, *CM-21-150* (B 70 0105430, UNIPAR).

##### Short description.

*Russulahiemisilvae* is a rather robust, mild, annulate species with a greyish-red pileus, stipe with rose hue and ellipsoid spores with a reticulate ornamentation with spines up to 1.5 µm high, occurring in savannah woodlands.

##### Description based on material recently collected in Benin.

**Growth habit**: basidiomata in small groups. **Pileus**: medium-sized, 50–55 mm in diam., plane, slightly centrally depressed; margin even, striate up to 15 mm, regularly shaped, finely crenulate; cuticle smooth, radially fibrous, slightly shiny, peelable up to ½ of the pileus radius, colour near the margin pinkish-white (11A2), pale red (11A3) or greyish-rose (11B3), near the centre, dull red (11C3–4), greyish-brown (11D3) or greyish-red (11D4), sometimes with lighter spots. **Lamellae**: 5–6 mm wide, 7–8 lamellae present along 1 cm near the pileus margin, narrowly adnate, white, furcations, anastomoses and lamellulae absent; edges, concolourous. **Stipe**: 40–45 × 12–15 mm, cylindrical, sometimes narrowing towards the base, slightly bulging here and there; smooth to slightly rugose, with a fugacious white and dull red (11C3–4) rimmed annulus, white with a rose hue; cottony stuffed. **Context**: 1 mm thick at half pileus radius, white, unchanging when bruised, brittle, taste mild, odour inconspicuous; macrochemical reactions: guaiac after 8–10 seconds positive (++) on both stipe and lamellae surfaces, FeSO_4_ rose, sulphovanillin negative, KOH discolouring red parts to yellow, phenol negative. **Spore print**: not observed, but probably white or cream.

**Spores**: (8.3–)8.6–9–9.4(–9.9) × (7.3–)7.5–7.7–7.9(–8) µm (n = 30), Q = (1.08–)1.13–1.17–1.22(–1.26), subglobose to broadly ellipsoid; ornamentation of moderately distant amyloid spines [3–6(–7) in a circle of 3 µm diam.], 1.1–1.5 µm high, connected by abundant, distinct lines [3–6(–7) in the circle], forming a complete reticulum with regularly-shaped meshes, isolated elements absent; suprahilar plage small, inamyloid, without ornamentation, surrounded by small warts. **Basidia**: (41.5–)44.5–49–53.5(–59) × (12–)13–13.5–14(–14.5) µm (n = 20), subclavate to clavate, 4-spored; basidiola approx. 7–9 µm wide, clavate to subclavate. **Hymenial cystidia**: on lamellae sides (70.5–)77–86–95.5(–101) × (11–)12–13.5–15(–16) µm (n = 20), widely dispersed, 27–82/mm^2^, cylindrical to subclavate, sometimes slightly constricted, originating in subhymenium and somewhat protruding over basidia, thin-walled, apically obtuse, with a 2–7(–9) µm long appendage; heteromorphous contents dense, amorphous, mostly located in the upper half, turning distinctly dark yellow-brown in sulphovanillin. Hymenial cystidia near the lamellae edges shorter and narrower, (52.5–)56–61–66.5(–73.5) × (9–)10–11.5–13(–14.5) µm (n = 20), predominantly fusiform, with a 1.5–8 µm long appendage; heteromorphous contents less dense and less frequently located in the upper half. **Lamellae edges**: fertile, marginal cells intermixed with basidia and basidiola. **Marginal cells**: (30–)33–38–43(–46) × (3.5–)5.5–6.5–8(–9) µm (n = 20), cylindrical to subclavate with frequent irregular constrictions, frequently flexuous, optically empty, thin-walled. **Pileipellis**: orthochromatic in Cresyl blue, well delimited from the underlying context, 135–200 mm deep; suprapellis 50–110 µm deep, of loose, irregularly orientated hyphal terminations embedded in a gelatinous matrix; sharply delimited from a 70–85 µm deep subpellis of dense, parallel, 2.5–4.5 µm wide hyphae, with cystidioid hyphae with crystalline contents. Acid resistant encrustations absent. **Hyphal terminations**: near the pileus margin composed of 1–2(–3) unbranched cells, thin-walled, terminal cells (16.5–)22–26–30(–33) × (3–)3.5–4 µm (n = 30), cylindrical or slightly narrowing towards the apex, rarely subcapitate, apically obtuse, rarely acute; subterminal cells shorter, sometimes isodiametric, 3–4.5 µm wide, cylindrical. Hyphal terminations near the pileus centre of 1(–2) unbranched cells, thin-walled, terminal cells (6.5–)12–19–26(–33) × (2.5–)3.5–4–5(–6) µm (n = 37), irregularly shaped, cylindrical or subulate, frequently with several constrictions or branched, apically obtuse; subterminal cells usually shorter, 3.5–7.5 µm wide, cylindrical or ellipsoid, often irregularly shaped and with lateral branches or nodes. **Pileocystidia**: near the pileus margin (21–)24–31.5–39(–45.5) × (3.5–)4–4.5–5.5(–6) µm (n = 20), one-celled, lanceolate, cylindrical or subclavate, originating in the suprapellis, thin-walled, occasionally with a 2–3 µm long appendage or terminal knob; heteromorphous contents amorphous, weakly turning grey-violet in sulphovanillin. Pileocystidia near the pileus centre similar in size and shape to the ones near the pileus margin, (21.5–)25.5–29.5–33.5(–39.5) × (3.5–)4.5–5.5–6.5(–7.5) µm (n = 20), more frequently with a 2–4 µm long appendage or terminal knob; heteromorphous contents similar. **Context**: without cystidioid and oleiferous hyphae.

##### Distribution and ecology.

Widely distributed in tropical African savannah woodlands. Known from Benin, Burundi, Madagascar, Tanzania, Zambia and Zimbabwe.

##### Notes.

The material of *R.hiemisilvae* from Benin was identified, based on type sequencing. The holotype material of *R.hiemisilvae* differs from our collection by the presence of refractive inclusions in the hyphal terminations in the pileipellis and the reddish to almost absent reaction of the cystidia to sulphovanillin (Härkönen et al. 1993). *Russulaannulata* is a similar annulate species described from Madagascar, which differs from *R.hiemisilvae* by a stipe covered by vivid violet pustules, lamellae with dark violet edges and violet context under the cuticle (Heim 1937, 1938).

**Figure 21.**
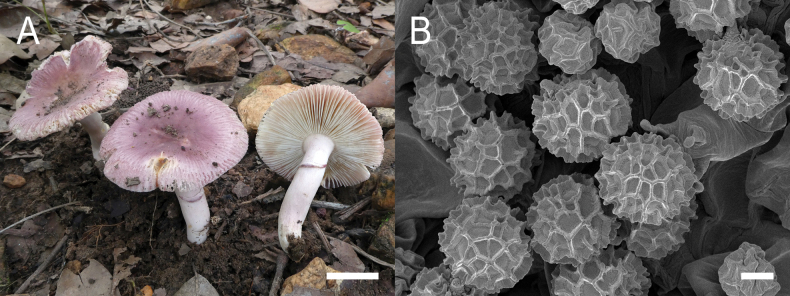
*Russulahiemisilvae* (CM-21-150) **A** field picture of basidiomata, **B** scanning electron microscopical picture of basidiospores. Scale bar: 2 cm (**A**); 3 µm (**B**).

**Figure 22.**
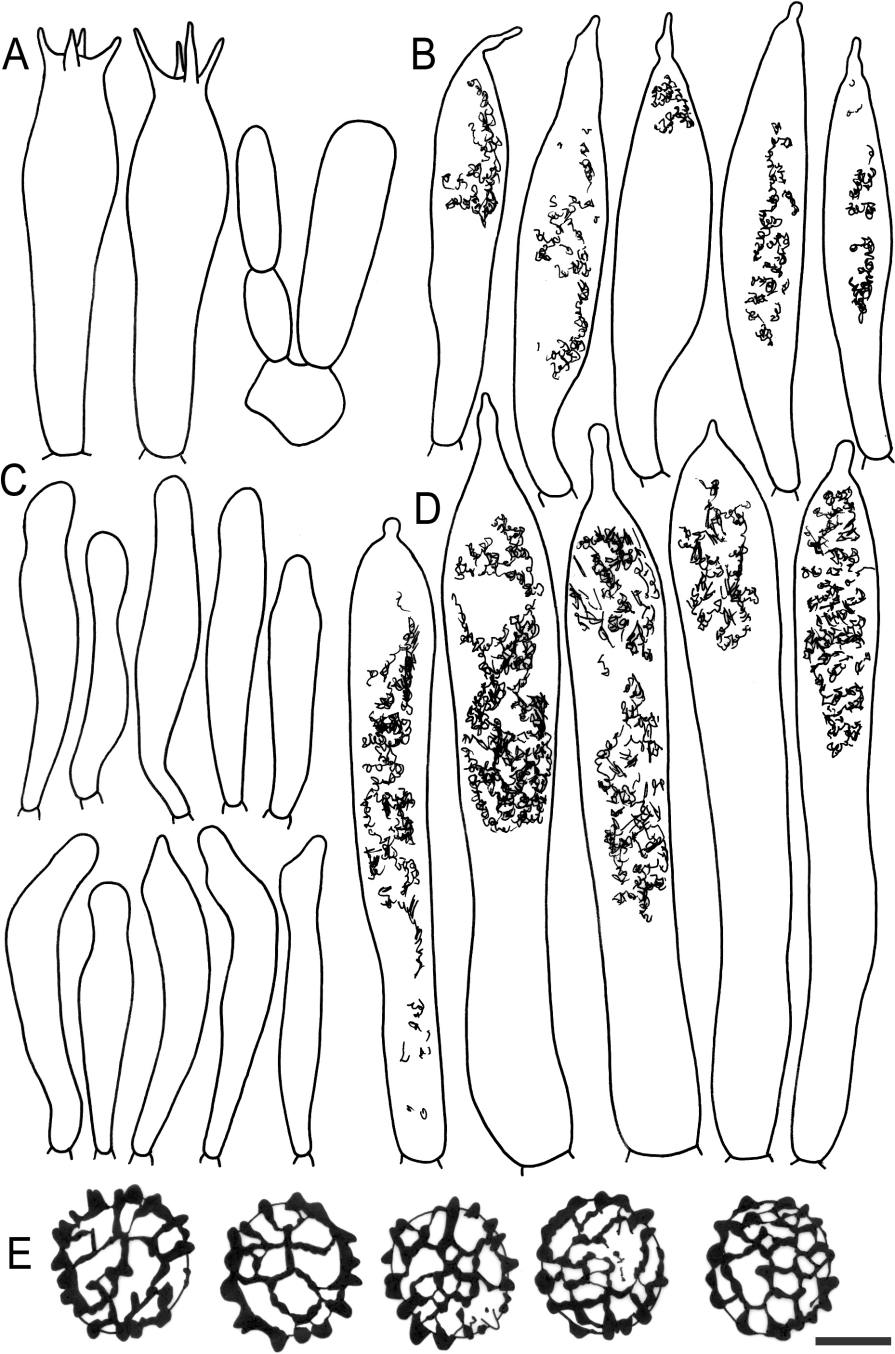
Hymenial elements of *Russulahiemisilvae* (CM-21-150) **A** basidia and basidiola, **B** hymenial cystidia near the lamellar edges, **C** marginal cells, **D** hymenial cystidia, **E** spores as seen in Melzer’s reagent. Scale bar: 10 μm, but only 5 μm for spores.

**Figure 23.**
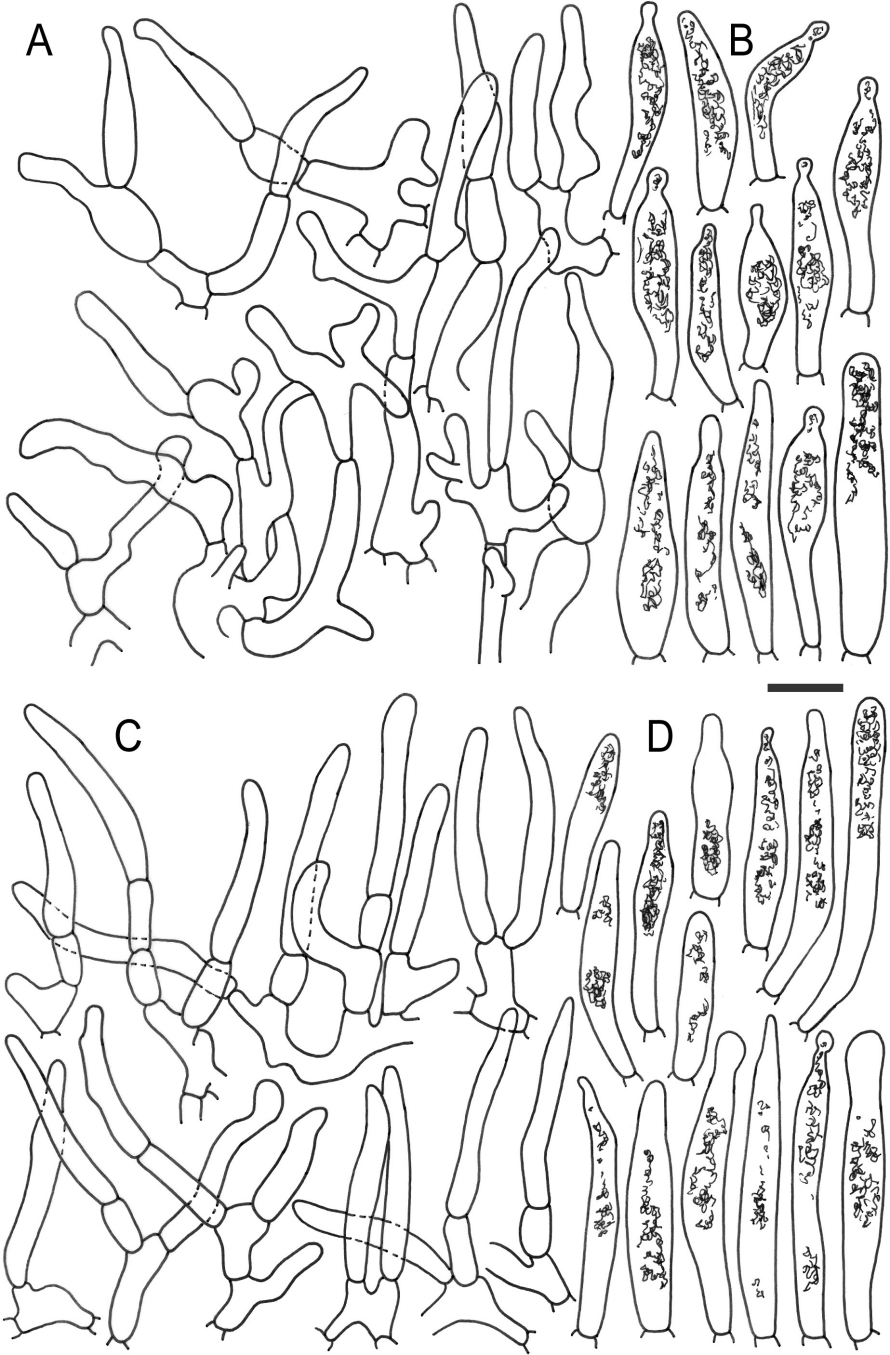
Elements of the pileipellis of *Russulahiemisilvae* (CM-21-150) **A, B** elements near the pileus centre: **A** hyphal terminations, **B** pileocystidia; **C, D** elements near the pileus margin: **C** hyphal terminations, **D** pileocystidia. Scale bar: 10 μm.

#### ﻿ Russula inflata

Taxon classificationAnimaliaRussulalesRussulaceae

Buyck, Bull. Jard. Bot. Natl. Belg. 58 (3–4): 470 (1988)

4AE91A52-8578-5ED2-9D80-3A153137D6A3

134759

**Figure 24.**
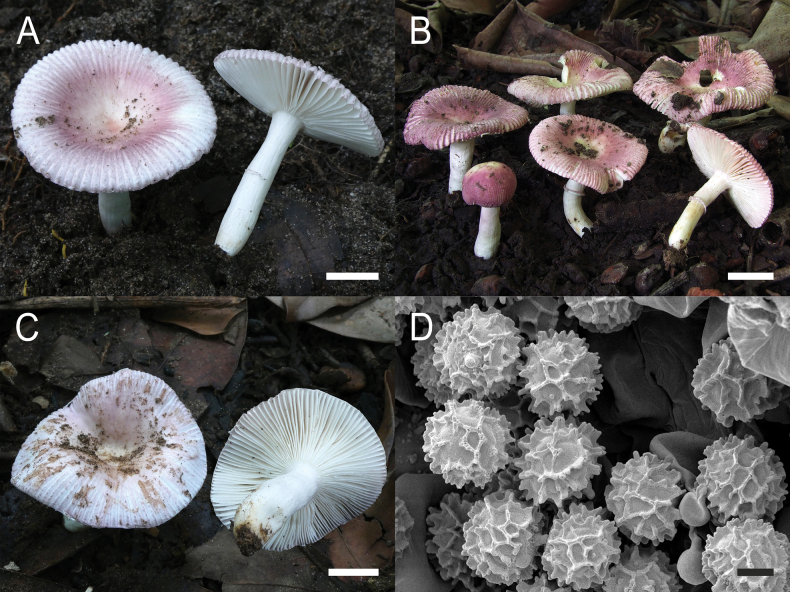
*Russulainflata***A–C** field pictures of basidiomata: **A** CM-21-144, **B** CM-22-231, **C** CM-21-128, **D** scanning electron microscopical picture of basidiospores (CM-21-144). Scale bar: 1 cm (**A, C**); 2 cm (**B**); 3 µm (**D**).

**Figure 25.**
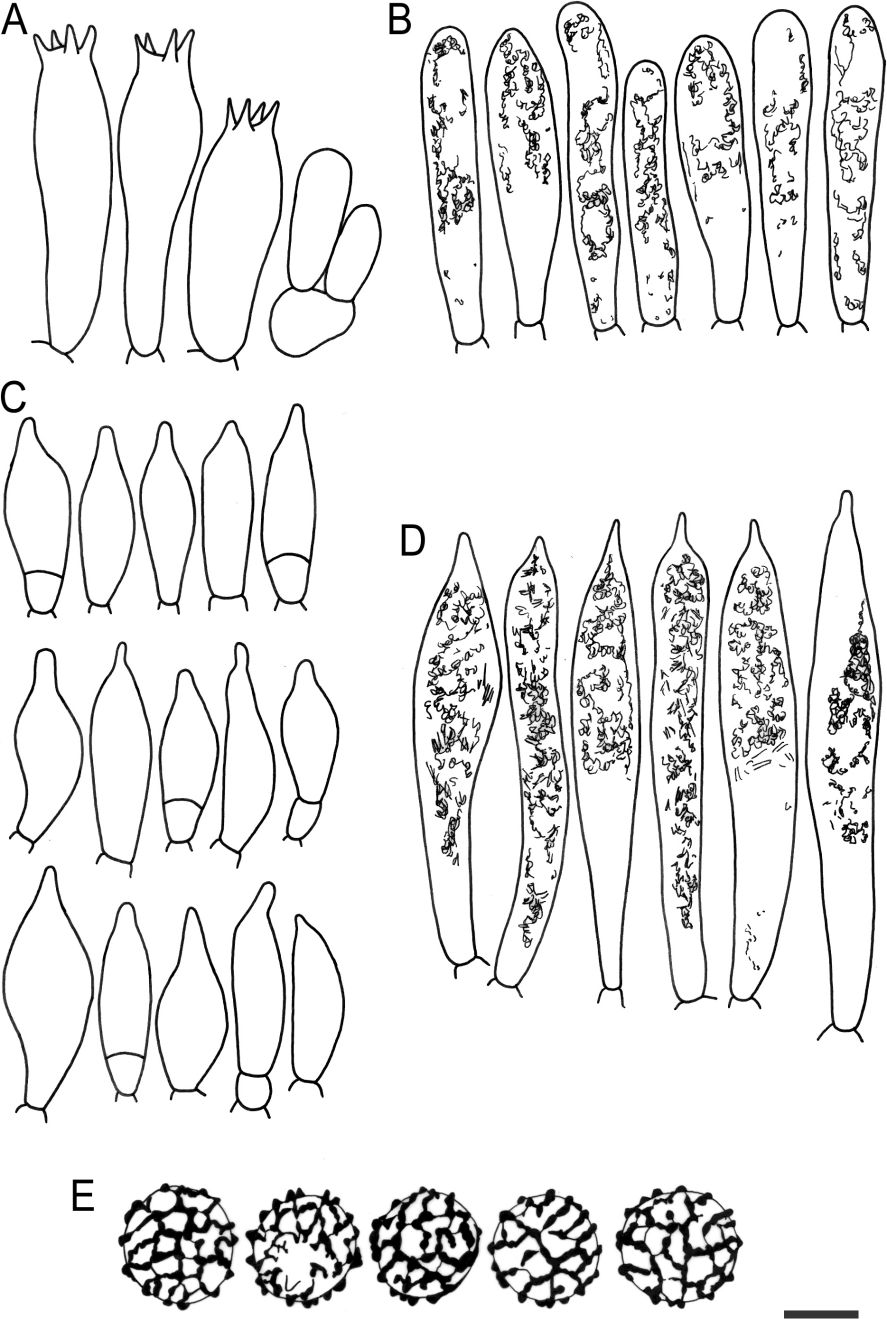
Hymenial elements of *Russulainflata* (CM-21-144) **A** basidia and basidiola, **B** hymenial cystidia near the lamellar edges, **C** marginal cells, **D** hymenial cystidia, **E** spores as seen in Melzer’s reagent. Scale bar: 10 μm, but only 5 μm for spores.

**Figure 26.**
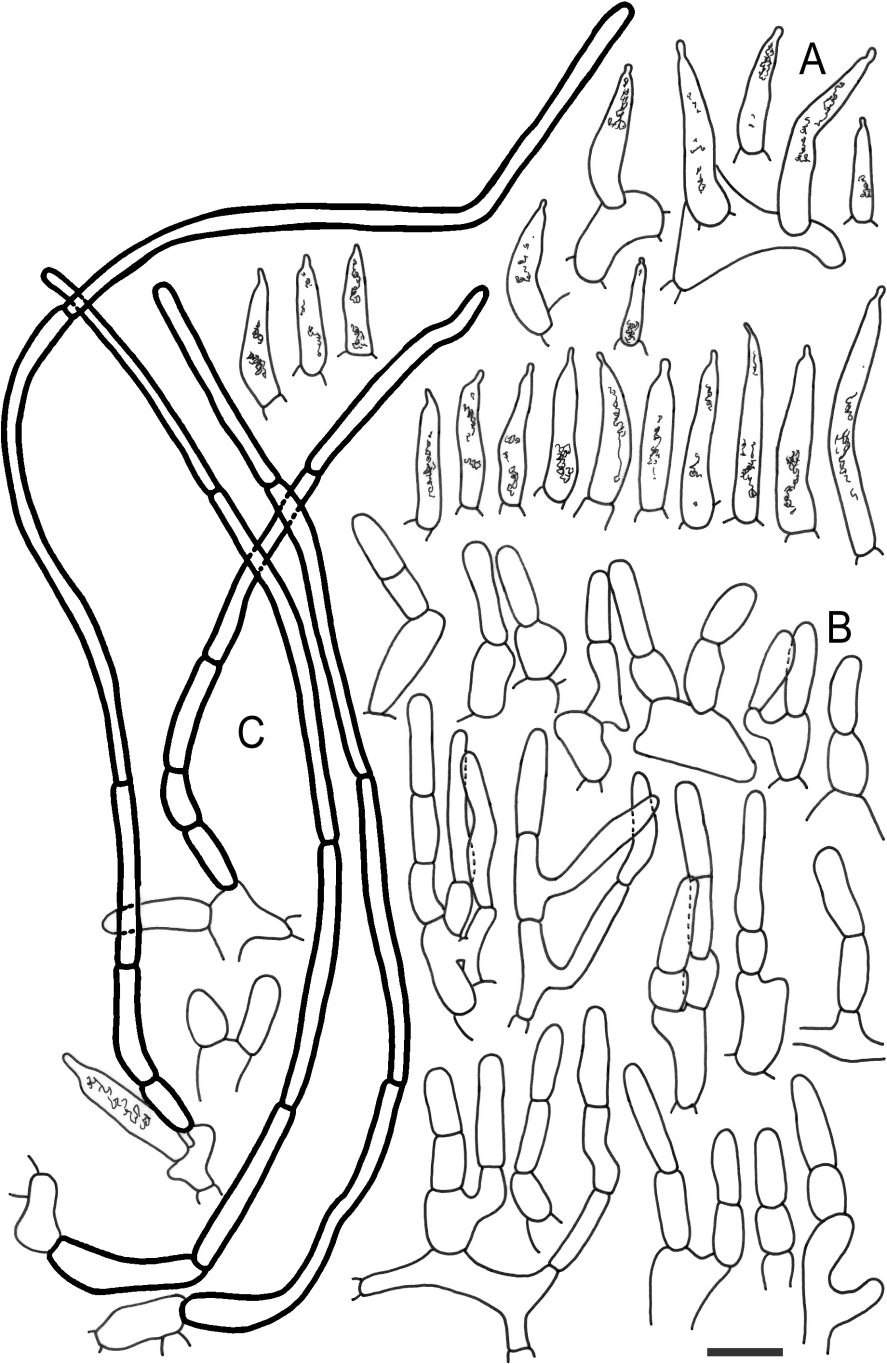
Elements of the pileipellis near the pileus centre of *Russulainflata* (CM-21-144) **A** pileocystidia, **B** hyphal terminations, **C** thick-walled hyphal terminations. Scale bar: 10 µm.

**Figure 27.**
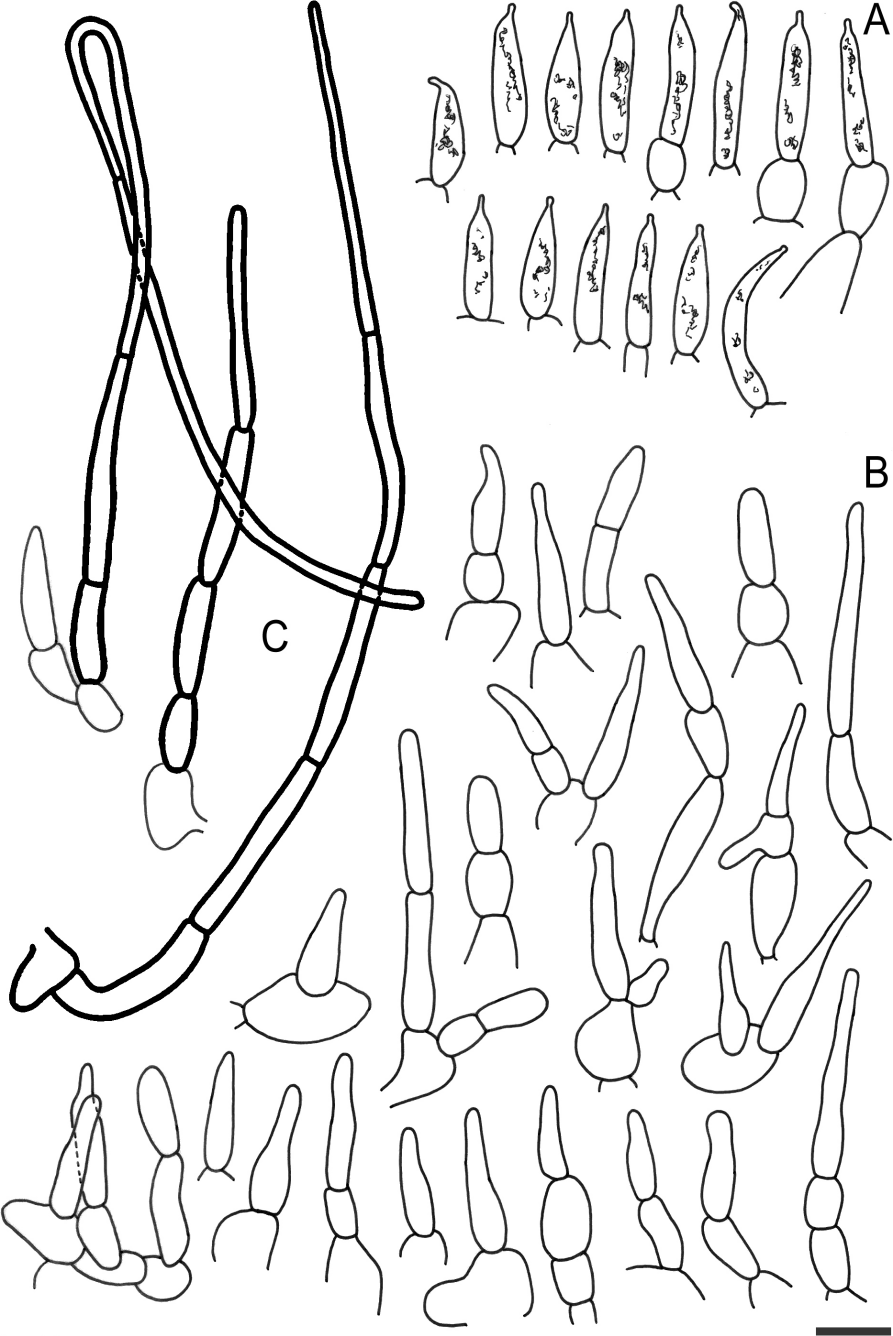
Elements of the pileipellis near the pileus margin of *Russulainflata* (CM-21-144) **A** pileocystidia, **B** hyphal terminations, **C** thick-walled hyphal terminations. Scale bar: 10 µm.

**Figure 28.**
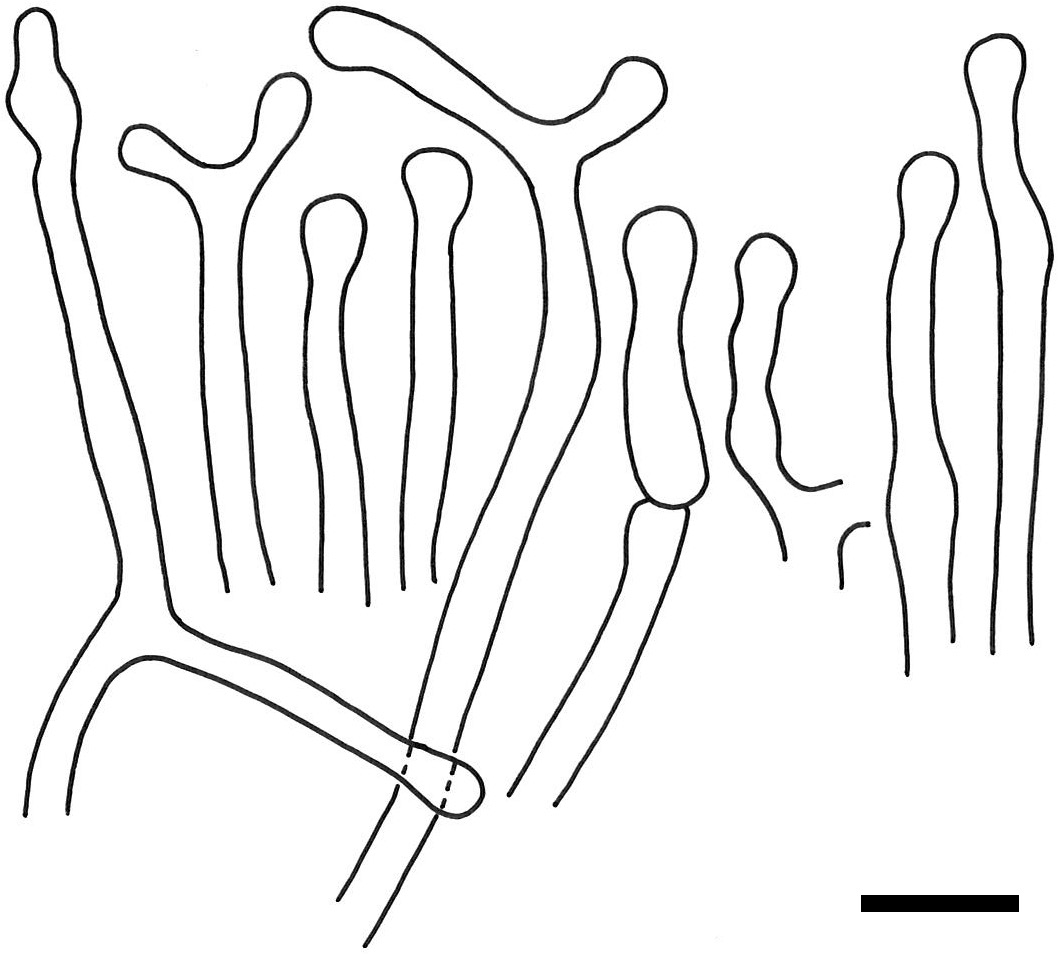
*Russulainflata* (CM-21-144). Hyphal terminations of the subpellis. Scale bar: 10 µm.

Figs 24 , 25 , 26 , 27 , 28

##### Holotype.

DRC. Kisangani; NNE van Batiabongena, primary rainforest under *Gilbertiodendrondewevrei* (De Wild.) J.Léonard, 05.05.1984, leg. B. Buyck, *1652* (**holotype** of *R.inflata*, BR5020005254166).

##### Synonym.

*Russulaintricata* Buyck, Bulletin du Jardin Botanique National de Belgique 58 (3–4): 470 (1988). **Holotype** – DRC. Luiswishi, dense forest, on ground, 20.04.1986, leg. J. Schreurs, *1778* (BR5020005258201).

##### Additional material examined.

Benin. Atakora, Natitingou, Kota Waterfalls, co-ord. 10°12.7'N, 1°26.8'E, alt. 500 m, in a gallery forest, under *Uapacaguineensis*, directly along the riverside on bare sand, 14.07.2021, leg. C. Manz, F. Hampe, N. S. Yorou, G. Abohoumbo & D. Dongnima, *CM-21-128* (B 70 0105431, UNIPAR); *ibid.* 19.07.2021, *CM-21-144* (B 70 0105432, UNIPAR); *ibid.* Kossoucoingou, co-ord. 10°9.5'N, 1°9.5'E, alt. 330 m, savannah woodland with dense undergrowth, under *Uapaca* sp., on rocky soil, 27.06.2022, leg. C. Manz, S. Sarawi, A. Rühl, *CM-22-231* (B 70 0105433, UNIPAR). DRC. Luiswishi, at the margin of a gallery forest, at the foot of an abandoned termite mound, 1990, leg. J. Degreef, *90/9* (as *R.roseoalba*, BR 5020005039923); *ibid.* South of firm Kimba, 18.04.1986, leg. J. Schreurs, *1738* (as *R.roseoalba*, BR5020006035214); *ibid.* 19.03.1986, leg. J. Schreurs, *1415* (as *R.roseoalba*, BR5020006037232); *ibid.* Muhulu de la Luiswishi, dense dry forest, 27.12.1971, leg. D. Thoen, *5209* (as *R.roseoalba*, BR5020006031179); *ibid.* Kisangani; NNE van Batiabongena, primary rainforest under *G.dewevrei*, 05.05.1984, leg. B. Buyck, *1653* (BR5020005255170); *ibid*. primary rainforest with *G.dewevrei* and *Scaphopetalum* Mast., 05.05.1984, leg. B. Buyck, *1560* (BR5020005256184). Senegal. Basse Casamance National Park, Guinean forest, 24.08.1986, leg. D. Thoen, *7647* (as *R.roseoalba*, BR5020006032183).

##### Short description.

Basidiomata small to medium-sized, pileus surface pinkish, stipe with fugitive annulus, hyphal terminations in the pileipellis of two types including presence of distinctly thick-walled, sparsely septate, very long hyphae repent over other elements of the suprapellis, occurring in gallery forests and savannah woodlands.

##### Description based on recent material from Benin.

**Growth habit**: solitary or gregarious. **Pileus**: small to medium-sized, 23–62 mm in diam., when young, convex, later expanding plane, centrally slightly to distinctly depressed; margin even or slightly involuted, distinctly tuberculate-striate up to 17 mm, crenulate, sometimes undulate or radially cracked; cuticle smooth, matt, sometimes with velar patches, distinctly radially cracked between the striations, sometimes easily and sometimes hardly peelable, colour near the margin pinkish-white (11–13A2), rose (11–12A3) or greyish-magenta (13B3, 13D3), near the centre, pinkish-white (11–12A2), dull red (11B3), greyish-ruby (12C4, 12D3–4), greyish-rose (12B4), but also yellowish-white (3A2). **Lamellae**: 3–4 mm wide, 4–8 lamellae present along 1 cm near the pileus margin, adnexed, white, furcations occasional near the stipe attachment, anastomoses and lamellulae absent; edges entire, concolourous. **Stipe**: 28–50 × 4–13 mm, cylindrical, sometimes clavate, smooth to slightly rugose, with a fugitive, white or rose (11A3) rimmed annulus that is sometimes attached to the pileus margin instead of the stipe, white or with a slight pinkish hue; cottony stuffed, with age becoming hollow. **Context**: up to 1 mm thick at half pileus radius, white, unchanging when bruised, brittle, taste mild, odour inconspicuous, macrochemical reactions: guaiac after 8–10 seconds weakly positive (+) or negative (-) on stipe and positive (++) on lamellae surfaces, FeSO_4_ weak orange or pinkish, sulphovanillin negative, KOH discolouring yellowish on red cuticle parts, phenol negative. **Spore print**: not observed, but probably white or cream.

**Spores**: (6.9–)7.3–7.7–8.1(–8.9) × (6.6–)7–7.3–7.7(–8.4) µm (n = 90), Q = (1–)1.02–1.05–1.08(–1.13), globose to subglobose; ornamentation of distant to moderately distant amyloid warts [(2–)3–5(–7) in a circle of 3 µm diam.], 1–1.4 µm high, connected by abundant lines [(2–)3–5(–6) in the circle] forming a complete reticulum, isolated warts or fusions dispersed (up to 1 in the circle), lines frequently rimmed by additional smaller warts only visible by SEM; suprahilar plage small, inamyloid, without ornamentation. **Basidia**: (30–)34.5–39.5–45(–52) × (10.5–)11.5–12.5–13.5(–15.5) µm (n = 60), cylindrical, clavate to broadly clavate, 4-spored; basidiola approx. 5–11 µm wide, cylindrical to subclavate. **Hymenial cystidia**: on lamellae sides (58–)65–72.5–80(–91) × (7–)9–11–13(–17) µm (n = 60), widely dispersed, 65–90/mm^2^, predominantly fusiform, rarely subclavate, sometimes slightly curved near the base, originating in subhymenium and somewhat protruding over basidia, thin-walled, apically acute, with a 2–10(–13) µm long appendage; heteromorphous contents dense, amorphous to crystalline, sometimes located only in the upper half, turning moderately, but distinctly greyish-black in sulphovanillin after 5–10 minutes. Hymenial cystidia near the lamellae edges shorter and narrower, (34–)41–46–51(–58) × (6–)7.5–9.5–11.5(–15) µm (n = 60), cylindrical to clavate, apically obtuse, without appendages, heteromorphous contents similar to those in hymenial cystidia on lamellae sides, but not crystalline. **Lamellae edges**: sterile, densely covered with marginal cells. **Marginal cells**: (19–)23–27–31(–36.5) × (6­–)7–9–11.5(–15) µm (n = 60), fusiform or lageniform, acute or with a narrow appendage, frequently with a secondary septum, optically empty, thin-walled. **Pileipellis**: orthochromatic in Cresyl blue, sharply delimited from the underlying context, 65–120 µm deep; suprapellis a trichoderm, 30–70 µm deep, composed of predominantly erect, loose hyphal terminations embedded in a gelatinous matrix; gradually passing to a 25–50 µm deep subpellis, of dense, more or less parallel, not gelatinised, thin-walled, 4.5–9 µm wide hyphae with capitate and frequently forked terminations, not gelatinised. Acid resistant encrustations absent. **Hyphal terminations**: near the pileus margin dimorphic; mainly thin-walled, up to 50 µm long, composed of 1–2(–3) unbranched cells, terminal cells (9–)12.5–18.5–24.5(–31) × (2.5–)3.5–4.5–5–(–7) µm (n = 93), predominantly subulate with obtuse apex, less frequently cylindrical; subterminal cells shorter, 3.5–7 µm wide, cylindrical or ellipsoid; additionally mixed with dispersed, thick-walled, sparsely septate, filamentous, curved or bent, repent, up to 200 µm long hyphal terminations. Hyphal terminations near the pileus centre composed of equally represented thin and thick-walled forms, thin walled composed of (1–)2–3 unbranched cells, terminal cells (7–)9–16.5–24(–37) × (2.5–)3.5–4–5(–6.5) µm (n = 93), stout, cylindrical; subterminal cells similar, 3–6.5 µm wide; thick-walled ones similar to those near the pileus margin. **Pileocystidia**: near the pileus margin (15–)17–19.5–22.5(–26) × (3–)3.5–4–5(–6.5) µm (n = 60), one-celled, predominantly fusiform, sometimes subcylindrical, rarely curved, originating in the suprapellis, thin-walled, apically obtuse, with a 1–3 µm long terminal knob; heteromorphous contents amorphous, not reacting to sulphovanillin. Pileocystidia near the pileus centre longer, more abundant, (11.5–)18–26.5–35(–48) × (2.5–)3.5–4.5–5(–7) µm (n = 60), shape and heteromorphous contents similar to those in pileocystidia near the pileus margin. **Context**: without cystidioid and oleiferous hyphae.

##### Distribution and ecology.

Widely distributed in rainforests, gallery forests and savannah woodlands in tropical Africa. Associated with *Asteropeiamcphersonii* G.E.Schatz, M.Lowry & A.-E.Wolf, *Gilbertiodendrondewevrei* and *Uapaca* spp. Known from Benin, Cameroon, DRC, Gabon, Madagascar and Senegal.

##### Notes.

*Russulainflata* is the type species of the subsect.Inflatinae defined by a pileipellis composed of a dense layer of intricate hyphae with irregular orientation and frequently inflated hyphal terminations (Buyck 1994). Based on this pileipellis morphology, *R.intricata* and *R.roseoalba* were also placed in this subsection. Sequences (ITS) of both type specimens are available as a result of this study. Since phylogenetic and morphological differences are minor, we consider *R.intricata* as a synonym of *R.inflata*. The sequence variation of the *R.inflata* clade in the ITS phylogeny might be caused by intraspecific/intragenomic polymorphisms which seem rather common in fungi (Cedeño-Sanchez et al. 2024). Our observations proved that the species is morphologically more variable than previously thought, which explains why two different morphotypes of this species were described in the same publication as different species. The spore ornamentation is also variable and was up to 1.5 µm high in Benin material or up to 2.5 µm high in type material of *R.inflata*. We also observed variable marginal cells which were unbranched with secondary septa in material from Benin, rarely branched in type material of *R.intricata* or frequently branched in type material of *R.inflata*. The shape of the terminal cells in the pileipellis is distinctly different between the pileus centre and margin. The presence of thick-walled inflated hyphal terminations in the subpellis was underlined in the original description of *R.inflata*, but according to our observations, it is not a reliable character for the species identification because these elements can sometimes be thin-walled, subcapitate and not distinctly inflated. To understand these variations in morphology and sequence polymorphisms, sampling covering the entire ecological and geographical range of the lineage and providing better quality for performing a multi-loci analysis is essential. *Russularoseoalba* is a species with similar field appearance due to which it was frequently confused with *R.inflata* and, because of that, several collections identified as this species by Buyck (1994) had ITS sequences matching *R.inflata*. *Russularoseoalba* can be distinguished by the absence of the striking thick-walled, up to 200 µm long pileipellis hyphae.

#### ﻿ Russula mollicula

Taxon classificationAnimaliaRussulalesRussulaceae

nom. prov.

73625515-B6BD-5E4F-BD61-564B4A1C38BB

Figs 29 , 30 , 31

##### Material examined.

Benin. Donga, Bassila, co-ord. 9°0.1'N, 1°38.9'E, alt. 360 m, in a gallery forest, under *Berliniagrandiflora*, on sandy soil, 30.06.2022, leg. C. Manz, F. Hampe, S. Sarawi, A. Rühl & R. Dramani, *CM-22-244* (B 70 0105434, UNIPAR).

##### Short description.

Basidiomata small and ephemerous, pileus surface whitish to pale pinkish, pileocystidia distinctly inflated, broadly fusoid with dense crystalline contents and pileipellis a trichoderm composed of long, narrowly lanceolate terminal cells on top of short and inflated subterminal cells, occurring in gallery forests.

**Growth habit**: basidiomata solitary. **Pileus**: small, 20 mm in diam., plane, centrally deeply depressed; margin uplifted, strongly tuberculate-striate up to ½ of the pileus radius, somewhat undulate and slightly crenulate; cuticle smooth, matt, not peelable, colour near the margin white, near the centre pinkish-white (10A2). **Lamellae**: approx. 2 mm wide, 8–9 lamellae present along 1 cm near the pileus margin, narrowly adnate, white, furcations, anastomoses and lamellulae absent; edges entire, concolourous. **Stipe**: 27 × 4 mm, cylindrical, bulging here and there, smooth, annulus absent, white; hollow. **Context**: approx. 0.5 mm thick at half pileus radius, white, unchanging when bruised, brittle, taste mild, odour inconspicuous, macrochemical reactions not observed. **Spore print**: not observed, probably white or cream.

**Spores**: (9.4–)9.9–10.3–10.8(–11.3) × (9.1–)9.4–9.8–10.3(–10.7) µm (n = 30), Q = (1–)1.02–1.05–1.08(–1.11), globose to subglobose, ornamentation of distant amyloid spines (1–3 in a circle of 3 µm diam.), 1.8–2.6 µm high, abundantly connected by distinct lines [1–3(–5) in the circle] forming a complete reticulum, isolated elements absent, spines and line connections with frequent secondary warts only visible by SEM, suprahilar plage medium-sized, with a distinct central amyloid spot. **Basidia**: (32.5–)36–41.5–46.5(–49.5) × (12–)13–14–15(–16) µm (n = 20), broadly clavate, 4-spored; basidiola approx. 9–12 µm wide, clavate. **Hymenial cystidia**: on lamellae sides (83–)91.5–99.5–107.5(–109.5) × (13–)15–17–18.5(–21.5) µm (n = 20), widely dispersed, 100–135/mm^2^, fusiform, originating in subhymenium and somewhat protruding over basidia, thin-walled, apically obtuse, with a 4–10(–15) µm long appendage; heteromorphous contents predominantly densely crystalline, turning dark grey violet in sulphovanillin. Hymenial cystidia near the lamellae edges distinctly shorter and narrower, (47.5–)51–63–75(–88) × (10–)11–12.5–14.5(–15.5) µm (n = 20), shape and heteromorphous contents similar to the one on hymenial cystidia on lamellae sides. *Lamellae edges* sterile, densely covered with marginal cells. **Marginal cells**: (19–)20–32.5–45(–67) × (6–)7.5–8.5–9.5(–11) µm (n = 20), fusiform or lanceolate, sometimes with long appendages, optically empty, thin-walled. **Pileipellis**: orthochromatic in Cresyl blue, sharply delimited from the underlying context, 80–105 µm deep; suprapellis a trichoderm, 23–38 µm deep, composed of a thin layer of erect, not gelatinised hyphal terminations arranged in tufts; well delimited from the 55–73 µm deep subpellis, of loose, gelatinised, interwoven, irregularly orientated, 3–5 µm wide hyphae, becoming denser and horizontally arranged towards the context. Acid resistant encrustations absent. **Hyphal terminations**: near the pileus margin composed of (1–)2 unbranched cells, thin-walled, terminal cells (18.5–)27.5–37–46.5(–63.5) × (2.5–)3–3.5–4(–5) µm (n = 30), attenuated, subulate, slender, slightly flexuous, apically obtuse; subterminal cells distinctly shorter, 3.5–6.5 µm wide, cylindrical, ellipsoid or subglobose. Hyphal terminations near the pileus centre composed of up to 3 unbranched cells, terminal cells (15–)26.5–36–45.5(–56.5) × (2.5–)3–3.5–4.5(–5.5) µm (n = 30), similar in shape to the ones near the pileus margin; subterminal cells shorter, 3.5–10 µm wide, inflated, ellipsoid to subglobose. **Pileocystidia**: near the pileus margin (28–)30–39.5–49(–66) × (11–)12.5–14.5–16.5(–18) µm (n = 20), one-celled, inflated, broadly fusiform, originating in the suprapellis, thin-walled, apically obtuse, with a 3–16 µm long finger-like appendage; heteromorphous contents dense, crystalline, turning to grey, surrounding cytoplasm to dark pink in sulphovanillin. Pileocystidia near the pileus centre similar in size and shape to those near the pileus margin (25–)27.5–39–50(–66) × (11–)12–14–16.5(–19.5) µm (n = 20), with a single or rarely two, 3–18 µm long appendages; heteromorphous contents similar. **Context**: without cystidioid and oleiferous hyphae.

##### Etymology.

molliculus (lat.) = dainty, tender. Referring to the small and tender habit of the basidiomata of this species.

##### Distribution and ecology.

Only known from the Bassila gallery forest in Benin.

##### Notes.

So far, *R.mollicula* nom. prov. is the only “*Afrovirescentinae*” species with a consistently and distinctly amyloid spot on the suprahilar plage. Despite this striking diagnostic character and well-defined position in our phylogeny (Fig. 1), we refrain from formally describing it as a new species because we only studied a single collection. As the specimen was collected under very moist weather conditions, we were not certain if the species has a partial veil and we were unable to document the macrochemical reactions and the variability of the pileus colours. *Russularoseoalba* is very similar in field aspect, but differs by more slender pileocystidia with less crystalline contents and hyphal terminations in the pileipellis composed of longer chains of cells.

**Figure 29.**
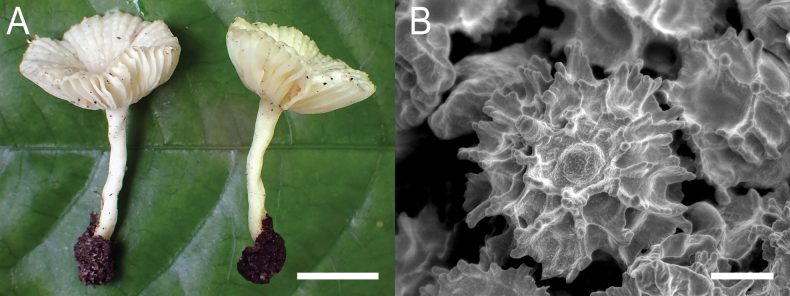
*Russulamollicula* nom. prov. (CM-22-244) **A** field picture, **B** scanning electron microscopical picture of basidiospore with a prominent spot on the suprahilar plage. Scale bar: 1 cm (**A**); 3 µm (**B**).

**Figure 30.**
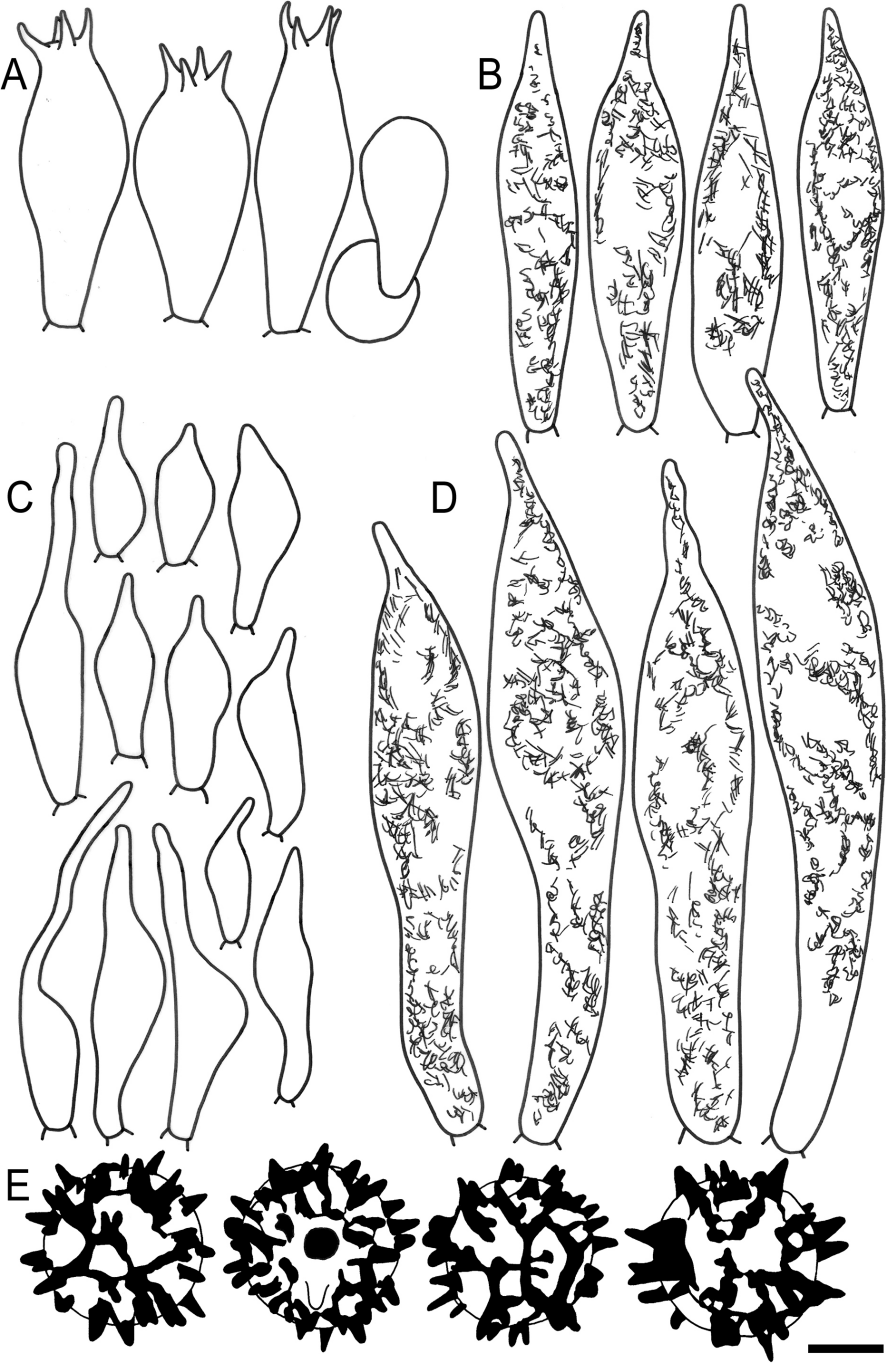
Hymenial elements of *Russulamollicula* nom. prov. (CM-22-244) **A** basidia and basidiola, **B** hymenial cystidia near the lamellar edges, **C** marginal cells, **D** hymenial cystidia, **E** spores as seen in Melzer’s reagent. Scale bar: 10 μm, but only 5 μm for spores.

**Figure 31.**
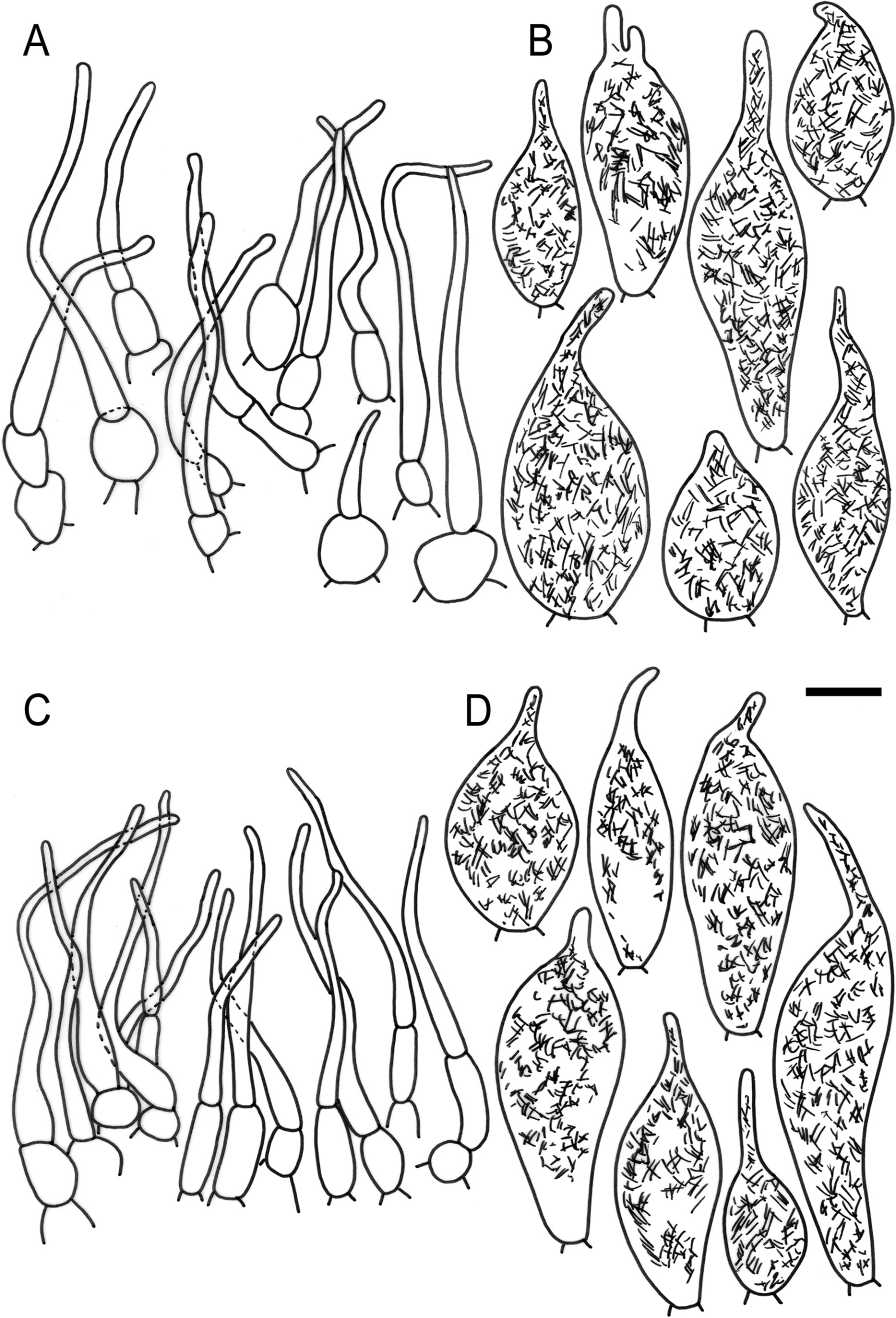
Elements of the pileipellis of *Russulamollicula* nom. prov. (CM-22-244) **A, B** elements near the pileus centre: **A** hyphal terminations, **B** pileocystidia; **C, D** elements near the pileus margin: **C** hyphal terminations, **D** pileocystidia. Scale bar: 10 μm.

#### ﻿ Russula spectabilis

Taxon classificationAnimaliaRussulalesRussulaceae

Manz, F. Hampe & Buyck, sp. nov.

8AA73A2E-BA0C-5857-BFD8-57D4BAAAF95C

856867

Figs 32 , 33 , 34

##### Holotype.

Benin. Atakora, Kossoucoingou, co-ord. 10°9.9'N, 1°12.1'E, alt. 500 m, Sudanian woodland, under *Isoberliniatomentosa*, on rocky soil, 20.07.2021, leg. C. Manz, F. Hampe, G. Abohoumbo, T.C. Bogo, *CM-21-154* (**holotype** B 70 0105435, **isotype**UNIPAR).

##### Additional material examined.

Benin. Atakora, Natitingou, Kota Waterfalls, co-ord. 10°12.7'N, 1°26.8'E, alt. 500 m, Sudanian woodland, under *I.tomentosa*, on rocky soil, 12.07.2021, leg. C. Manz, F. Hampe, N. S. Yorou & G. Abohoumbo, *CM-21-116* (**paratype**, B 70 0105436, UNIPAR).

##### Diagnosis.

Basidiomata relatively robust, pileus surface yellow-orange, taste mild, terminal cells of the pileipellis hyphae lanceolate near the pileus margin and narrowly cylindrical near the pileus centre, occurring in savannah woodlands. Differs from *Russulaaureola* Buyck by a lower spore ornamentation.

##### Description.

**Growth habit**: basidiomata solitary. **Pileus**: medium-sized to large, 80–90 mm in diam., soon expanding plane, centrally depressed; margin even, finely tuberculate-striate up to 10 mm, regularly shaped; cuticle smooth, matt, peelable up to ¾ of the pileus radius, colour butter yellow (4B5), near the margin pale orange (5A3) or light orange (5A4), near the centre, melon (5A6) or golden yellow (5B7). **Lamellae**: 6–7 mm wide, 8–9 lamellae present along 1 cm near the pileus margin, narrowly adnate, white to pale cream, furcations frequent at the stipe attachment, anastomoses absent, lamellulae occasionally present; edges entire, concolourous. **Stipe**: 90–100 × 18–22 mm, cylindrical, smooth to slightly rugose, annulus absent, white; cottony stuffed, cavernate with 2–3 distinct chambers. **Context**: 6–7 mm thick at half pileus radius, white, unchanging when bruised, brittle, taste mild, odour inconspicuous to pleasant, macrochemical reactions: guaiac after 8–10 seconds (++) on stipe and weakly positive (+) on lamellae surfaces, FeSO_4_ weak salmon orange, sulphovanillin negative, KOH negative or slightly discolouring on the pileipellis, phenol negative. **Spore print**: not observed, probably white or cream.

**Spores**: (6.9–)7.4–7.7–8.1(–8.7) × (6.1–)6.4–6.7–7(–7.4) µm (n = 60), Q = (1.03–)1.10–1.15–1.20(–1.26), subglobose to broadly ellipsoid; ornamentation of moderately distant, dense to very dense, pustules [(5–)6–9(–12) in a circle of 3 µm diam.], not well defined, by light microscopy up to 0.5 µm high, connected by abundant, short lines [(2–)3–6(–8) in the circle] forming a complete reticulum, isolated or partially connected elements very rare to absent, occasionally to frequently fused (approx. 1–4 fusions in the circle); suprahilar plage small, inamyloid, without ornamentation. **Basidia**: (36.5–)42–47.5–53(–58) × (9–)10–11–12(–12.5) µm (n = 40), narrowly to broadly clavate, 4-spored; basidiola approx. 5–8 µm wide, cylindrical to clavate. **Hymenial cystidia**: on lamellae sides (60.5–)71–83.5–95.5(–114) × (8.5–)9.5–10.5–11.5(–12.5) µm (n = 40), predominantly lanceolate, sometimes with slight moniliform constrictions, originating in subhymenium and somewhat protruding over basidia, thin-walled, apically obtuse, usually with a 2–13 µm long appendage; heteromorphous contents moderately dense, mostly amorphous, rarely crystalline, mostly located in the upper two-thirds, not reacting to sulphovanillin. Hymenial cystidia near the lamellae edges distinctly shorter and narrower, (38.5–)42.5–51–60(–70) × (6–)6.5–7.5–9(–11) µm (n = 40), lanceolate or rarely cylindrical, apically predominantly mucronate, with broad, 2.5–11 µm long appendages; heteromorphous contents similar to the one in hymenial cystidia on lamellae sides, but less dense. **Lamellae edges**: sterile, consisting of cystidia and marginal cells. **Marginal cells**: (23.5–)27.5–32.5–38(–48.5) × (4–)4.5–5.5–6.5(–8) µm (n = 40), narrowly utriform, lageniform or lanceolate, rarely cylindrical with slight constrictions, sometimes with a forked apex, optically empty, thin-walled. **Pileipellis**: orthochromatic in Cresyl blue, gradually passing to the underlying context, 125–165 µm deep; suprapellis a trichoderm, 30–40 µm deep, composed of erect hyphal terminations; gradually passing to a 95–130 µm deep subpellis of loose, strongly gelatinised, intricate, irregularly orientated, 1.5–4 µm wide hyphae, becoming denser and horizontally arranged near the context. Acid resistant incrustations absent. **Hyphal terminations**: near the pileus margin composed of 1–4 unbranched cells, thin-walled, frequently covered with a glutinous coating well visible in Congo red, terminal cells (8–)11.5–19.5–27(–40) × (2–)2.5–3–3.5(–4.5) µm (n = 60), predominantly lanceolate or subulate, rarely cylindrical, apically obtuse or acute; subterminal cells usually shorter, 3–6 µm wide, cylindrical or slightly inflated, branched or not. Hyphal terminations near the pileus centre composed of 1–3 unbranched cells, thin-walled, terminal cells shorter, (6.5–)9–13–17(–26) × 2–2.5–3(–3.5) µm (n = 61), slender, straight, cylindrical; subterminal cells equally long, 2–3.5 µm wide. **Pileocystidia**: near the pileus margin (15–)27–37.5–47.5(–55.5) × (3–)3.5–4.5–5.5(–7.5) µm (n = 44), one-celled, cylindrical or clavate to subcapitate, rarely lanceolate, flexuous, slightly moniliform, originating in the suprapellis, thin-walled, occasionally with a 2–3 µm long appendage; heteromorphous contents amorphous, not reacting to sulphovanillin. Pileocystidia near the pileus centre similar in size, shape and heteromorphous contents to those near the pileus margin, (24.5–)32–40–48.5(–55) × (4–)4.5–5–5.5(–6.5) µm (n = 40). **Context**: without cystidioid hyphae, oleiferous hyphae dispersed.

##### Etymology.

Reference to the African plant *Costusspectabilis* (Fenzl) K.Schum., the flower of which is similarly coloured.

##### Distribution and ecology.

Only known from the Sudanian woodlands in Atakora in Benin.

##### Notes.

*Russulaaureola*, *Russulabonii* Buyck and *Russulasingeri*R. Heim, are other tropical African species with similar orange pileus colours. *Russulaaureola* has more slender basidiomata and was described from *Gilbertiodendrondewevrei* forests in the DRC, differing by a high spore ornamentation of 2 µm long spines and a pileipellis with attenuated terminal cells on top of a chain- of barrel-shaped subterminal cells (Buyck 1994). *Russulabonii* has relatively robust basidiomata with low spore ornamentation. It is known from miombo woodland in Zambia (Buyck 1995). It was placed in subsect.Amoeninae Singer ex Buyck because of the absence of both hymenial cystidia and pileocystidia (Buyck 1995). *Russulasingeri* is a mild species with white spore print which differs by a relatively tough, greying stipe and spores with an amyloid suprahilar plage (Heim 1937, 1938). *Russulatenuithrix* Buyck has a similar pileipellis structure and low spore ornamentation of up to 0.5 µm height, but differs by its greenish-grey pileus colours (Harkönen et al. 1993). Based on type sequencing, it is not related to *R.spectabilis* and probably belongs to Russulasubgen.Malodorae Buyck & V. Hofstetter. The type sequence of *R.tenuithrix* will be published in a future study on subgen.Malodorae.

**Figure 32.**
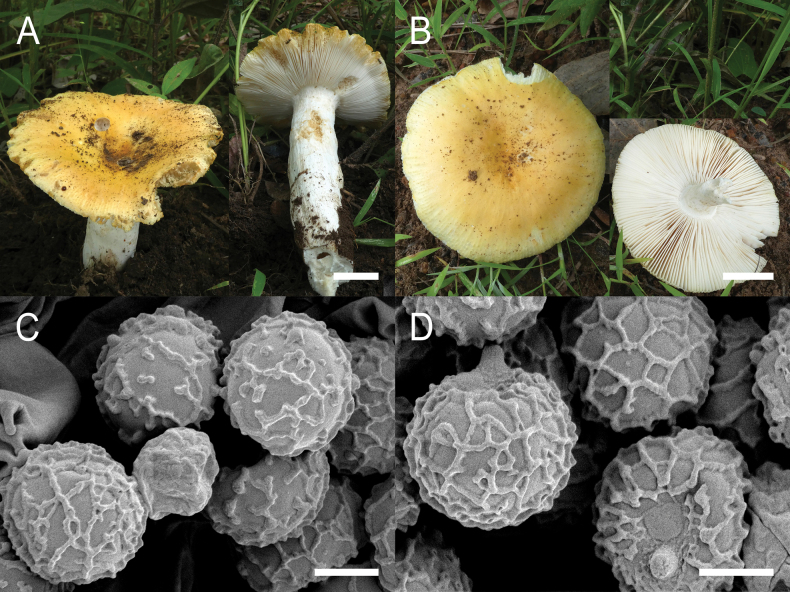
*Russulaspectabilis***A, B** field pictures of basidiomata: **A** CM-21-154, holotype, **B** CM-21-116; **C, D** scanning electron microscopical pictures of basidiospores (both CM-21-154, holotype). Scale bars: 2 cm (**A, B**); 3 µm (**C, D**).

**Figure 33.**
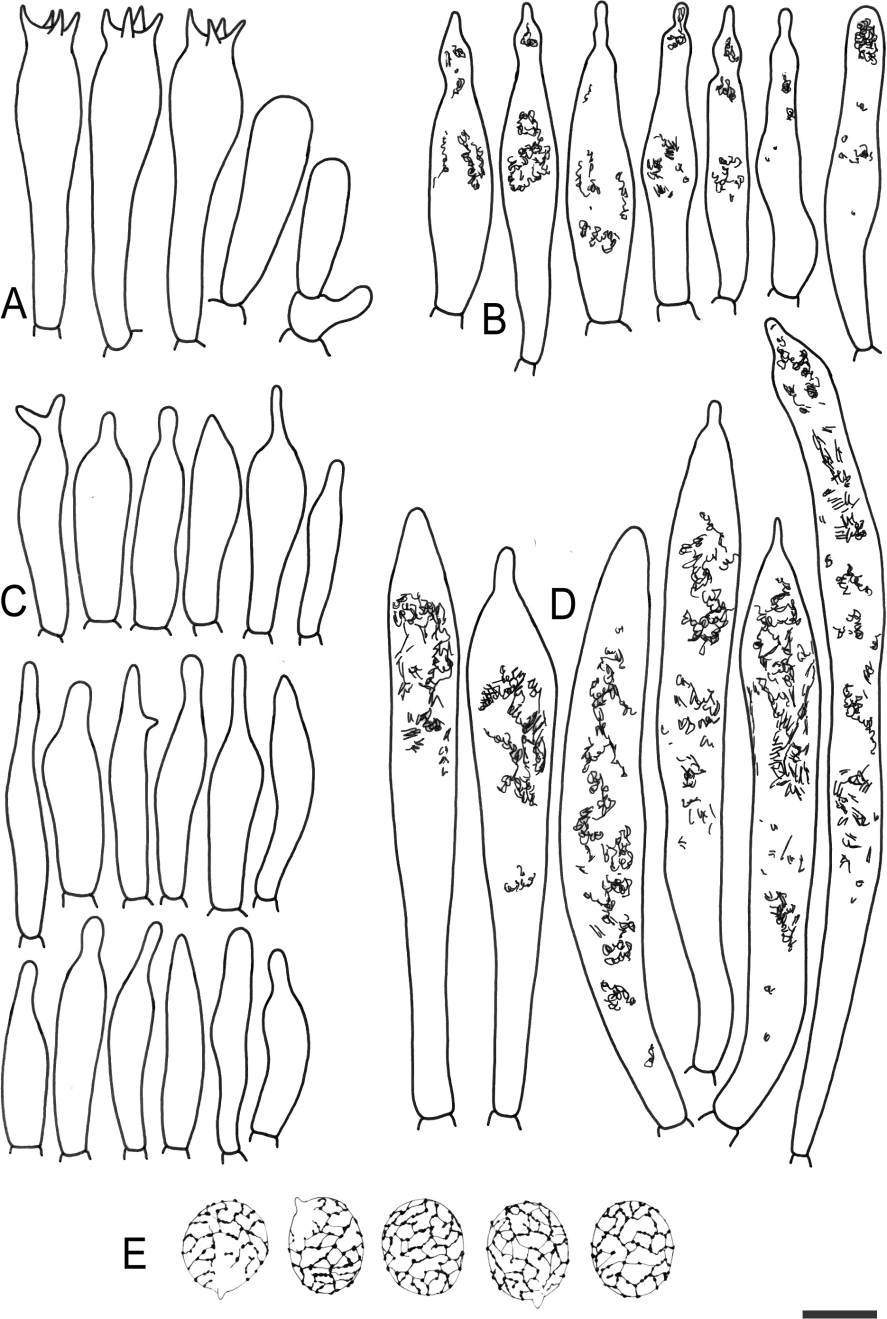
Hymenial elements of *Russulaspectabilis* (holotype, CM-21-154) **A** basidia and basidiola, **B** hymenial cystidia near the lamellar edges, **C** marginal cells, **D** hymenial cystidia, **E** spores as seen in Melzer’s reagent. Scale bar: 10 μm, but only 5 μm for spores.

**Figure 34.**
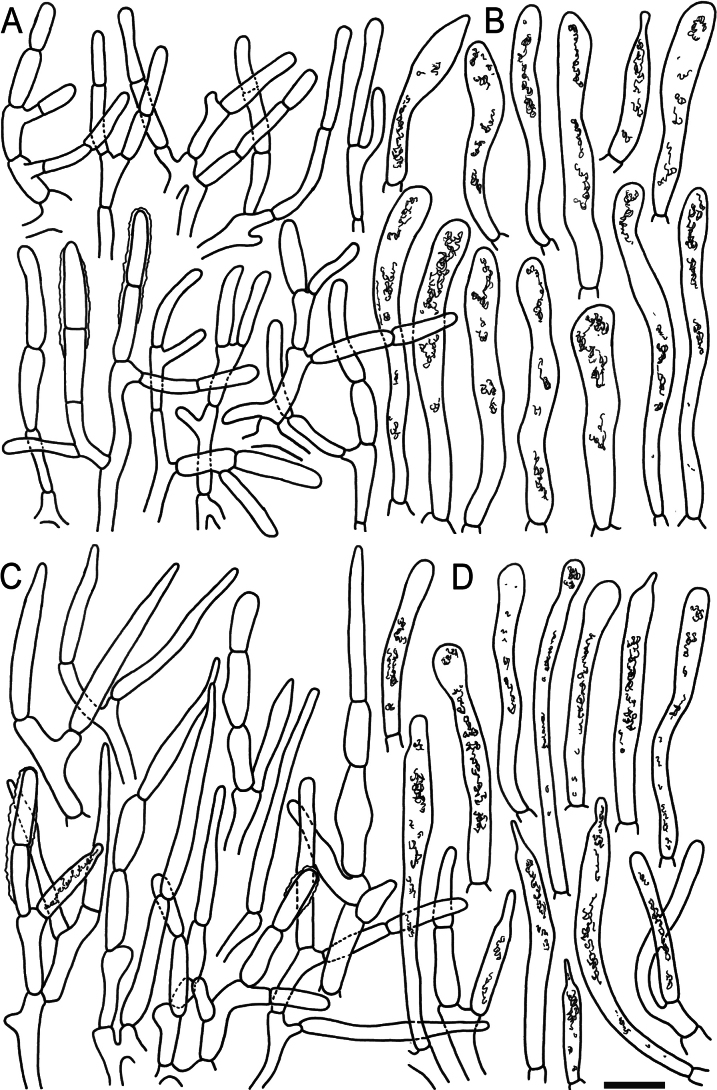
Elements of the pileipellis of *Russulaspectabilis* (holotype, CM-21-154) **A, B** elements near the pileus centre: **A** hyphal terminations, **B** pileocystidia; **C, D** elements near the pileus margin: **C** hyphal terminations, **D** pileocystidia. Scale bar: 10 μm.

#### ﻿ Russula sublaevis

Taxon classificationAnimaliaRussulalesRussulaceae

(Buyck) Buyck, Karstenia 33: 34 (1993)

71F274E8-52F9-5A86-A66B-5632167A28A7

361063

Figs 35 , 36 , 37

##### Holotype.

DRC. Haut-Katanga, Lubumbashi; 14 km from l’Bashi, at the roadside in open forest, 02.02.1986, leg.: J. Schreurs, Schreurs 985 (BR5020005285474).

##### Basionym.

Russularoseoviolaceaf.sublaevis Buyck, Bull. Jard. Bot. Natl. Belg. 60: 204 (1990).

##### Additional material examined.

Benin. Atakora, Natitingou, Kota Waterfalls, co-ord. 10°12.8'N, 1°26.8'E, alt. 500 m, Sudanian woodland, under *Isoberliniatomentosa*, on rocky soil, 19.07.2021, leg. C. Manz, F. Hampe, N. S. Yorou, G. Abohoumbo & D. Dongnima, *CM-21-148* (B 70 0105437, UNIPAR); *ibid.* co-ord. 10°12.7'N, 1°26.6'E, alt. 500 m, Sudanian woodland, under *I.tomentosa*, on rocky soil, 26.06.2022, leg. C. Manz, F. Hampe, S. Sarawi, A. Rühl & D. Dongnima, *CM-22-219* (B 70 0105438, UNIPAR); *ibid.* 05.07.2022, leg. C. Manz & F. Hampe, *CM-22-281* (B 70 0105439, UNIPAR); *ibid.* co-ord. 10°12.4'N, 1°26.9'E, alt. 470 m, in a gallery forest, under *Berliniagrandiflora* & *Isoberliniadoka*, on the ground, 08.06.2002, leg. A. De Kesel, *ADK3317* (BR5020152205127).

##### Short description.

*Russulasublaevis* is a species with medium-sized basidiomata, a bright yellow pileus, white stipe, mild taste and cream-coloured spore print. Microscopically, the small subglobose spores with a very low ornamentation are noticeable.

##### Description based on material recently collected Benin.

**Growth habit**: solitary or in groups of two. **Pileus**: medium-sized, 50–70 mm in diam., slightly convex to plane, centrally with a low shallow depression; margin even, finely striate up to 15 mm, regularly shaped; cuticle smooth, pruinose all over, under a magnifying glass with fine, whitish areolae, peelable up to ¾ of the pileus radius, colour yellow to bright yellow (3A2), becoming paler with age, near the centre sometimes slightly paler or darker. **Lamellae**: 5–6 mm wide, 8–9 lamellae present along 1 cm near the pileus margin, adnexed, at first white, then becoming pale cream, furcations and anastomoses absent, sometimes with dispersed lamellulae; edges entire, concolourous. **Stipe**: 35–45 × 8–10 mm, cylindrical, somewhat bulging here and there, smooth to slightly rugose, annulus absent, white; cottony stuffed, cavernate, with 2–3 distinct chambers. **Context**: 4–5 mm thick at half pileus radius, white, unchanging when bruised, brittle, taste mild, odour inconspicuous. Macrochemical reactions: guaiac after 8–10 seconds weakly positive (+) or negative (-) on stipe and positive (++) on lamellae surfaces; FeSO_4_ salmon orange, sulphovanillin negative; KOH negative; phenol negative. **Spore print**: cream (IIc).

**Spores**: (5.4–)6–6.3–6.7(–7.1) × (4.5–)5.1–5.4–5.6(–6) µm (n = 90), Q = (1.06–)1.12–1.18–1.24(–1.31), subglobose to broadly ellipsoid; surface almost smooth, ornamentation very inconspicuous, composed of very dense, weakly amyloid pustules and crests hardly visible under light microscope, ornamentation approx. 0.1 µm high as estimated by SEM, abundantly connected by fine lines forming a complete reticulum; few scattered isolated warts only visible by SEM; suprahilar plage small, inamyloid, partially covered by even lower ornamentation only visible by SEM. **Basidia**: (28.5–)33.5–37–40.5(–46.5) × (7.5–)8–8.5–9(–10.5) µm (n = 60), subcylindrical to narrowly clavate, 4-spored; basidiola approx. 4–6.5 µm wide, cylindrical to narrowly clavate. **Hymenial cystidia**: on lamellae sides (54.5–)60.5–69.5–78(–88.5) × (8–)9–11–13(–17) µm (n = 60), narrowly to distinctly clavate, rarely fusiform, sometimes slightly curved or bent at the base, originating in subhymenium and somewhat protruding over basidia, thin-walled, apically obtuse, sometimes with a 2.5–12 µm long appendage; heteromorphous contents amorphous, mostly located in the upper half, not reacting to sulphovanillin. Hymenial cystidia near the lamellae edges shorter and narrower, (39–)45.5–51.5–58(–67) × (7–)8.5–9.5–10.5(–12.5) µm (n = 60), narrowly to distinctly clavate, with a 2.5–13 µm long appendage, missing in some specimens; heteromorphous contents sparse, located in the apical part. **Lamellae edges**: fertile, with equal representation of cystidia, basidia, basidiola and marginal cells. **Marginal cells**: (15.5–)21.5–28–35(–42) × (3–)4–5–5.5(–7) µm (n = 60), predominantly fusiform, sometimes cylindrical or clavate, optically empty, thin-walled. **Pileipellis**: orthochromatic in Cresyl blue, sharply delimited from the underlying context, 200–300 µm deep; suprapellis a trichoderm, 40–50 µm deep, composed of erect, somewhat gelatinised hyphal terminations; gradually passing to a 160–250 µm deep, strongly gelatinised subpellis of loose, intricate, irregularly orientated, 2–3 µm wide hyphae, becoming gradually denser and horizontally arranged near the context. Acid resistant encrustations absent. **Hyphal terminations**: near the pileus margin composed of 2–4 unbranched cells, thin-walled, terminal cells (6–)20–32–44(–78.5) × (2.5–)3–4–5(–7) µm (n = 94), mainly subulate rarely cylindrical, apically obtuse; subterminal cells shorter, 3.5–4.5 µm wide, cylindrical. Hyphal terminations near the pileus centre similar to the ones near the pileus margin, terminal cells slightly narrower, (10.5–)16.5–29.5–43(–71) × (1.5–)2.5–3.5–4.5(–6) µm (n = 91); subterminal cells shorter, 2–5.5 µm wide, cylindrical. **Pileocystidia**: near the pileus margin (42–)48.5–59–69(–84.5) × (3.5–)4.5–5.5–6.5(–7.5) µm (n = 60), one-celled, predominantly lanceolate, sometimes subcylindrical, originating in the suprapellis, thin-walled, apically obtuse, with a 2–12 µm long appendage; heteromorphous contents amorphous, sometimes located only in the apical part, not reacting to sulphovanillin. Pileocystidia near the pileus centre slightly shorter, (34–)42–52–62(–75) × (3.5–)4.5–5.5–6.5(–8.5) µm (n = 60), similar in shape and heteromorphous contents to pileocystidia near the pileus margin. **Context**: without cystidioid hyphae, oleiferous hyphae frequent.

##### Distribution and ecology.

Widely distributed in Sudanian woodlands in tropical Africa. Kown from Benin, DRC and Zimbabwe.

##### Notes.

Here we provide the first detailed description of *R.sublaevis*, based on our recent collections from Benin which represent the first record of this species for the country. The identity of this material is confirmed by ITS sequences that are very similar to those of the holotype (Fig. 2). Morphologically, the Beninese collections differ from the holotype description by spores that are, on average, 1 µm shorter (Buyck 1994). Originally, *R.sublaevis* was described as a colour form of *R.roseoviolacea* with lower spore ornamentation, emphasising the similarity of pileipellis elements (Buyck 1990; Buyck 1994). Härkönen et al. (1993) formally combined the taxon to species rank on the occasion of a recent find from Tanzania. Buyck and Sharp (2007) reported the species from Zimbabwe from mixed miombo woodlands with *Julbernardiaglobiflora* (Benth.) Troupin and *Brachystegiaspiciformis* Benth., *Monotes* A.DC. The occurrence of *R.sublaevis* in Zimbabwe is also confirmed by a sequenced specimen (UDB07672946) collected in the Matobo National Park by Cathy Sharp. Further records of *R.sublaevis* from Tanzania, Togo and Malawi (https://www.gbif.org/ accessed on 30.06.2024, Härkönen et al. (1993)) remain unverified due to a lack of confirmation by corresponding sequence data and/or sufficient morphological descriptions. The species is widely distributed in sub-Saharan Africa in savannah habitats from western to eastern parts of the continent, in association with various host trees from the *Fabaceae* family.

**Figure 35.**
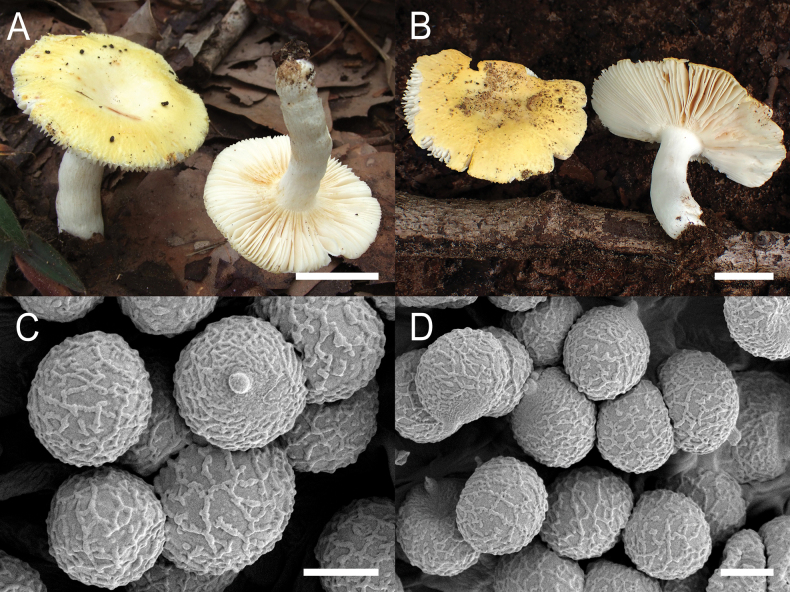
*Russulasublaevis***A, B** field pictures of basidiomata: **A** CM-21-148, **B** CM-21-219; **C, D** scanning electron microscopical pictures of basidiospores (both CM-21-148). Scale bars: 2 cm (**A, B**); 3 µm (**C, D**).

**Figure 36.**
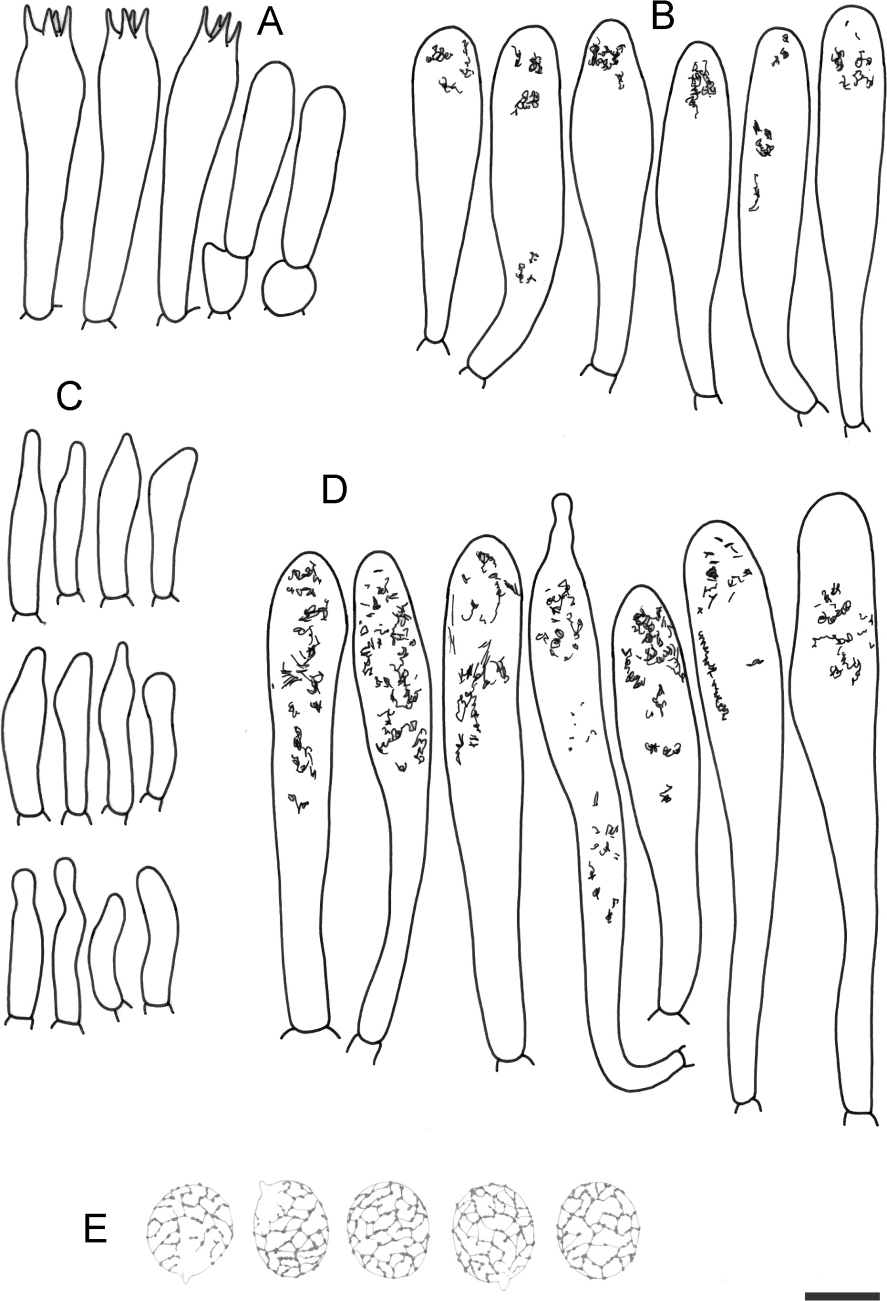
Hymenial elements of *Russulasublaevis* (CM-21-148) **A** basidia and basidiola, **B** hymenial cystidia near the lamellar edges, **C** marginal cells, **D** hymenial cystidia, **E** spores as seen in Melzer’s reagent. Scale bar: 10 μm, but only 5 μm for spores.

**Figure 37.**
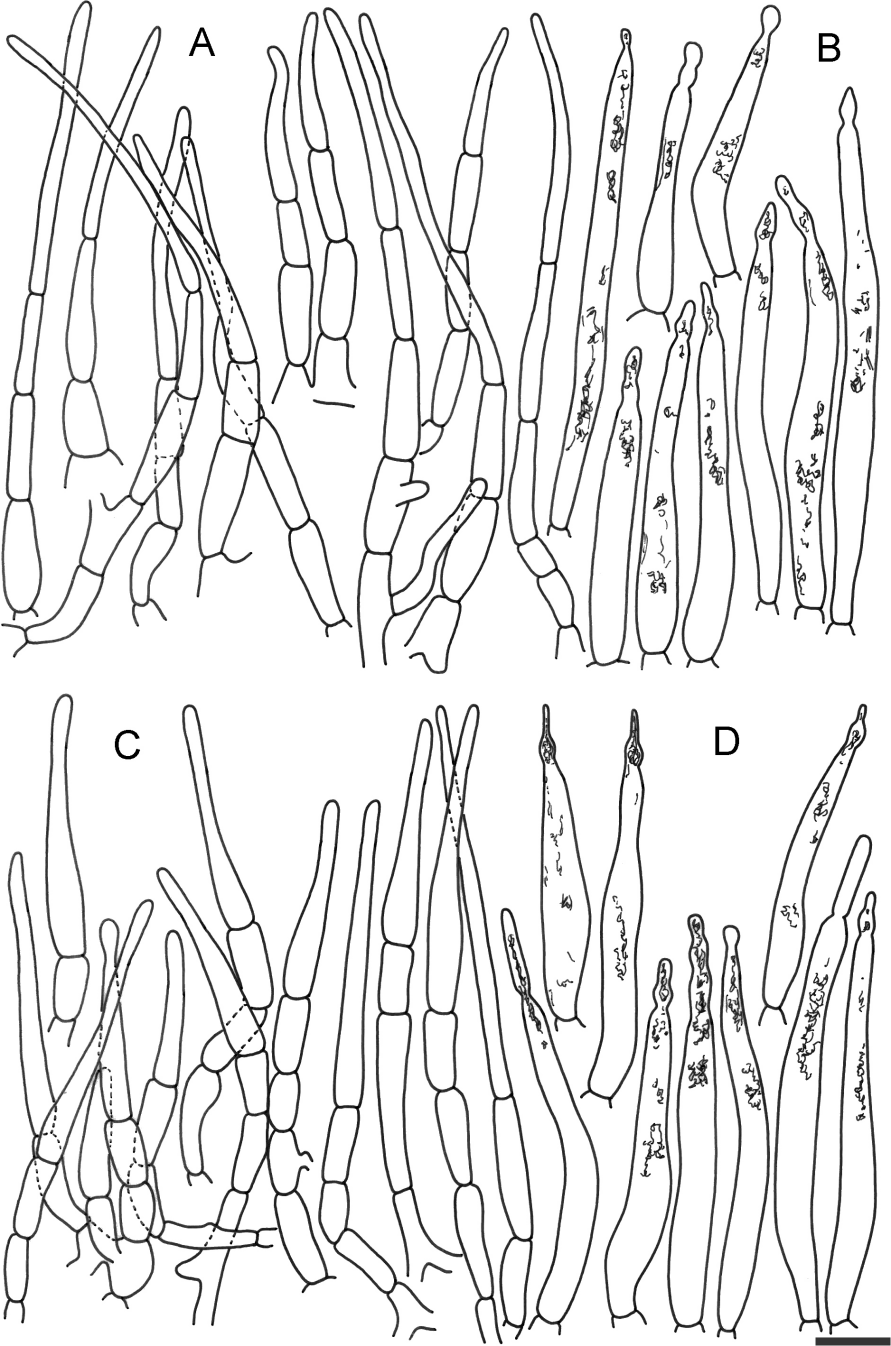
Elements of the pileipellis of *Russulasublaevis* (CM-21-148) **A, B** elements near the pileus centre: **A** hyphal terminations, **B** pileocystidia; **C, D** elements near the pileus margin: **C** hyphal terminations, **D** pileocystidia. Scale bar: 10 μm.

### ﻿Key to species of “*Afrovirescentinae*” known for Benin

**Table:** 

1	Basidiomata thin fleshed (context up to 1 mm thick at half pileus radius), mostly tiny (pileus diameter up to 40 mm), occurrence in gallery forests, spore ornamentation mostly prominent (> 1 µm high)	**2**
–	Basidiomata thick fleshed (context usually > 1 mm thick at half pileus radius), usually large (pileus diameter > 50 mm), occurrence in savannah woodlands, spore ornamentation mostly low (up to 0.5 µm high)	**6**
2	Pileus carmine red	***R.carmesina***
–	Pileus colour deep to pale violet or pink	**3**
3	Pileipellis with conspicuous long, thick-walled hyphal terminations	***R.inflata***
–	Thick-walled hyphal terminations in pileipellis absent	4
4	Basidiospores with a distinctly amyloid suprahilar spot	***R.mollicula* nom. prov.**
–	Basidiospores without amyloid suprahilar spot	**5**
5	Pileus 60–65 mm wide, greyish-violet, usually with corona-like veil remnants, marginal cells near lamellae edges diverticulate	***R.coronata***
–	Pileus 10–40 mm wide, usually pale pink with whitish margin, usually completely without veil remnants, marginal cells near lamellae edges inconspicuous	***R.florae***
6	Pileus colour white or pink	**7**
–	Pileus colour yellow or orange	**10**
7	Taste burning acrid	***R.acrialbida***
–	Taste mild	**8**
8	Pileus colour whitish, spore ornamentation < 0.5 µm high	***R.beenkenii***
–	Pileus colour deep pink, spore ornamentation > 1 µm high	**9**
9	Pileipellis with conspicuous long, thick-walled hyphal terminations	***R.inflata***
–	Thick-walled, long hyphal terminations in pileipellis absent	***R.hiemisilvae***
10	Terminal cells near the pileus centre cylindrical, pileocystidia usually without appendage, pileus colour mostly orange	***R.spectabilis***
–	Terminal cells near the pileus centre subulate, pileocystidia with appendages, pileus colour bright yellow	***R.sublaevis***

## ﻿Discussion

### ﻿Nomenclature

In the context of recent phylogenetic studies, infrageneric classifications of the genus Russulawere elaborated on a large scale atsubgenusrank (Buyck et al. 2020, 2024) or on a small scale at subsection rank (Adamčík et al. 2019; Wang et al. 2019a; Rossi et al. 2020; Looney et al. 2022). However, a recent concept for an infrageneric classification at section level rank is still lacking and, therefore, the placement of “Afrovirescentinae” withinsect.Heterophyllae is preliminary. Members of the “*Afrovirescentinae*” clade belong to Russulasubgen.Heterophyllidiae and are sister to the subsect.Virescentinae. They may be treated as part of *Virescentinae* because they collectively form a monophyletic and supported lineage, but we refrain from including them into this subsection because they have a distinct morphology and area of distribution. “*Afrovirescentinae*” have basidiospores which are predominantly subglobose with a completely reticulated ornamentation and they occur in tropical Africa and lowland tropical rainforests in South and Central America, while the basidiospores of *Virescentinae* are predominantly ellipsoid with a more isolated ornamentation and they occur in temperate to subtropical areas of Europe, Asia and North America. In our opinion, the “*Afrovirescentinae*” clade deserves the rank of a subsection, but its nomenclature is not resolved yet, due to older described subsection names which represent or may represent this phylogenetic group. In this study, we demonstrated that “*Afrovirescentinae*” species are represented by a variable field aspect and micromorphology. These distinct morphological differences within “*Afrovirescentinae*” apparently caused recognition of multiple subsections defined by morphology which cover different parts of this phylogenetic group. Our type studies of subsections *Inflatinae* and *Pseudoepitheliosinae* (Buyck 1990a; Buyck 1990b) revealed that at least these two already-described subsections fall into the “*Afrovirescentinae*” clade. It is possible that more previously described names (e.g. subsect. ParadermatinaeBuyck orsubsect.Parvoroseinae Buyck) will be competing with these two subsection names by increased sampling efforts of type material. For this reason, we do not use the currently oldest available subsection name *Inflatinae* to include all members of the “*Afrovirescentinae*” clade.

### ﻿Distribution

Several species of *Boletaceae* and *Inocybaceae* are known to have large distribution areas extending from Sudanian savannah woodlands in Benin to the miombo woodlands of the Zambezian biogeographic region (Badou et al. 2018; Han et al. 2018; Aïgnon et al. 2023). Our analyses of publicly available sequence data revealed this distribution pattern also for several *Russula* species in “*Afrovirescentinae*” clade. While these species follow well the distribution of the mesic savannah biome, some “*Afrovirescentinae*” species occurring in *Gilbertiodendron* J. Léonard forests in Cameroon and Gabon are broadly distributed across the Guineo-Congolian Rain Forest Biome (Huntley 2023). We expect an overlap of the distribution areas of species from these two biomes in the adjacent intermixed transitional mosaics. Within the mesic savannah biome, Azihou et al. (2013) found non-overlapping distributions of savannah and gallery forest tree species across savannah–forest boundaries in northern Benin. This general trend was also confirmed for *Russula* species in “*Afrovirescentinae*”, which are usually restricted to an occurrence in either gallery forests or savannah woodlands.

“*Afrovirescentinae*” sequence data originated almost exclusively from tropical areas of sub-Saharan Africa and the Neotropics. Two individual sequences from New Zealand and Mexico, which cluster in “*Afrovirescentinae*”, stand out due to their origin from temperate regions.

It seems that climate preferences and distribution of the sister lineage, subsect.Virescentinae, are only marginally overlapping. *Virescentinae* are well represented in temperate and subtropical areas of North America and Southeast Asia. In sub-Saharan Africa, *Virescentinae* are absent, while “*Afrovirescentinae*” are not detected in North Africa. Several new, recently described species from South and Southeast Asia, i.e. from China (Das et al. 2017; Song et al. 2018; Hyde et al. 2019; Chen et al. 2021), India (Dutta et al. 2015), Pakistan (Ullah et al. 2020) and Thailand (Paloi et al. 2023), suggest that this area is a diversity hotspot for species of *Virescentinae*. Additionally, in North America, a high diversity of *Virescentinae* species is reported (Buyck and Adamčík 2011, https://www2.muse.it/russulales-news/ accessed on 18.07.2024). Species of “*Afrovirescentinae*” are absent from both areas. Due to insufficient recent sequence data from Central and South America we cannot confirm if there is a climatic or geographical gap in distribution of these two sister phylogenetic groups. Based on our data, we assume that the evolutionary history and distribution of species of *Virescentinae* and “*Afrovirescentinae*” results from adaptations to different climates and ancestral origins of these sister phylogenetic groups.

### ﻿Estimation of species richness

The estimation of fungal species diversity in insufficiently explored areas is a challenging task and an important measure for assessing the world’s undescribed fungal species richness (Niskanen et al. 2023). According to our analysis of publicly available data, only approximately nine species belonging to “*Afrovirescentinae*” were used in previous phylogenetic analyses (Buyck et al. 2018; Wang et al. 2019a). Sequences of these species formed a well-supported unlabelled clade in subgen.Heterophyllidiae. In the present study, we uncovered 94, that is roughly ten times the number of species in the “*Afrovirescentinae*” clade, mainly based on the analysis of high throughput metabarcoding data from environmental DNA (eDNA) deposited in the UNITE database (https://unite.ut.ee/) (Nilsson et al. 2018; Abarenkov et al. 2024). Furthermore, we increased the number of known *Russula* species for West Africa from 51 to 58 species and for Benin from 10 to 20 species (Piepenbring et al. 2020). *Russulahiemisilvae* was suspected to occur in Senegal (Härkönen et al. 1993). In the present study, however, we provide the first reliable record of the species for West Africa. *Russulasublaevis* was the only previously described species of “*Afrovirescentinae*” that was already reported from West Africa (Togo; Kamou et al. (2017)). The overall high number of singletons in the “*Afrovirescentinae*” ITS phylogeny, retrieved by both eDNA and basidiomata samples, indicates a high diversity of still undiscovered species. The results of the present study underline the effectiveness of integrative studies combining voucher-based and environmental sequence data (Truong et al. 2017), especially in such understudied areas as Benin (Kaygusuz et al. 2024).

### ﻿Evolutionary habitat adaptations

Morphological characteristics of fungal fruiting bodies can sometimes be linked to specific environmental factors suggesting a functionality that results in evolutionary advantages. For example, according to Bässler et al. (2021), mushrooms are larger in areas characterised by a high seasonality and an intermediate mean temperature. Krah et al. (2019) showed that they are significantly darker in areas with cold climates. The basidiomata of Beninese species of “*Afrovirescentinae*” are large and fleshy in open savannah woodlands and small and ephemerous in gallery forests. Fungi have a short lifespan and reproduce by many small basidiomata in disturbed environments (Halbwachs and Bässler 2015). A small size allows a rapid formation of basidiomata which is advantageous for growth on the banks of rivers in gallery forests that are frequently flooded during the fruiting period in the rainy seasons. Similar findings of fruiting body size reduction were observed by Piepenbring et al. (2015) who investigated the fungal phenology in a secondary dry-seasonal forest in the lowlands of Panama characterised by an air humidity over 90% during rainy seasons and found that numerous species of *Agaricales* formed small ephemerous basidiomata that were increasingly mechanically damaged, covered by moulds or penetrated by insects during periods of frequent heavy rains. Our findings are also in agreement with De Crop et al. (2018) who noticed small basidioma sizes of *Lactifluus* (Pers.) Roussel species in tropical forests of Thailand and assumed that it represents an advantage in conditions where fungal tissues are more susceptible to rotting or damage. In the dense vegetation of gallery forests, the high air humidity makes a development of basidiomata with thin flesh possible without drying out. Sturdy thick-fleshed and large basidiomata keep the hymenium moist for a long enough period to allow basidiospore formation in savannah woodlands with more open vegetation and soils exposed to wind and sunlight (Halbwachs et al. 2016). During our fieldwork in Benin, we also observed a similar trend in the stature of basidiomata in other lineages of ECM fungi, for example, *Amanita* Pers. and *Boletaceae*. Our observations support the hypothesis that basidioma size may be an adaptive trait (Bässler et al. 2021).

The basidiospore ornamentation is another morphological trait showing differences in Beninese “*Afrovirescentinae*” between studied habitats. It was very low (0.1–0.5 µm) for the majority of species in savannah woodlands and distinctly higher (1.0–2.6 µm) for species in gallery forests. Smooth spores are typical for arid areas where wind is the predominant vector for dispersal (Kreisel & Al‐Fatimi 2008). The almost smooth ornamentation of the basidiospores observed in some species in savannah woodlands is a rare characteristic for *Russula* species which may be a result to an adaptation to the environmental conditions in that habitat. It is windless with an air humidity close to saturation in gallery forests. The investment in a more prominent hydrophobic ornamentation might represent an advantage to ensure the functionality of the basidiospore discharge mechanism via Buller’s drop since relative humidity and hydrophobicity of the spore surface influence the discharge mechanism (Stolze Rybczynski et al. 2009). Another function of spore ornamentation in ECM fungi is the facilitation of dispersal by arthropods within the soil to get close enough to uncolonised root tips (Halbwachs et al. 2015; Calhim et al. 2018). Basidiospores of savannah species were usually smaller than basidiospores of gallery forest species. Smaller spores are known to have a higher ability to disperse by air (Hussein et al. 2013; Wang et al. 2021), which, in combination with larger basidiomata producing larger numbers of basidiospores, can facilitate the dispersal of *Russula* species in open savannahs.

We demonstrate that morphological fungal traits correlate with environmental conditions even within the evolutionary relatively young and small lineage of “*Afrovirescentinae*”. In connection with contrasting ecosystems included in our study, we assume that major factors influencing these traits are climate conditions. Unfortunately, the data about environmental variables are missing for the majority of publicly-available sequences so that we were unable to trace how consistent these morphological adaptations are in “*Afrovirescentinae*” and other tropical ECM fungi outside the studied area.

## Supplementary Material

XML Treatment for Russula acrialbida

XML Treatment for Russula beenkenii

XML Treatment for Russula carmesina

XML Treatment for Russula coronata

XML Treatment for Russula florae

XML Treatment for Russula hiemisilvae

XML Treatment for Russula inflata

XML Treatment for Russula mollicula

XML Treatment for Russula spectabilis

XML Treatment for Russula sublaevis

## ﻿Additional information

### Adherence to national and international regulations

All the fungal strains used in this study have been legally obtained, respecting the Convention on Biological Diversity (Rio Convention).

### Author ORCIDs

Cathrin Manz https://orcid.org/0000-0003-0515-0334

Mario Amalfi https://orcid.org/0000-0002-1792-7828

Bart Buyck https://orcid.org/0000-0001-6824-5280

Felix Hampe https://orcid.org/0000-0001-7642-7930

Nourou S. Yorou https://orcid.org/0000-0001-6997-811X

Slavomír Adamčík https://orcid.org/0000-0003-2156-5767

Meike Piepenbring https://orcid.org/0000-0002-7043-5769

### Data availability

All of the data that support the findings of this study are available in the main text or Supplementary Information.

## Supplementary materials

Supplementary material 1 Names of *Russula* species originally described from tropical Africa, their ecology according to the original descriptions, origin of type material and references to original descriptions Data type xlsx https://binary.pensoft.net/file/1253164This dataset is made available under the Open Database License (http://opendatacommons.org/licenses/odbl/1.0/). The Open Database License (ODbL) is a license agreement intended to allow users to freely share, modify, and use this Dataset while maintaining this same freedom for others, provided that the original source and author(s) are credited.Cathrin Manz, Mario Amalfi Bart Buyck, Felix Hampe, Nourou S. Yorou, Slavomír Adamčík , Meike Piepenbring
